# Supplementary material for: The impact of hsa-miR-1972 on the expression of von Willebrand factor in breast cancer progression regulation
Source: PeerJ. 2024 Nov 8;12:e18476. doi: 10.7717/peerj.18476 (PMC11552492; doi:10.7717/peerj.18476)
Supplement: Supplemental Information 2 [file peerj-12-18476-s002.docx]

| Target Rank | Target Score | miRNA Name | Gene ID | Gene Symbol | Transcript Accession | Gene Description | |  |  |
| --- | --- | --- | --- | --- | --- | --- | --- | --- | --- |
| 1 | 98 | hsa-miR-29a-3p | 1282 | COL4A1 | NM_001845 | collagen type IV alpha 1 chain | | |  |
| 2 | 98 | hsa-miR-29c-3p | 1282 | COL4A1 | NM_001845 | collagen type IV alpha 1 chain | | |  |
| 3 | 98 | hsa-miR-29b-3p | 1282 | COL4A1 | NM_001845 | collagen type IV alpha 1 chain | | |  |
| 4 | 96 | hsa-miR-381-3p | 1282 | COL4A1 | NM_001845 | collagen type IV alpha 1 chain | | |  |
| 5 | 96 | hsa-miR-488-3p | 1282 | COL4A1 | NM_001303110 | collagen type IV alpha 1 chain | | |  |
| 6 | 95 | hsa-miR-148b-3p | 1282 | COL4A1 | NM_001845 | collagen type IV alpha 1 chain | | |  |
| 7 | 95 | hsa-miR-152-3p | 1282 | COL4A1 | NM_001845 | collagen type IV alpha 1 chain | | |  |
| 8 | 95 | hsa-miR-3065-5p | 1282 | COL4A1 | NM_001845 | collagen type IV alpha 1 chain | | |  |
| 9 | 95 | hsa-miR-148a-3p | 1282 | COL4A1 | NM_001845 | collagen type IV alpha 1 chain | | |  |
| 10 | 95 | hsa-miR-124-3p | 1282 | COL4A1 | NM_001845 | collagen type IV alpha 1 chain | | |  |
| 11 | 95 | hsa-miR-506-3p | 1282 | COL4A1 | NM_001845 | collagen type IV alpha 1 chain | | |  |
| 12 | 94 | hsa-miR-200a-5p | 1282 | COL4A1 | NM_001303110 | collagen type IV alpha 1 chain | | |  |
| 13 | 92 | hsa-miR-23a-3p | 1282 | COL4A1 | NM_001845 | collagen type IV alpha 1 chain | | |  |
| 14 | 92 | hsa-miR-23b-3p | 1282 | COL4A1 | NM_001845 | collagen type IV alpha 1 chain | | |  |
| 15 | 91 | hsa-miR-224-5p | 1282 | COL4A1 | NM_001845 | collagen type IV alpha 1 chain | | |  |
| 16 | 90 | hsa-miR-3152-3p | 1282 | COL4A1 | NM_001845 | collagen type IV alpha 1 chain | | |  |
| 17 | 88 | hsa-let-7a-5p | 1282 | COL4A1 | NM_001845 | collagen type IV alpha 1 chain | | |  |
| 18 | 88 | hsa-miR-202-3p | 1282 | COL4A1 | NM_001845 | collagen type IV alpha 1 chain | | |  |
| 19 | 88 | hsa-let-7g-5p | 1282 | COL4A1 | NM_001845 | collagen type IV alpha 1 chain | | |  |
| 20 | 88 | hsa-let-7i-5p | 1282 | COL4A1 | NM_001845 | collagen type IV alpha 1 chain | | |  |
| 21 | 88 | hsa-let-7b-5p | 1282 | COL4A1 | NM_001845 | collagen type IV alpha 1 chain | | |  |
| 22 | 88 | hsa-let-7e-5p | 1282 | COL4A1 | NM_001845 | collagen type IV alpha 1 chain | | |  |
| 23 | 88 | hsa-let-7c-5p | 1282 | COL4A1 | NM_001845 | collagen type IV alpha 1 chain | | |  |
| 24 | 88 | hsa-miR-98-5p | 1282 | COL4A1 | NM_001845 | collagen type IV alpha 1 chain | | |  |
| 25 | 88 | hsa-let-7f-5p | 1282 | COL4A1 | NM_001845 | collagen type IV alpha 1 chain | | |  |
| 26 | 88 | hsa-miR-495-3p | 1282 | COL4A1 | NM_001845 | collagen type IV alpha 1 chain | | |  |
| 27 | 88 | hsa-let-7d-5p | 1282 | COL4A1 | NM_001845 | collagen type IV alpha 1 chain | | |  |
| 28 | 85 | hsa-miR-628-5p | 1282 | COL4A1 | NM_001845 | collagen type IV alpha 1 chain | | |  |
| 29 | 85 | hsa-miR-369-3p | 1282 | COL4A1 | NM_001845 | collagen type IV alpha 1 chain | | |  |
| 30 | 85 | hsa-miR-4520-3p | 1282 | COL4A1 | NM_001845 | collagen type IV alpha 1 chain | | |  |
| 31 | 85 | hsa-miR-205-5p | 1282 | COL4A1 | NM_001303110 | collagen type IV alpha 1 chain | | |  |
| 32 | 84 | hsa-miR-767-5p | 1282 | COL4A1 | NM_001845 | collagen type IV alpha 1 chain | | |  |
| 33 | 84 | hsa-miR-637 | 1282 | COL4A1 | NM_001845 | collagen type IV alpha 1 chain | | |  |
| 34 | 83 | hsa-miR-545-3p | 1282 | COL4A1 | NM_001845 | collagen type IV alpha 1 chain | | |  |
| 35 | 83 | hsa-miR-605-3p | 1282 | COL4A1 | NM_001303110 | collagen type IV alpha 1 chain | | |  |
| 36 | 79 | hsa-miR-374a-5p | 1282 | COL4A1 | NM_001845 | collagen type IV alpha 1 chain | | |  |
| 37 | 78 | hsa-miR-377-3p | 55052 | MRPL20 | NM_017971 | mitochondrial ribosomal protein L20 | | | |
| 38 | 77 | hsa-miR-1972 | 633 | BGN | NM_001711 | biglycan |  |  |  |
| 39 | 77 | hsa-miR-374b-5p | 1282 | COL4A1 | NM_001845 | collagen type IV alpha 1 chain | | |  |
| 40 | 77 | hsa-miR-1323 | 1282 | COL4A1 | NM_001303110 | collagen type IV alpha 1 chain | | |  |
| 41 | 76 | hsa-miR-548e-3p | 55052 | MRPL20 | NM_017971 | mitochondrial ribosomal protein L20 | | | |
| 42 | 76 | hsa-miR-221-5p | 55052 | MRPL20 | NM_001318485 | mitochondrial ribosomal protein L20 | | | |
| 43 | 76 | hsa-miR-548f-3p | 55052 | MRPL20 | NM_017971 | mitochondrial ribosomal protein L20 | | | |
| 44 | 76 | hsa-miR-548a-3p | 55052 | MRPL20 | NM_017971 | mitochondrial ribosomal protein L20 | | | |
| 45 | 74 | hsa-miR-1972 | 7450 | VWF | NM_000552 | von Willebrand factor | | |  |
| 46 | 73 | hsa-miR-374b-3p | 1282 | COL4A1 | NM_001845 | collagen type IV alpha 1 chain | | |  |
| 47 | 72 | hsa-miR-548l | 55052 | MRPL20 | NM_017971 | mitochondrial ribosomal protein L20 | | | |
| 48 | 71 | hsa-miR-655-3p | 1282 | COL4A1 | NM_001845 | collagen type IV alpha 1 chain | | |  |
| 49 | 71 | hsa-miR-374c-5p | 1282 | COL4A1 | NM_001845 | collagen type IV alpha 1 chain | | |  |
| 50 | 70 | hsa-miR-3138 | 55052 | MRPL20 | NM_001318485 | mitochondrial ribosomal protein L20 | | | |
| 51 | 70 | hsa-miR-3130-5p | 1282 | COL4A1 | NM_001303110 | collagen type IV alpha 1 chain | | |  |
| 52 | 69 | hsa-miR-4667-5p | 1282 | COL4A1 | NM_001845 | collagen type IV alpha 1 chain | | |  |
| 53 | 69 | hsa-miR-182-5p | 1282 | COL4A1 | NM_001845 | collagen type IV alpha 1 chain | | |  |
| 54 | 69 | hsa-miR-1185-2-3p | 1282 | COL4A1 | NM_001845 | collagen type IV alpha 1 chain | | |  |
| 55 | 69 | hsa-miR-1185-1-3p | 1282 | COL4A1 | NM_001845 | collagen type IV alpha 1 chain | | |  |
| 56 | 68 | hsa-miR-106a-5p | 1282 | COL4A1 | NM_001845 | collagen type IV alpha 1 chain | | |  |
| 57 | 68 | hsa-miR-519d-3p | 1282 | COL4A1 | NM_001845 | collagen type IV alpha 1 chain | | |  |
| 58 | 68 | hsa-miR-93-5p | 1282 | COL4A1 | NM_001845 | collagen type IV alpha 1 chain | | |  |
| 59 | 68 | hsa-miR-20b-5p | 1282 | COL4A1 | NM_001845 | collagen type IV alpha 1 chain | | |  |
| 60 | 68 | hsa-miR-17-5p | 1282 | COL4A1 | NM_001845 | collagen type IV alpha 1 chain | | |  |
| 61 | 68 | hsa-miR-106b-5p | 1282 | COL4A1 | NM_001845 | collagen type IV alpha 1 chain | | |  |
| 62 | 68 | hsa-miR-20a-5p | 1282 | COL4A1 | NM_001845 | collagen type IV alpha 1 chain | | |  |
| 63 | 67 | hsa-miR-183-3p | 1282 | COL4A1 | NM_001845 | collagen type IV alpha 1 chain | | |  |
| 64 | 67 | hsa-miR-548o-3p | 1282 | COL4A1 | NM_001303110 | collagen type IV alpha 1 chain | | |  |
| 65 | 66 | hsa-miR-624-5p | 1282 | COL4A1 | NM_001845 | collagen type IV alpha 1 chain | | |  |
| 66 | 65 | hsa-miR-1827 | 1282 | COL4A1 | NM_001303110 | collagen type IV alpha 1 chain | | |  |
| 67 | 64 | hsa-miR-153-3p | 1282 | COL4A1 | NM_001303110 | collagen type IV alpha 1 chain | | |  |
| 68 | 64 | hsa-miR-548k | 1282 | COL4A1 | NM_001845 | collagen type IV alpha 1 chain | | |  |
| 69 | 63 | hsa-miR-24-3p | 55052 | MRPL20 | NM_001318485 | mitochondrial ribosomal protein L20 | | | |
| 70 | 63 | hsa-miR-186-5p | 1282 | COL4A1 | NM_001845 | collagen type IV alpha 1 chain | | |  |
| 71 | 63 | hsa-miR-423-5p | 633 | BGN | NM_001711 | biglycan |  |  |  |
| 72 | 62 | hsa-miR-377-3p | 1282 | COL4A1 | NM_001845 | collagen type IV alpha 1 chain | | |  |
| 73 | 61 | hsa-miR-548t-3p | 1282 | COL4A1 | NM_001845 | collagen type IV alpha 1 chain | | |  |
