# Supplementary material for: The impact of hsa-miR-1972 on the expression of von Willebrand factor in breast cancer progression regulation
Source: PeerJ. 2024 Nov 8;12:e18476. doi: 10.7717/peerj.18476 (PMC11552492; doi:10.7717/peerj.18476)
Supplement: Supplemental Information 3 [file peerj-12-18476-s003.zip › 1_Analysis/1_WGCNA/fig1e.pdf]

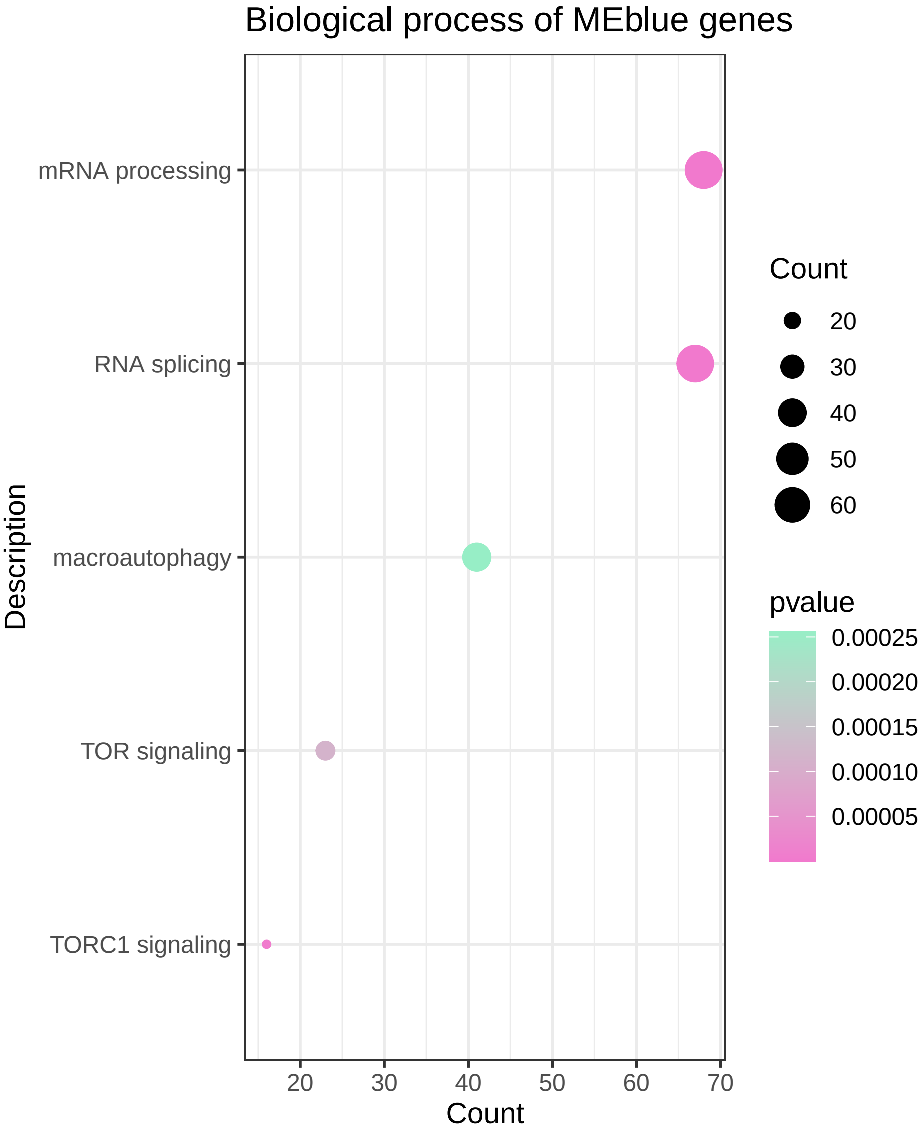
Biological process of MEblue genes
mRNA processing
RNA splicing
macroautophagy
TOR signaling
TORC1 signaling
Description
20
30
40
50
60
70
Count
Count
20
30
40
50
60
pvalue
0.00025
0.00020
0.00015
0.00010
0.00005
