# Supplementary material for: The impact of hsa-miR-1972 on the expression of von Willebrand factor in breast cancer progression regulation
Source: PeerJ. 2024 Nov 8;12:e18476. doi: 10.7717/peerj.18476 (PMC11552492; doi:10.7717/peerj.18476)
Supplement: Supplemental Information 3 [file peerj-12-18476-s003.zip › 1_Analysis/1_WGCNA/figs1a.pdf]

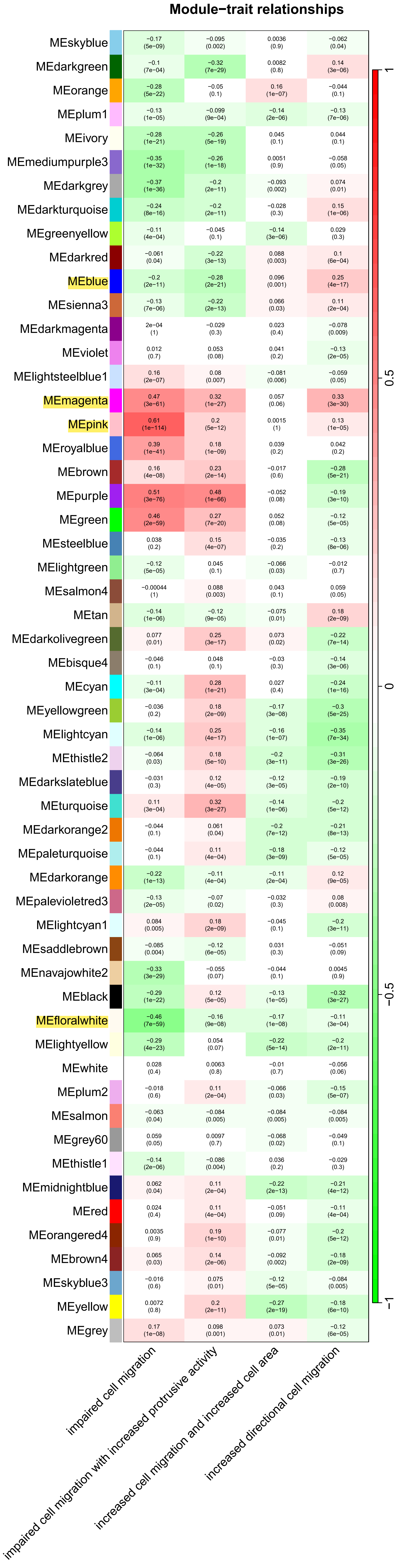
Module−trait relationships
MEskyblue −0.17 (5e−09) −0.095 (0.002) 0.0036 (0.9) −0.062 (0.04)
MEdarkgreen −0.1 (7e−04) −0.32 (7e−29) 0.0082 (0.8) 0.14 (3e−06)
MEorange −0.28 (5e−22) −0.05 (0.1) 0.16 (1e−07) −0.044 (0.1)
MEplum1 −0.13 (1e−05) −0.099 (9e−04) −0.14 (2e−06) −0.13 (7e−06)
MEivory −0.28 (1e−21) −0.26 (5e−19) 0.045 (0.1) 0.044 (0.1)
MEmediumpurple3 −0.35 (1e−32) −0.26 (1e−18) 0.0051 (0.9) −0.058 (0.05)
MEdarkgrey −0.37 (1e−36) −0.2 (2e−11) −0.093 (0.002) 0.074 (0.01)
MEdarkturquoise −0.24 (8e−16) −0.2 (2e−11) −0.028 (0.3) 0.15 (1e−06)
MEgreenyellow −0.11 (4e−04) −0.045 (0.1) −0.14 (3e−06) 0.029 (0.3)
MEdarkred −0.061 (0.04) −0.22 (3e−13) 0.088 (0.003) 0.1 (6e−04)
MEblue −0.2 (2e−11) −0.28 (2e−21) 0.096 (0.001) 0.25 (4e−17)
MEsienna3 −0.13 (7e−06) −0.22 (2e−13) 0.066 (0.03) 0.11 (2e−04)
MEdarkmagenta 2e−04 (1) −0.029 (0.3) 0.023 (0.4) −0.078 (0.009)
MEviolet 0.012 (0.7) 0.053 (0.08) 0.041 (0.2) −0.13 (2e−05)
MElightsteelblue1 0.16 (2e−07) 0.08 (0.007) −0.081 (0.006) −0.059 (0.05)
MEmagenta 0.47 (3e−61) 0.32 (1e−27) 0.057 (0.06) 0.33 (3e−30)
MEpink 0.61 (1e−114) 0.2 (5e−12) 0.0015 (1) 0.13 (1e−05)
MEroyalblue 0.39 (1e−41) 0.18 (1e−09) 0.039 (0.2) 0.042 (0.2)
MEbrown 0.16 (4e−08) 0.23 (2e−14) −0.017 (0.6) −0.28 (5e−21)
MEpurple 0.51 (3e−76) 0.48 (1e−66) −0.052 (0.08) −0.19 (3e−10)
MEgreen 0.46 (2e−59) 0.27 (7e−20) 0.052 (0.08) −0.12 (5e−05)
MEsteelblue 0.038 (0.2) 0.15 (4e−07) −0.035 (0.2) −0.13 (8e−06)
MElightgreen −0.12 (5e−05) 0.045 (0.1) −0.066 (0.03) −0.012 (0.7)
MEsalmon4 −0.00044 (1) 0.088 (0.003) 0.043 (0.1) 0.059 (0.05)
MEtan −0.14 (1e−06) −0.12 (9e−05) −0.075 (0.01) 0.18 (2e−09)
MEdarkolivegreen 0.077 (0.01) 0.25 (3e−17) 0.073 (0.02) −0.22 (7e−14)
MEbisque4 −0.046 (0.1) 0.048 (0.1) −0.03 (0.3) −0.14 (3e−06)
MEcyan −0.11 (3e−04) 0.28 (1e−21) 0.027 (0.4) −0.24 (1e−16)
MEyellowgreen −0.036 (0.2) 0.18 (2e−09) −0.17 (3e−08) −0.3 (5e−25)
MElightcyan −0.14 (1e−06) 0.25 (4e−17) −0.16 (1e−07) −0.35 (7e−34)
MEthistle2 −0.064 (0.03) 0.18 (5e−10) −0.2 (3e−11) −0.31 (3e−26)
MEdarkslateblue −0.031 (0.3) 0.12 (4e−05) −0.12 (3e−05) −0.19 (2e−10)
MEturquoise 0.11 (3e−04) 0.32 (3e−27) −0.14 (1e−06) −0.2 (5e−12)
MEdarkorange2 −0.044 (0.1) 0.061 (0.04) −0.2 (7e−12) −0.21 (8e−13)
MEpaleturquoise −0.044 (0.1) 0.11 (4e−04) −0.18 (3e−09) −0.12 (5e−05)
MEdarkorange −0.22 (1e−13) −0.11 (4e−04) −0.11 (2e−04) 0.12 (9e−05)
MEpalevioletred3 −0.13 (2e−05) −0.07 (0.02) −0.032 (0.3) 0.08 (0.008)
MElightcyan1 0.084 (0.005) 0.18 (2e−09) −0.045 (0.1) −0.2 (3e−11)
MEsaddlebrown −0.085 (0.004) −0.12 (6e−05) 0.031 (0.3) −0.051 (0.09)
MEnavajowhite2 −0.33 (3e−29) −0.055 (0.07) −0.044 (0.1) 0.0045 (0.9)
MEblack −0.29 (1e−22) 0.12 (5e−05) −0.13 (1e−05) −0.32 (3e−27)
MEfloralwhite −0.46 (7e−59) −0.16 (9e−08) −0.17 (1e−08) −0.11 (3e−04)
MElightyellow −0.29 (4e−23) 0.054 (0.07) −0.22 (5e−14) −0.2 (2e−11)
MEwhite 0.028 (0.4) 0.0063 (0.8) −0.01 (0.7) −0.056 (0.06)
MEplum2 −0.018 (0.6) 0.11 (2e−04) −0.066 (0.03) −0.15 (5e−07)
MEsalmon −0.063 (0.04) −0.084 (0.005) −0.084 (0.005) −0.084 (0.005)
MEgrey60 0.059 (0.05) 0.0097 (0.7) −0.068 (0.02) −0.049 (0.1)
MEthistle1 −0.14 (2e−06) −0.086 (0.004) 0.036 (0.2) −0.029 (0.3)
MEmidnightblue 0.062 (0.04) 0.11 (2e−04) −0.22 (2e−13) −0.21 (4e−12)
MEred 0.024 (0.4) 0.11 (4e−04) −0.051 (0.09) −0.11 (4e−04)
MEorangered4 0.0035 (0.9) 0.19 (1e−10) −0.077 (0.01) −0.2 (5e−12)
MEbrown4 0.065 (0.03) 0.14 (2e−06) −0.092 (0.002) −0.18 (2e−09)
MEskyblue3 −0.016 (0.6) 0.075 (0.01) −0.12 (5e−05) −0.084 (0.005)
MEyellow 0.0072 (0.8) 0.2 (2e−11) −0.27 (2e−19) −0.18 (6e−10)
MEgrey 0.17 (1e−08) 0.098 (0.001) 0.073 (0.01) −0.12 (6e−05)
impaired cell migration
impaired cell migration with increased protrusive activity
increased cell migration and increased cell area
increased directional cell migration
1
0.5
0
−0.5
−1
