# Supplementary material for: The impact of hsa-miR-1972 on the expression of von Willebrand factor in breast cancer progression regulation
Source: PeerJ. 2024 Nov 8;12:e18476. doi: 10.7717/peerj.18476 (PMC11552492; doi:10.7717/peerj.18476)
Supplement: Supplemental Information 3 [file peerj-12-18476-s003.zip › 1_Analysis/2_surrivive_analysis/fig1e.pdf]

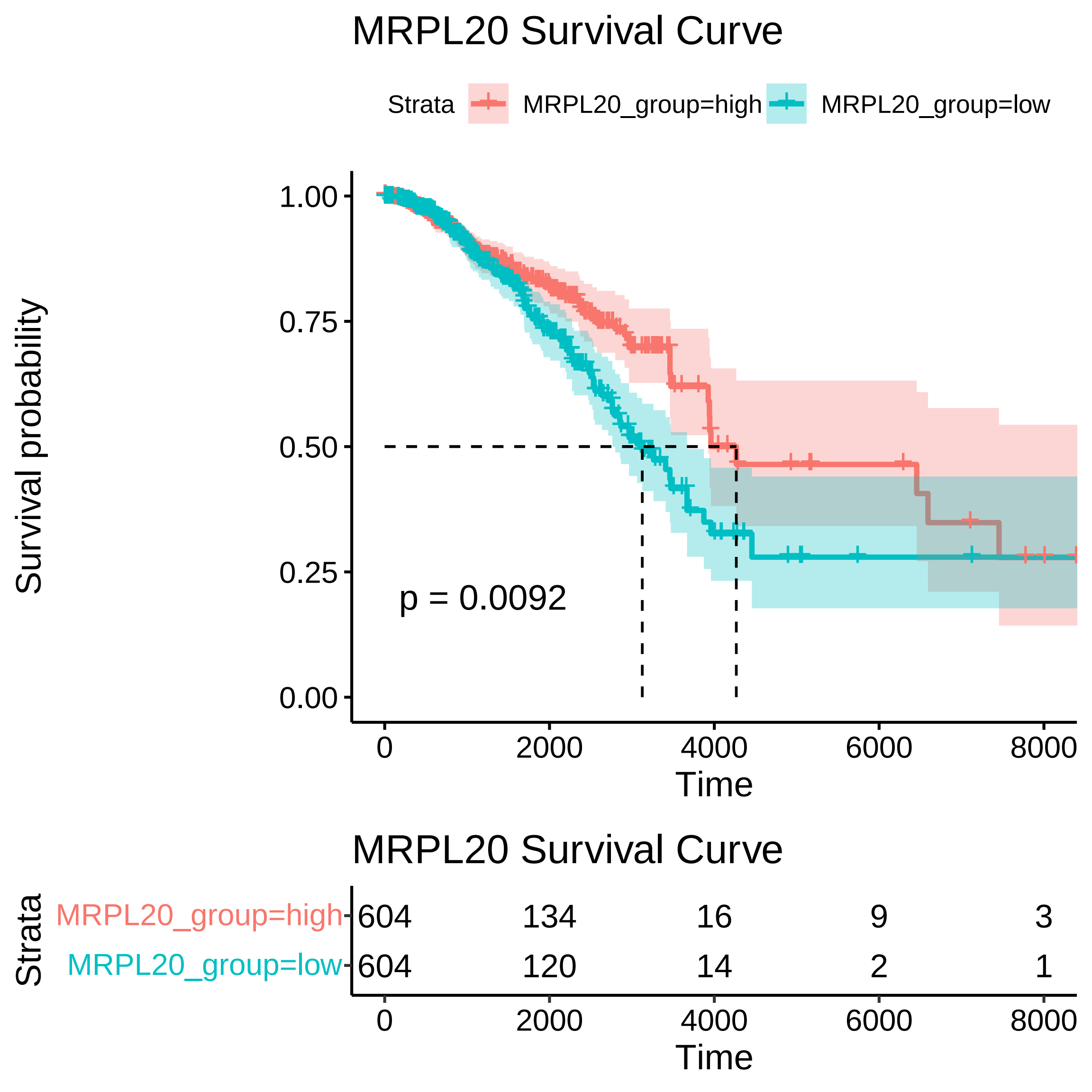
MRPL20 Survival Curve
Strata
MRPL20_group=high
MRPL20_group=low
Survival probability
1.00
0.75
0.50
0.25
0.00
p = 0.0092
0
2000
4000
6000
8000
Time
MRPL20 Survival Curve
Strata
MRPL20_group=high
MRPL20_group=low
604
134
16
9
3
604
120
14
2
1
0
2000
4000
6000
8000
Time
