# Supplementary material for: The impact of hsa-miR-1972 on the expression of von Willebrand factor in breast cancer progression regulation
Source: PeerJ. 2024 Nov 8;12:e18476. doi: 10.7717/peerj.18476 (PMC11552492; doi:10.7717/peerj.18476)
Supplement: Supplemental Information 3 [file peerj-12-18476-s003.zip › 1_Analysis/2_surrivive_analysis/Fig2.pdf]

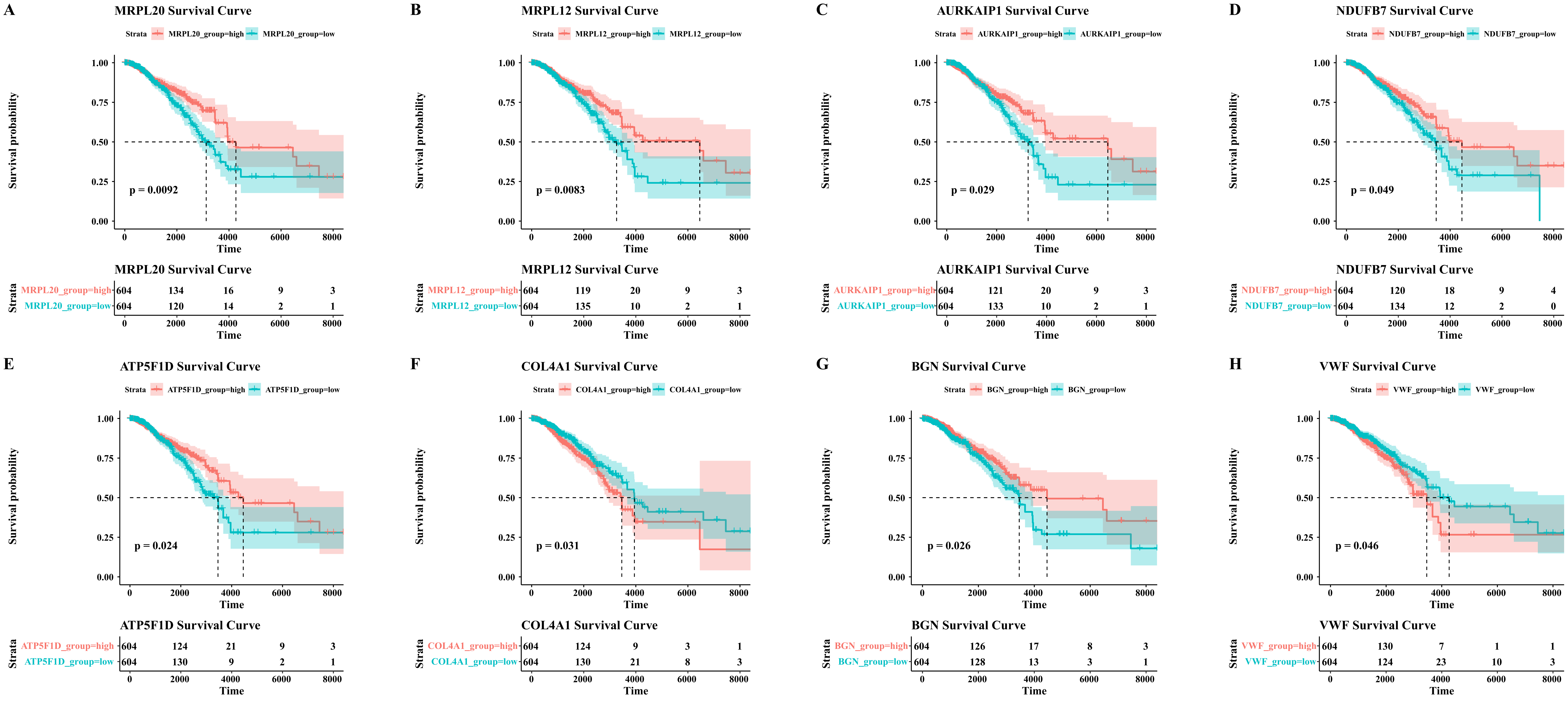
A
MRPL20 Survival Curve
Strata MRPL20_group=high MRPL20_group=low
Survival probability
Time
p = 0.0092
MRPL20 Survival Curve
Strata
MRPL20_group=high 604 134 16 9 3
MRPL20_group=low 604 120 14 2 1
B
MRPL12 Survival Curve
Strata MRPL12_group=high MRPL12_group=low
p = 0.0083
MRPL12 Survival Curve
MRPL12_group=high 604 119 20 9 3
MRPL12_group=low 604 135 10 2 1
C
AURKAIP1 Survival Curve
Strata AURKAIP1_group=high AURKAIP1_group=low
p = 0.029
AURKAIP1 Survival Curve
AURKAIP1_group=high 604 121 20 9 3
AURKAIP1_group=low 604 133 10 2 1
D
NDUFB7 Survival Curve
Strata NDUFB7_group=high NDUFB7_group=low
p = 0.049
NDUFB7 Survival Curve
NDUFB7_group=high 604 120 18 9 4
NDUFB7_group=low 604 134 12 2 0
E
ATP5F1D Survival Curve
Strata ATP5F1D_group=high ATP5F1D_group=low
p = 0.024
ATP5F1D Survival Curve
ATP5F1D_group=high 604 124 21 9 3
ATP5F1D_group=low 604 130 9 2 1
F
COL4A1 Survival Curve
Strata COL4A1_group=high COL4A1_group=low
p = 0.031
COL4A1 Survival Curve
COL4A1_group=high 604 124 9 3 1
COL4A1_group=low 604 130 21 8 3
G
BGN Survival Curve
Strata BGN_group=high BGN_group=low
p = 0.026
BGN Survival Curve
BGN_group=high 604 126 17 8 3
BGN_group=low 604 128 13 3 1
H
VWF Survival Curve
Strata VWF_group=high VWF_group=low
p = 0.046
VWF Survival Curve
VWF_group=high 604 130 7 1 1
VWF_group=low 604 124 23 10 3
0 2000 4000 6000 8000
0.00 0.25 0.50 0.75 1.00
