# Supplementary figures and images for: The impact of hsa-miR-1972 on the expression of von Willebrand factor in breast cancer progression regulation
Source: PeerJ. 2024 Nov 8;12:e18476. doi: 10.7717/peerj.18476 (PMC11552492; doi:10.7717/peerj.18476)

**A**

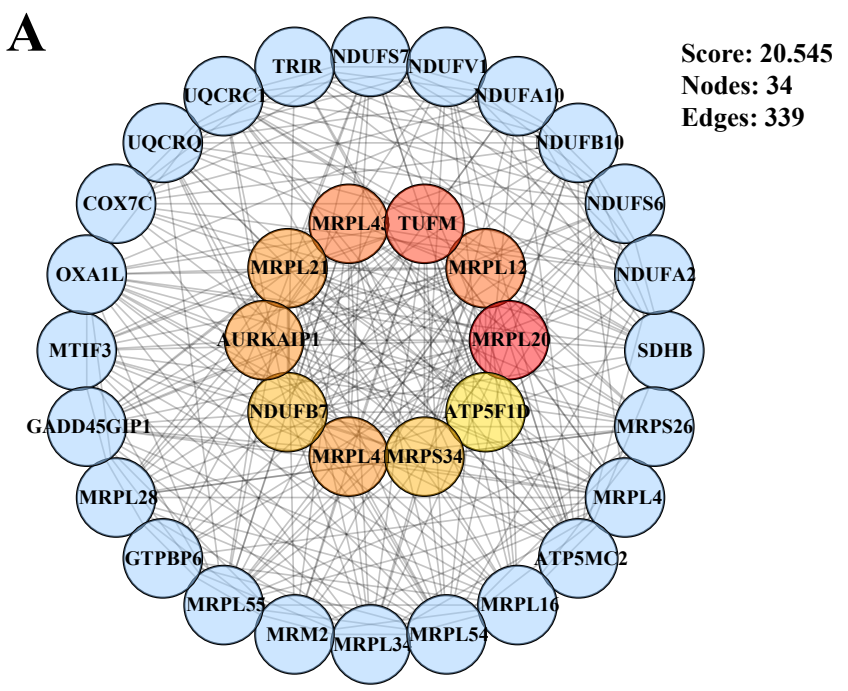

MEblue hub genes

**B**

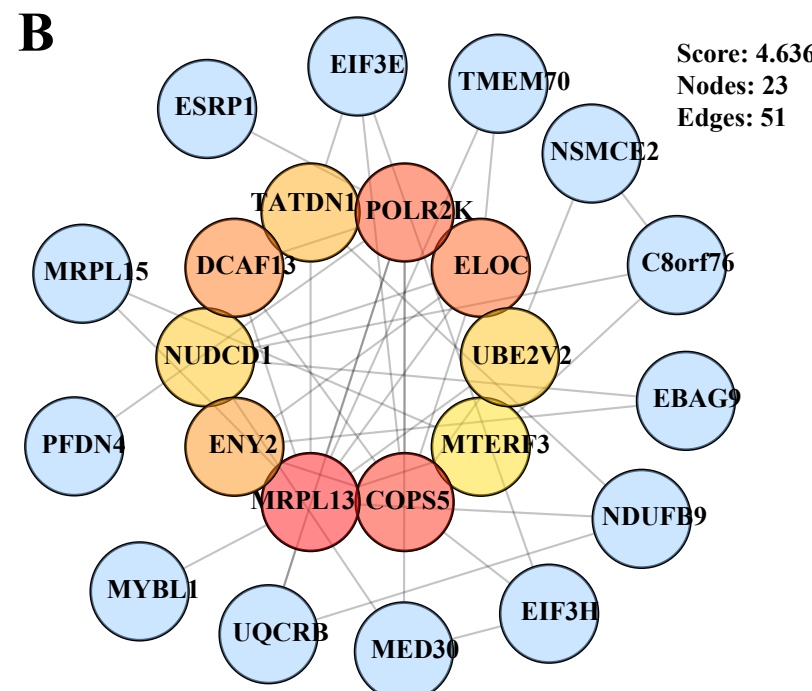

MEflorawhite hub genes

**C**

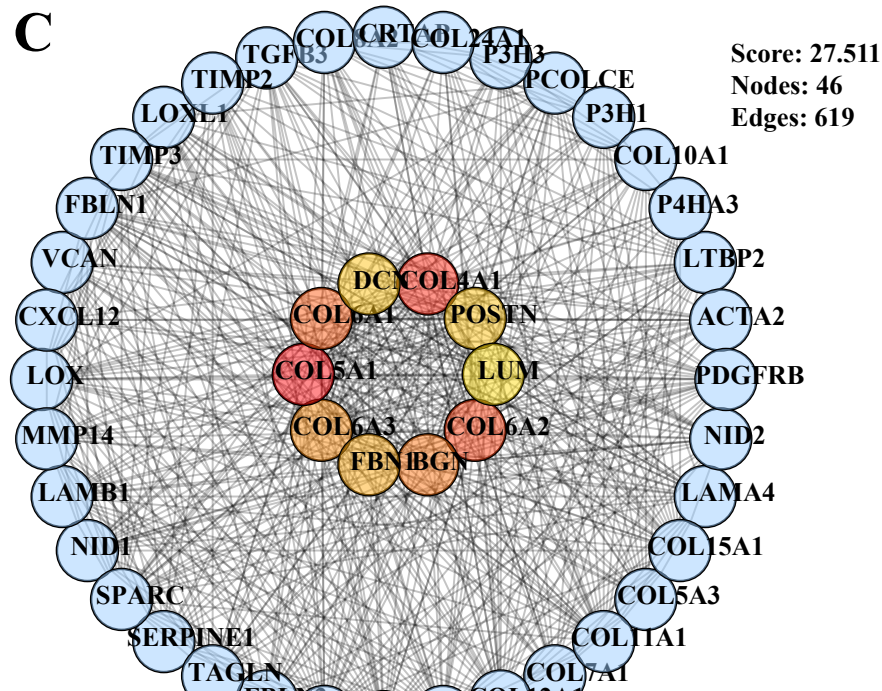

MEmagenta hub genes

**D**

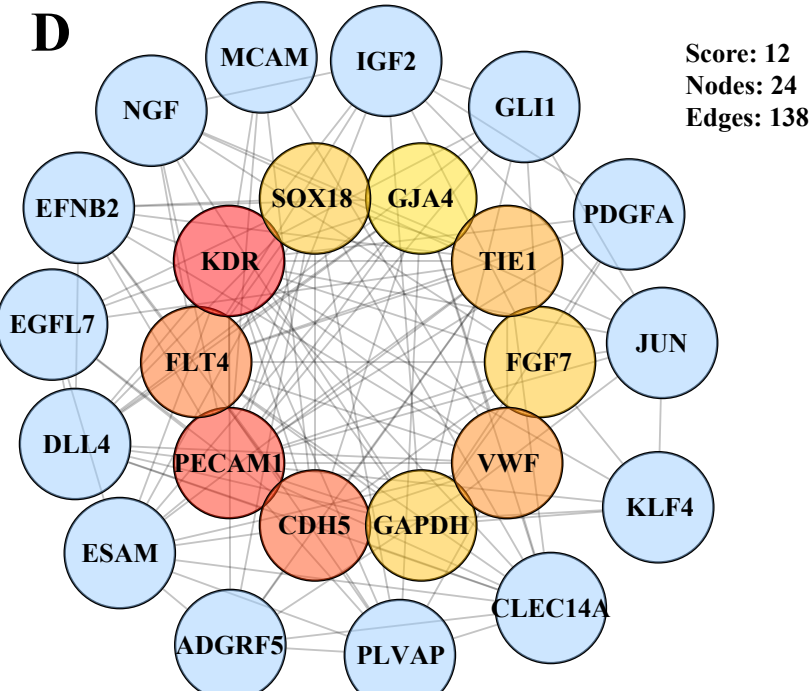

MEPink hub genes

**E**

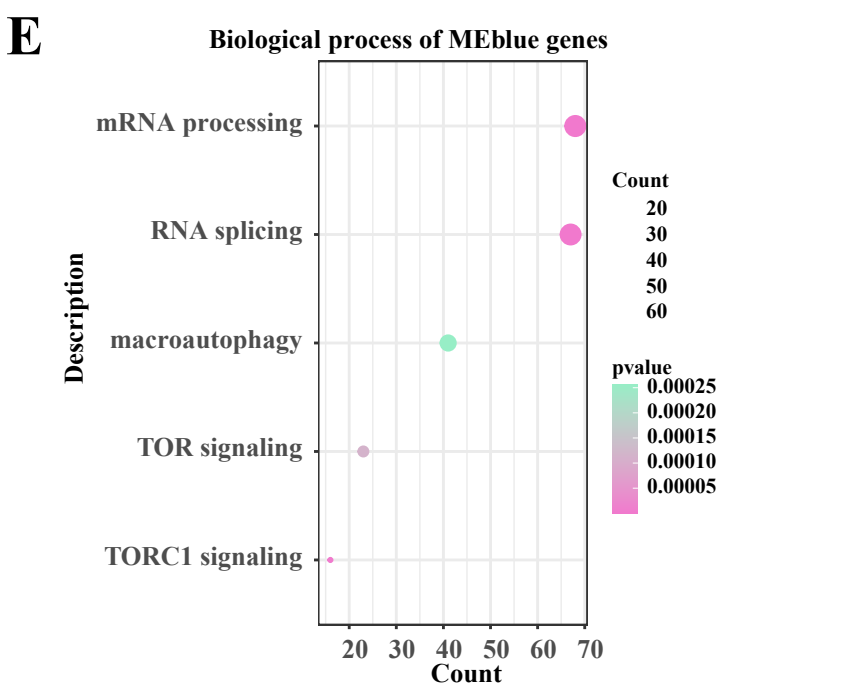

**F**

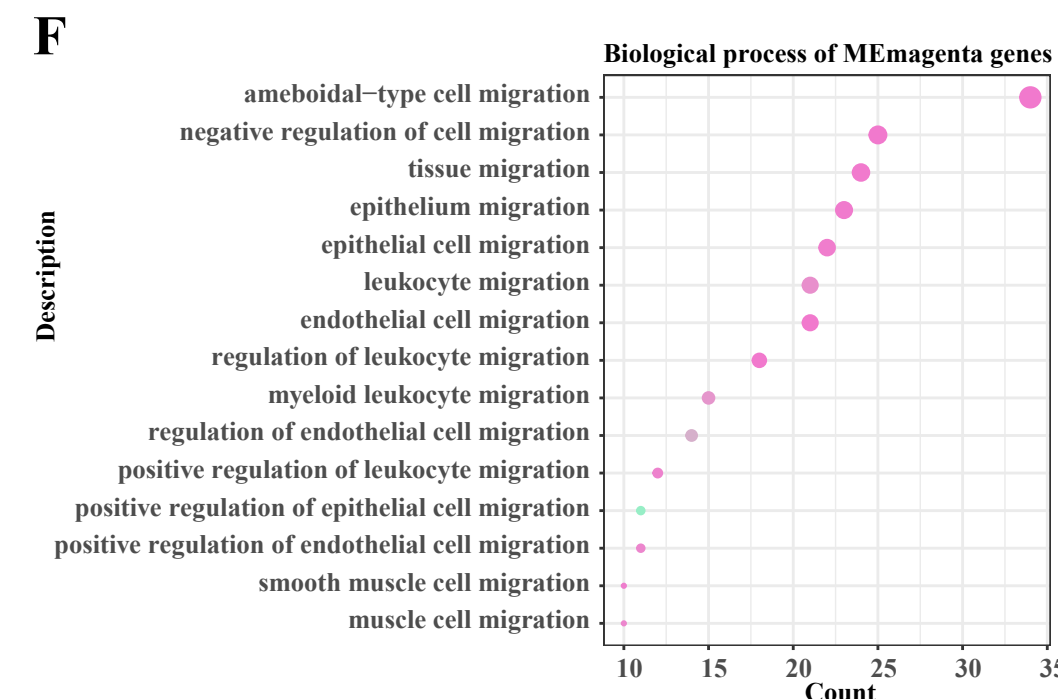

**G**

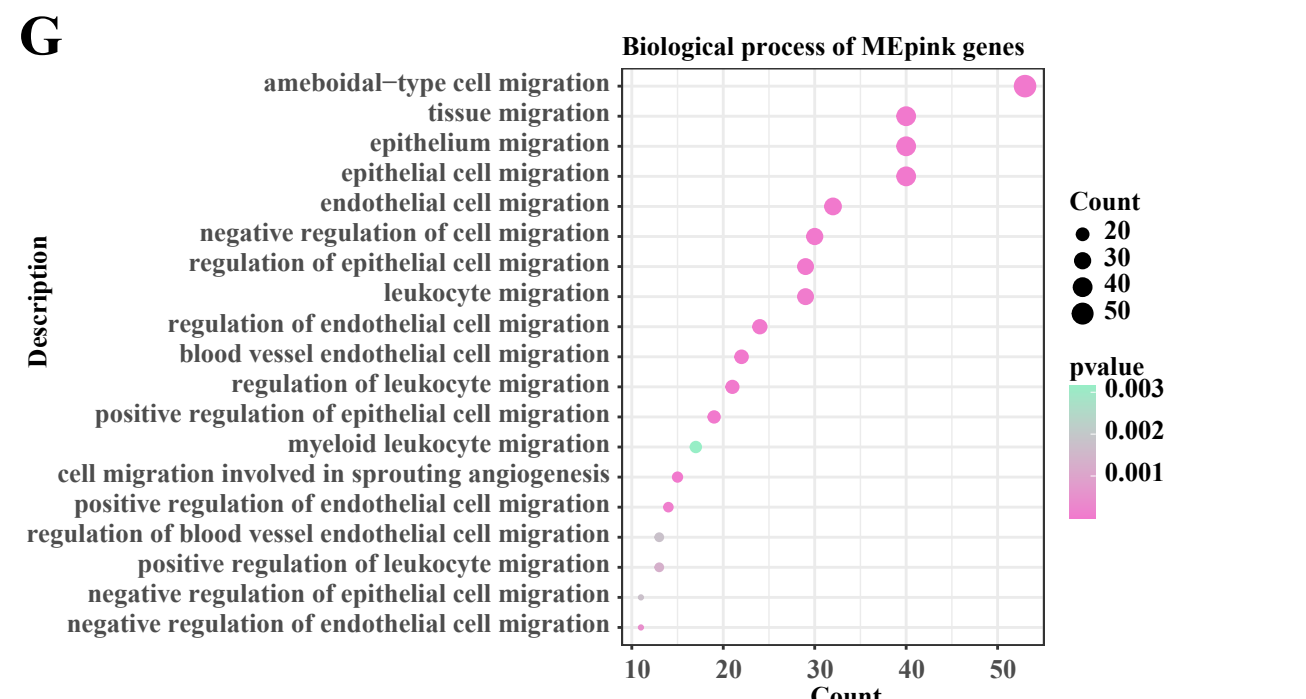

Supplement: Supplemental Information 3 [file peerj-12-18476-s003.zip › 1_Analysis/1_WGCNA/Fig1.pdf]

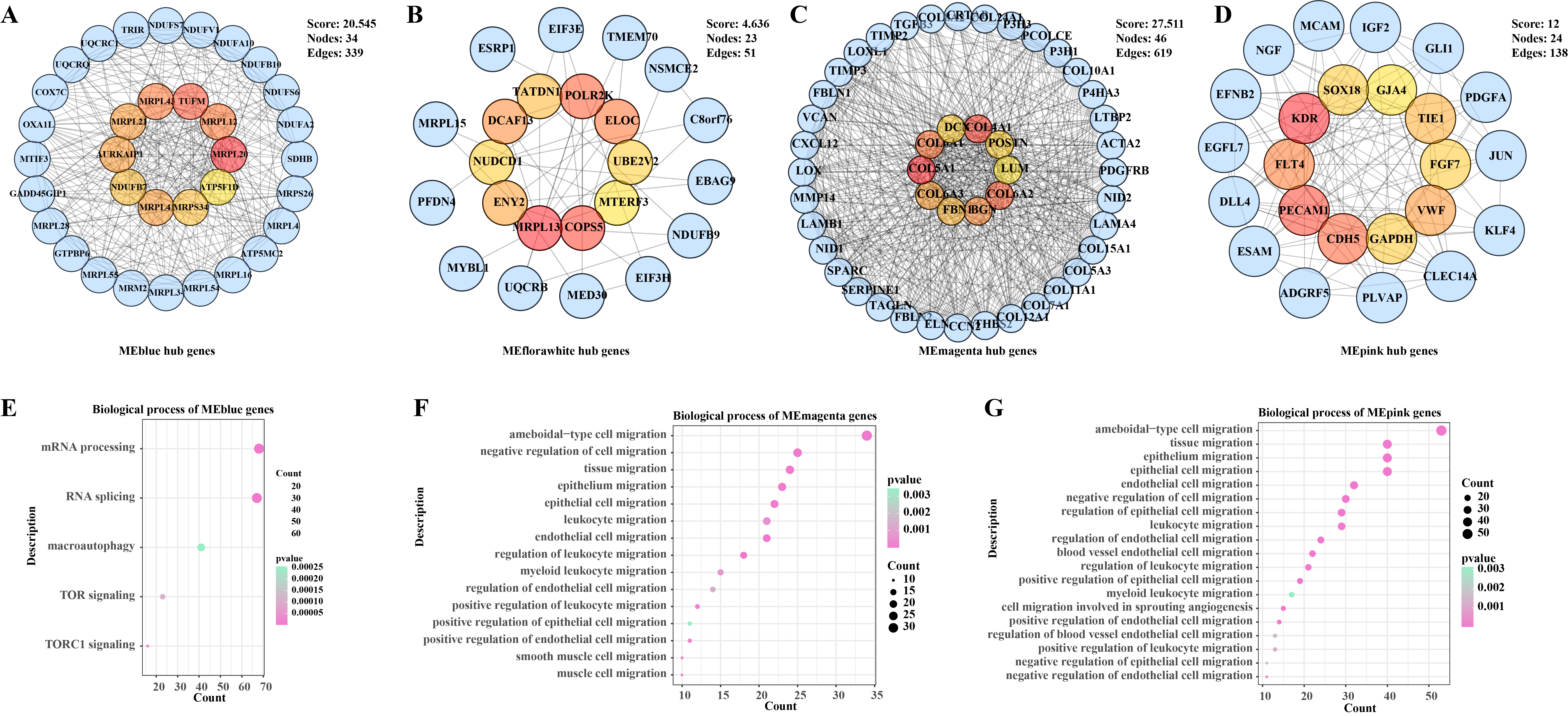

Supplement: Supplemental Information 3 [file peerj-12-18476-s003.zip › 1_Analysis/1_WGCNA/Fig1.png]

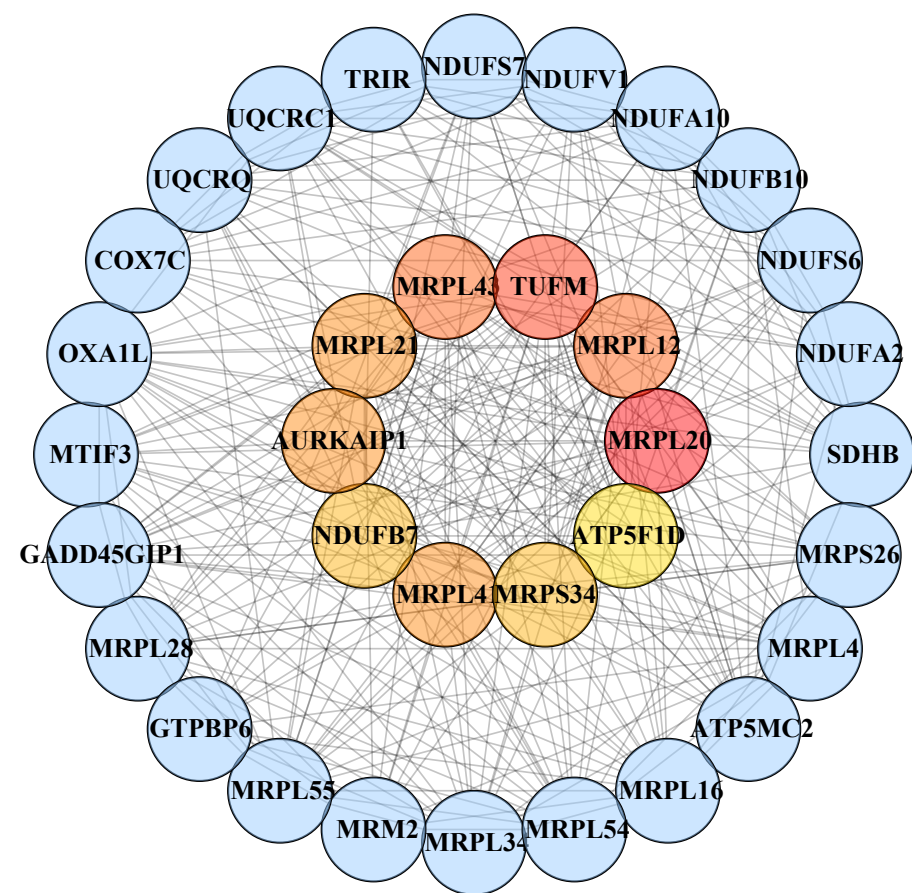

Supplement: Supplemental Information 3 [file peerj-12-18476-s003.zip › 1_Analysis/1_WGCNA/fig1a_blue_hubgene.pdf]

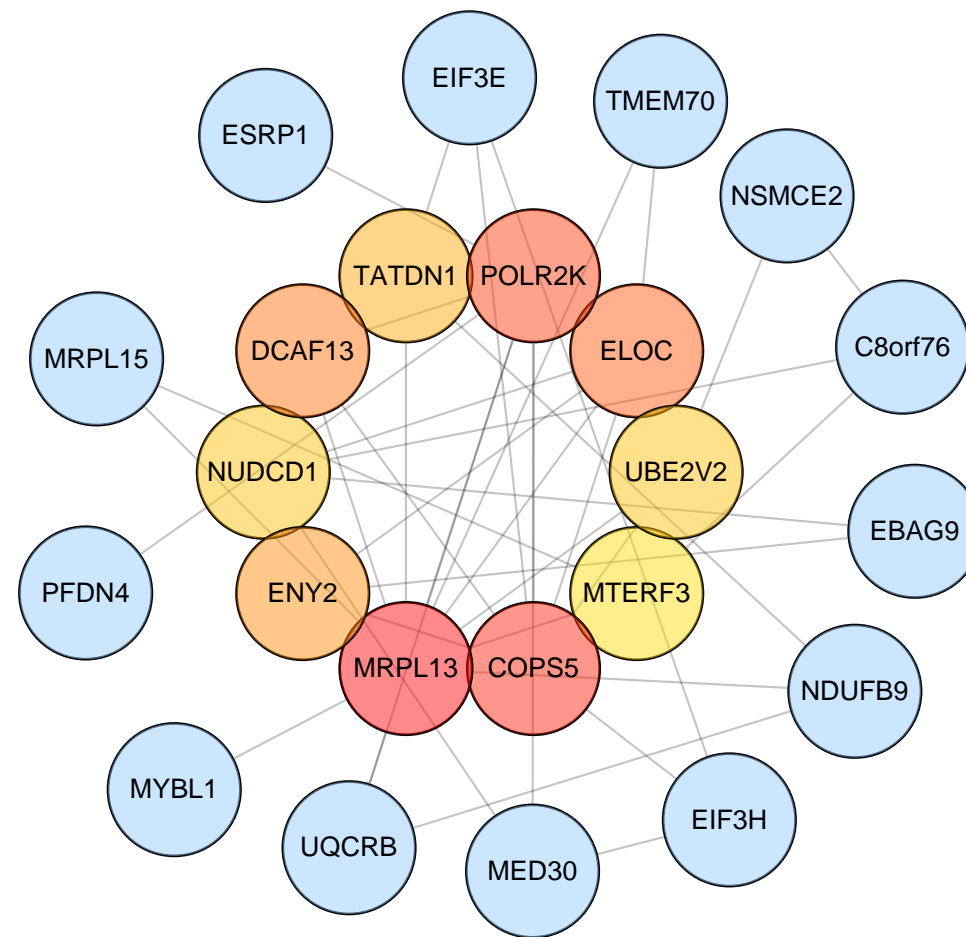

Supplement: Supplemental Information 3 [file peerj-12-18476-s003.zip › 1_Analysis/1_WGCNA/fig1b_florawhite_hubgene.pdf]

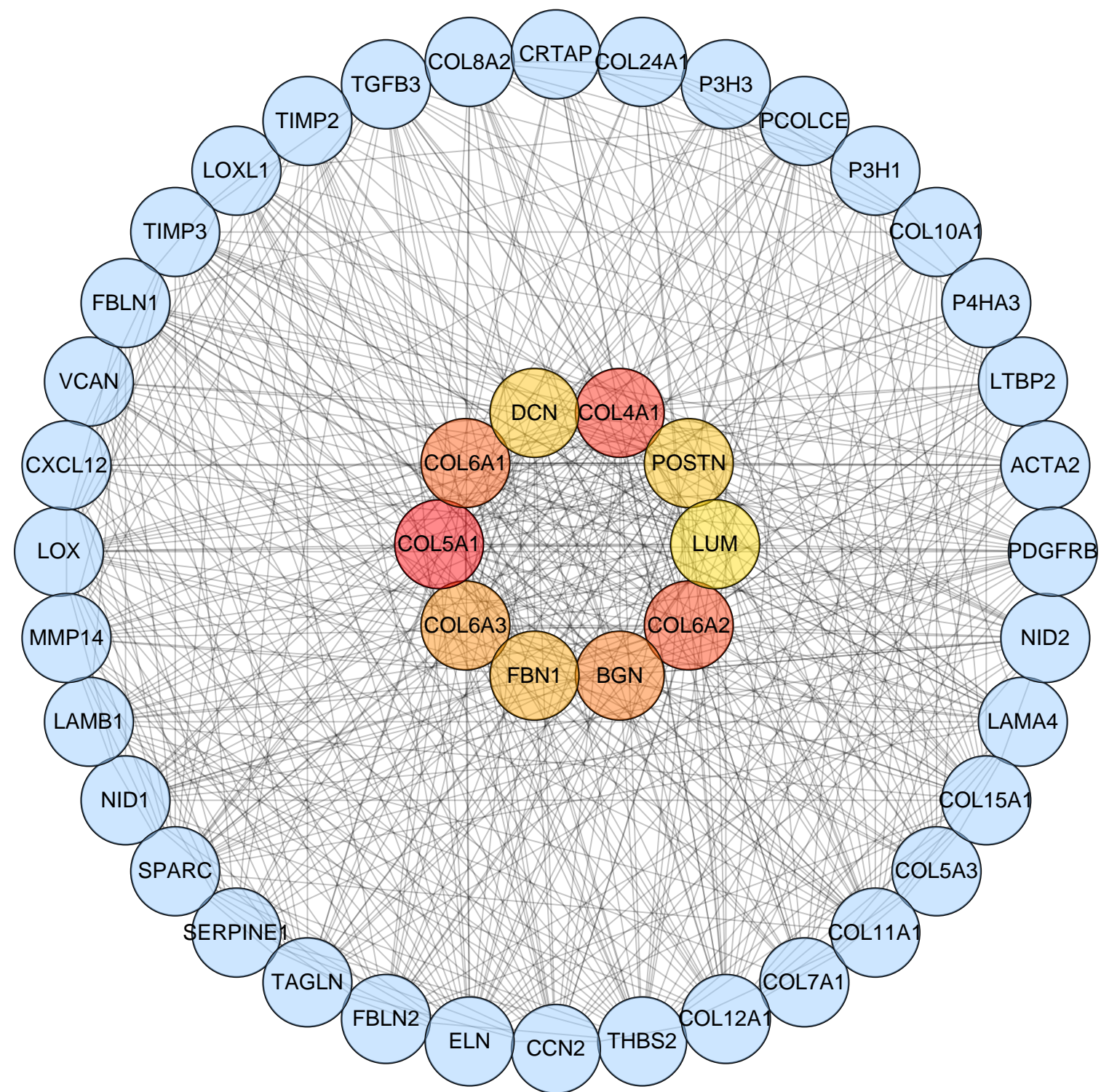

Supplement: Supplemental Information 3 [file peerj-12-18476-s003.zip › 1_Analysis/1_WGCNA/fig1c_magenta_hubgene.pdf]

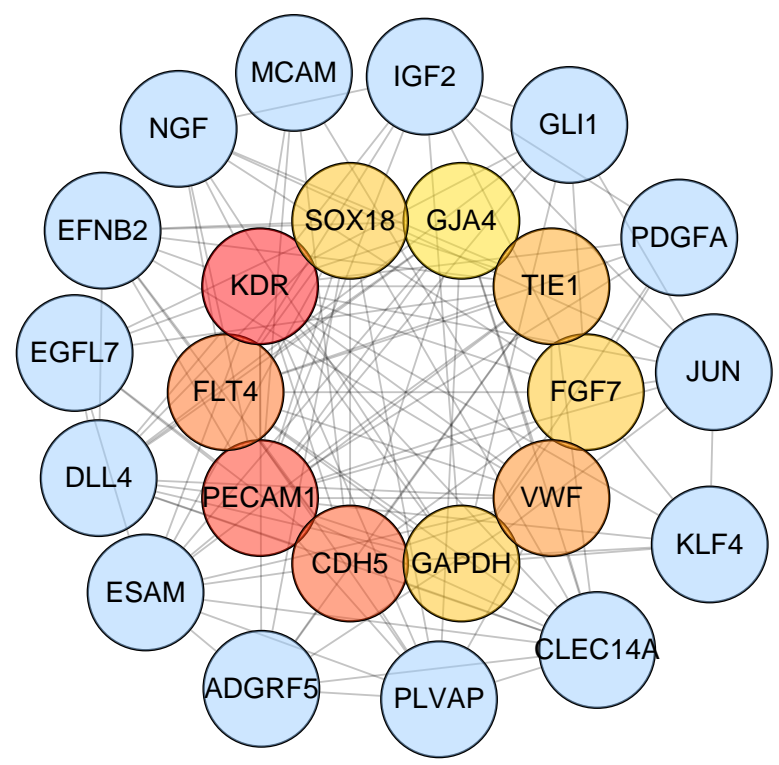

Supplement: Supplemental Information 3 [file peerj-12-18476-s003.zip › 1_Analysis/1_WGCNA/fig1d_pink_hubgene.pdf]

# Biological process of MEmagenta genes

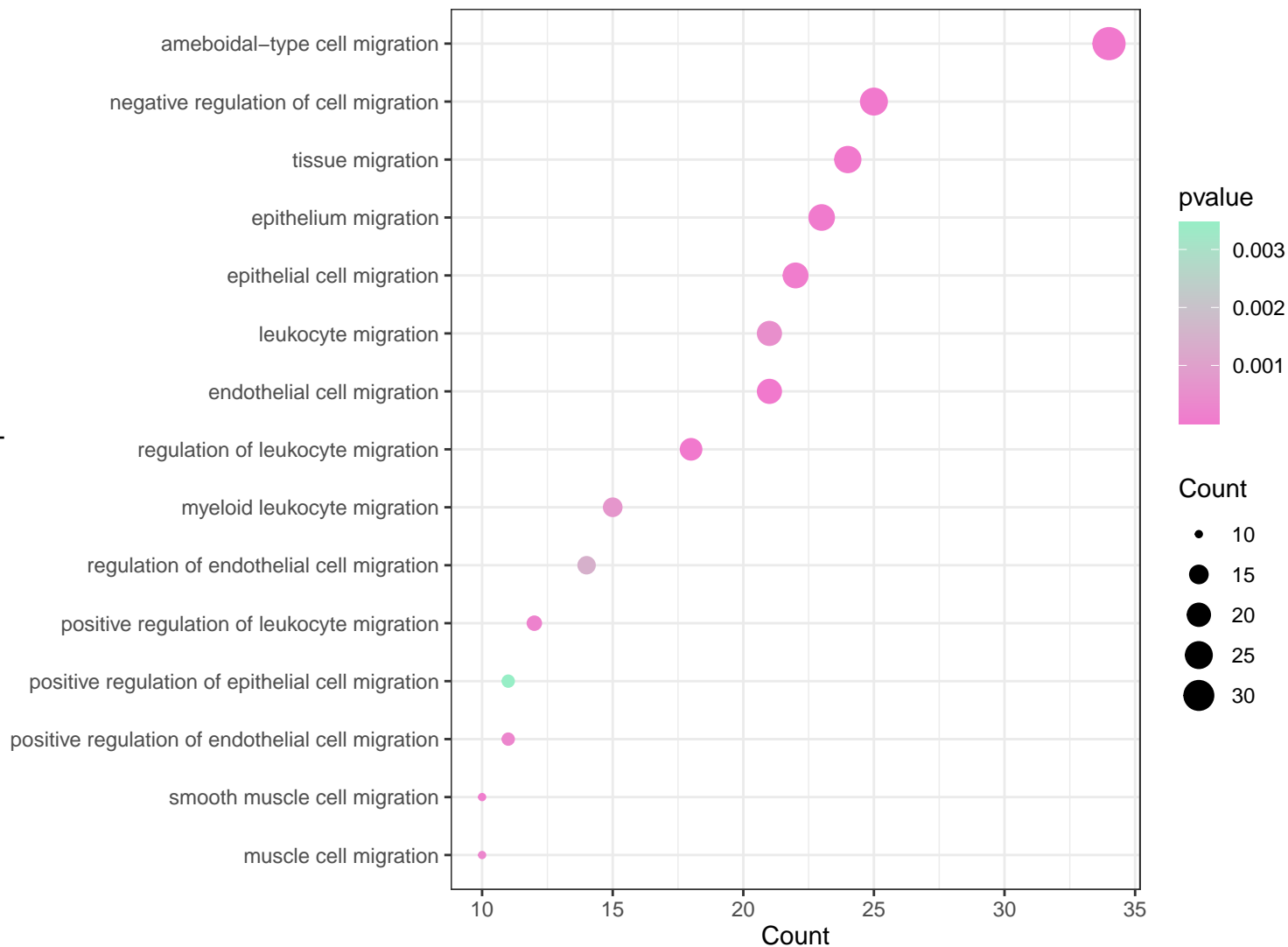

Supplement: Supplemental Information 3 [file peerj-12-18476-s003.zip › 1_Analysis/1_WGCNA/fig1f.pdf]

# Biological process of MEpink genes

Description

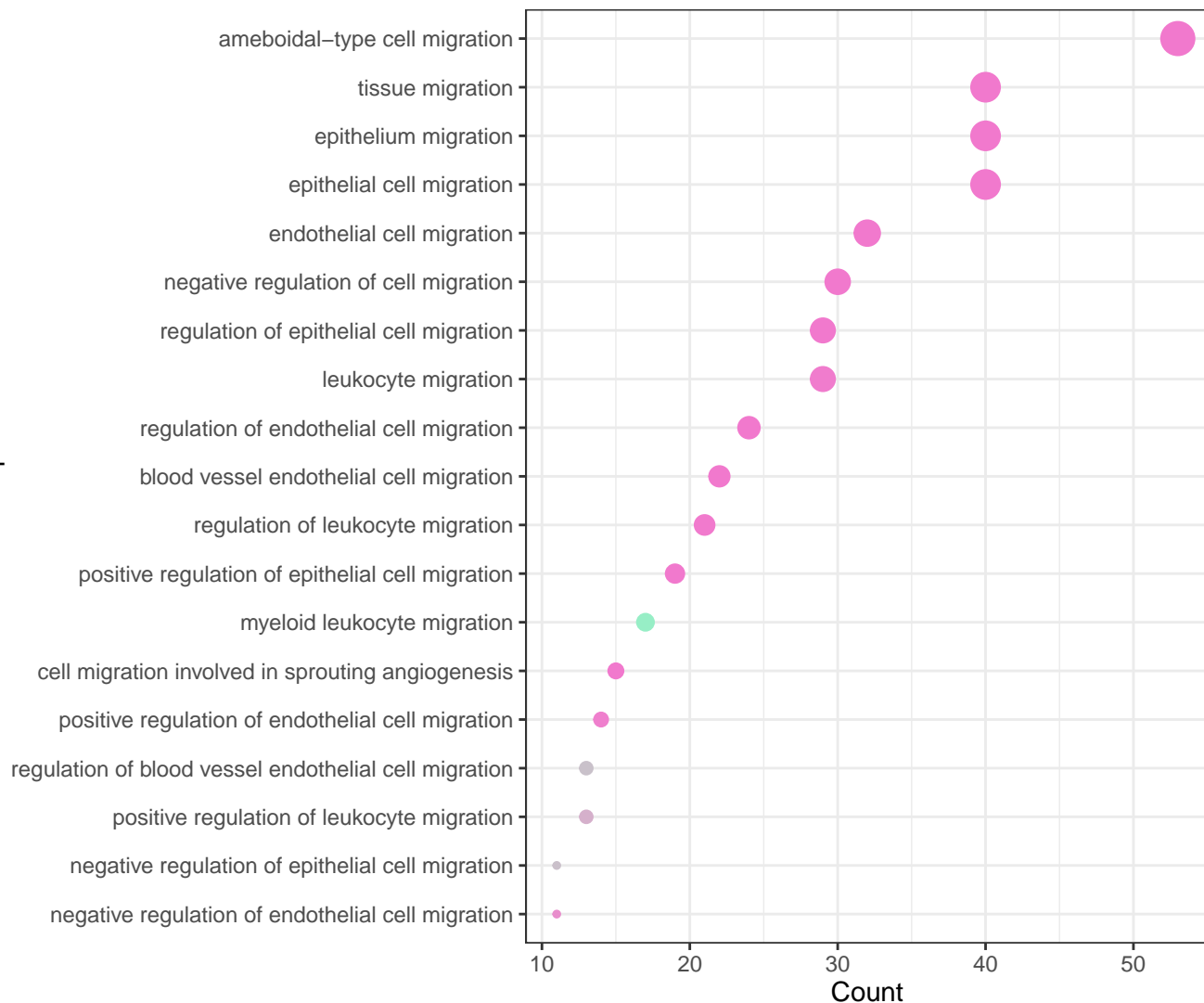

Count

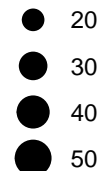

pvalue

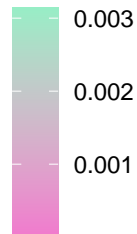

Supplement: Supplemental Information 3 [file peerj-12-18476-s003.zip › 1_Analysis/1_WGCNA/fig1g.pdf]

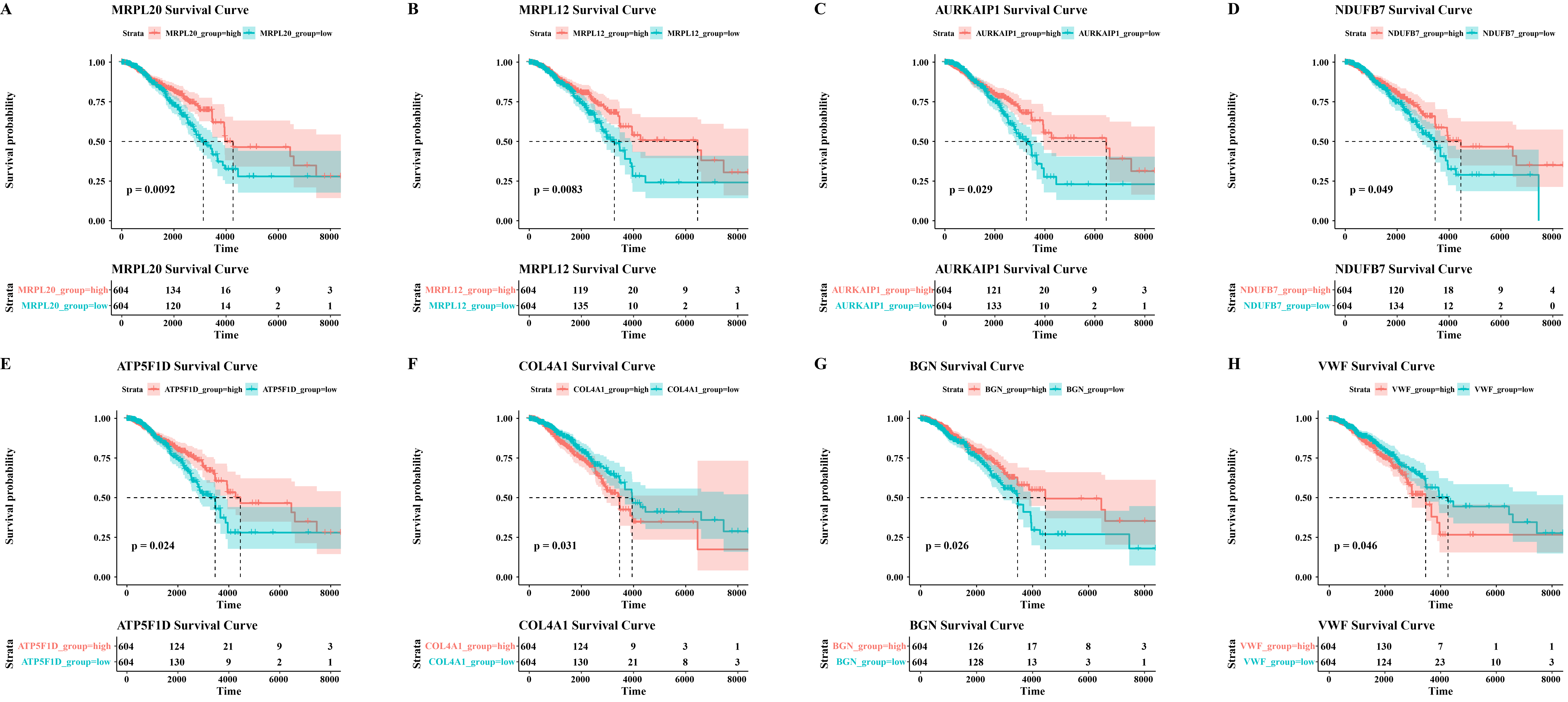

Supplement: Supplemental Information 3 [file peerj-12-18476-s003.zip › 1_Analysis/2_surrivive_analysis/Fig2.png]

**A**

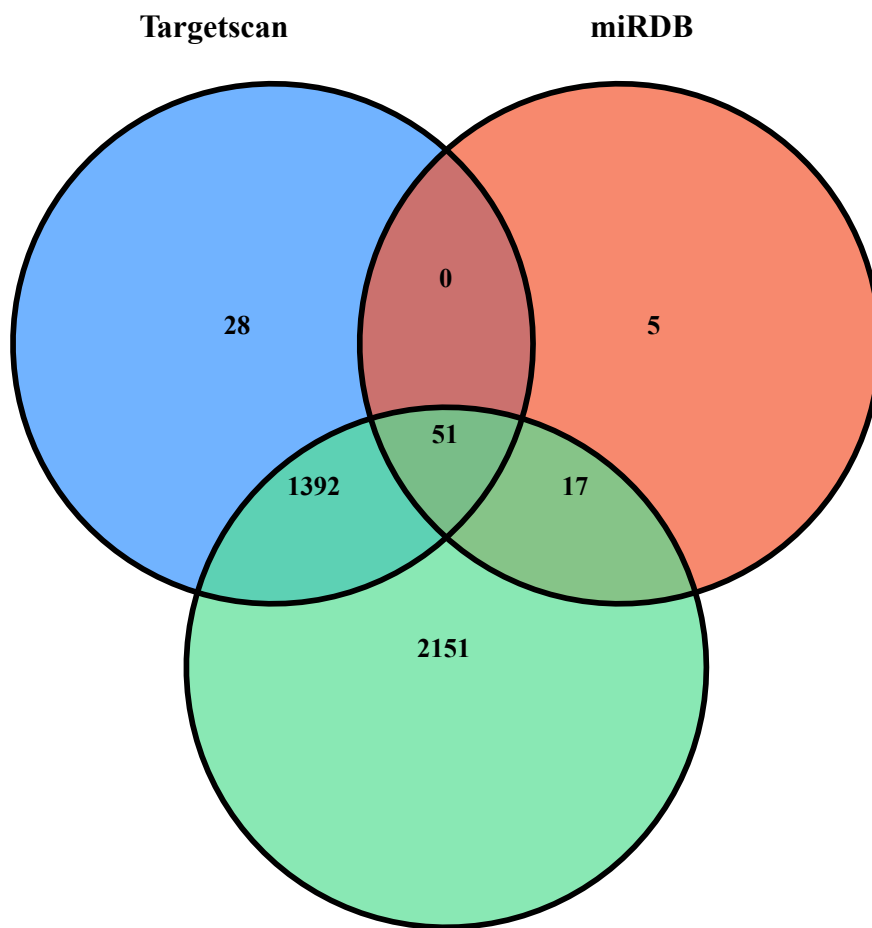

**B**

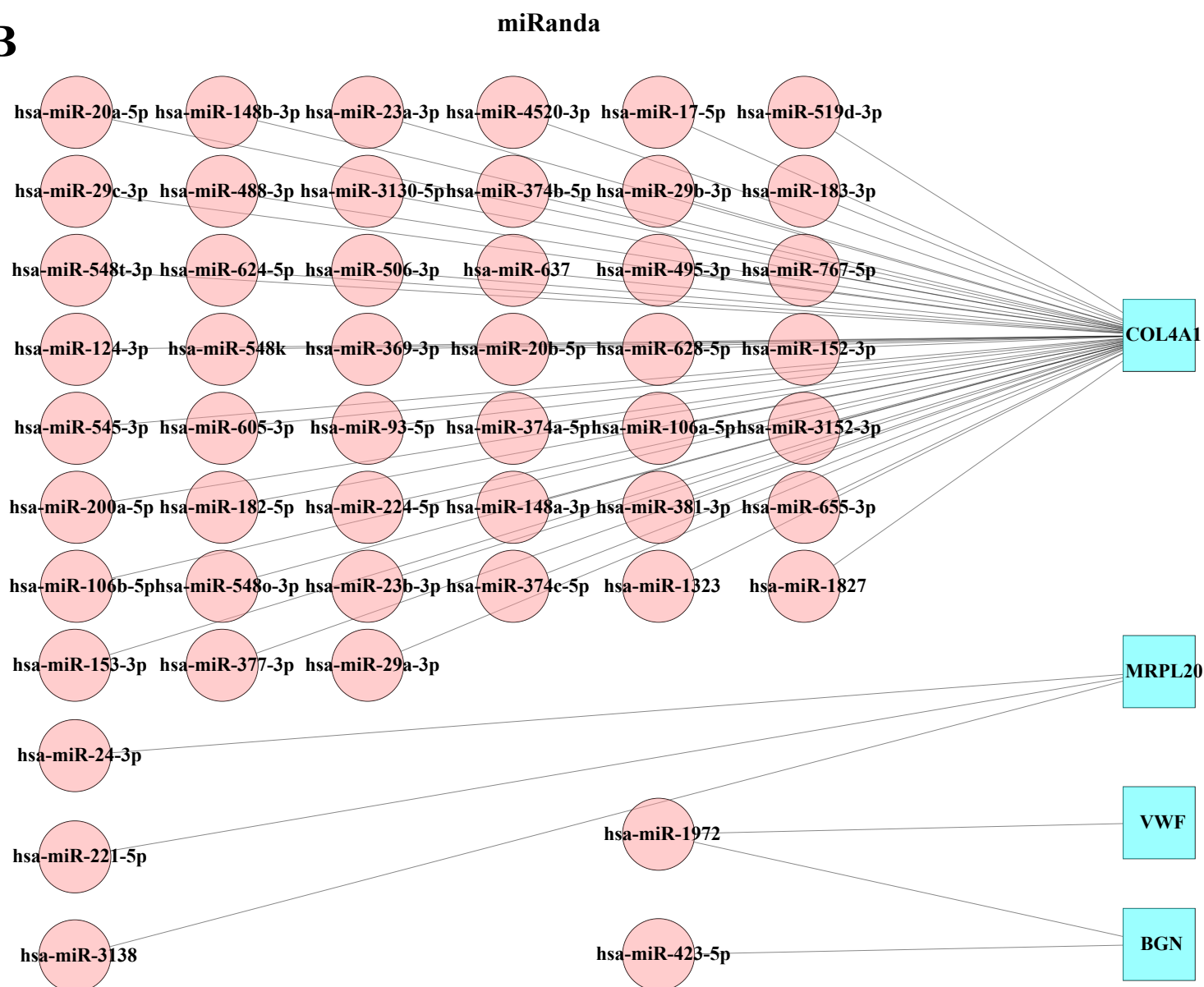

Supplement: Supplemental Information 3 [file peerj-12-18476-s003.zip › 1_Analysis/3_miRNA prediction/Fig3.pdf]

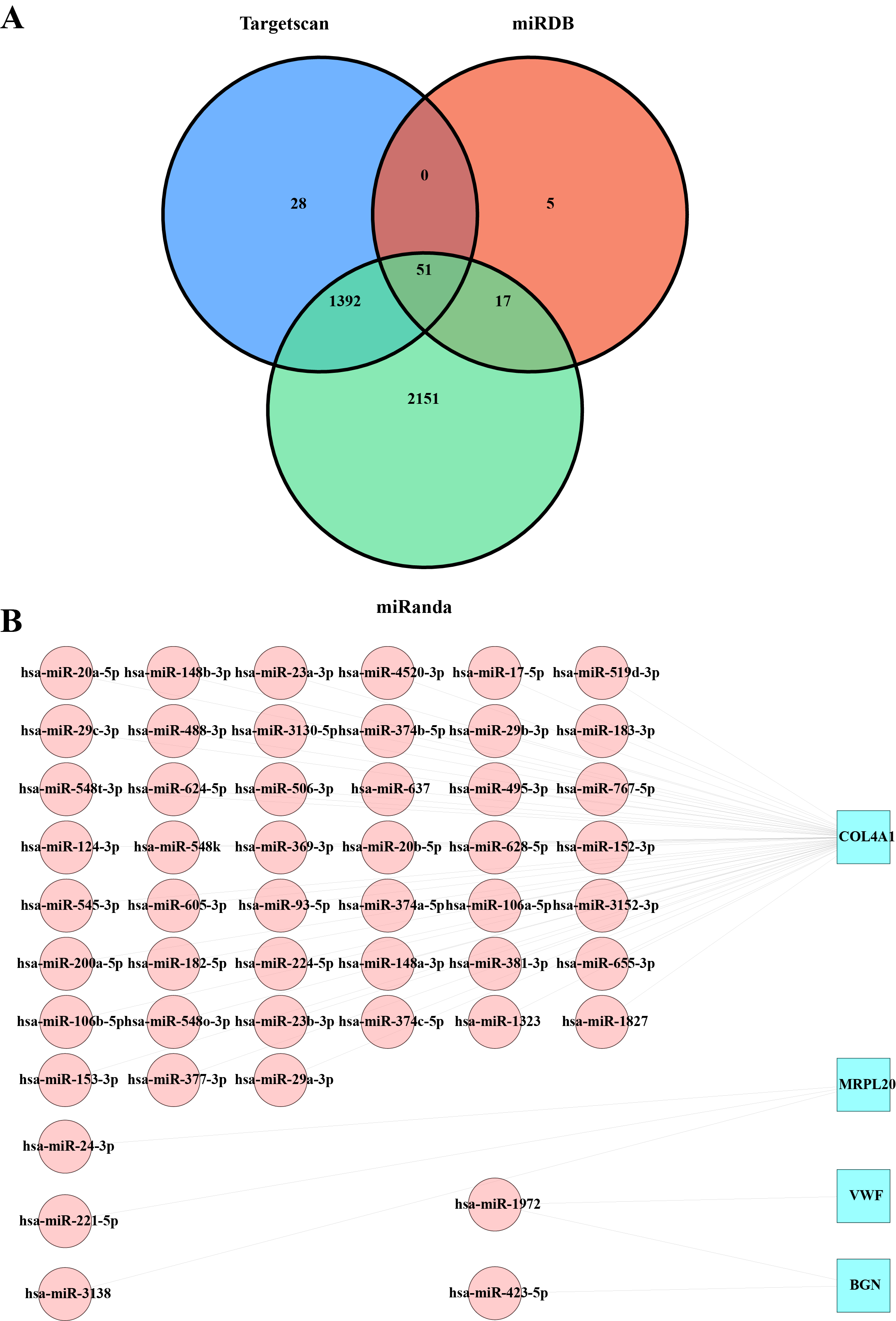

Supplement: Supplemental Information 3 [file peerj-12-18476-s003.zip › 1_Analysis/3_miRNA prediction/Fig3.png]

Targetscan

miRDB

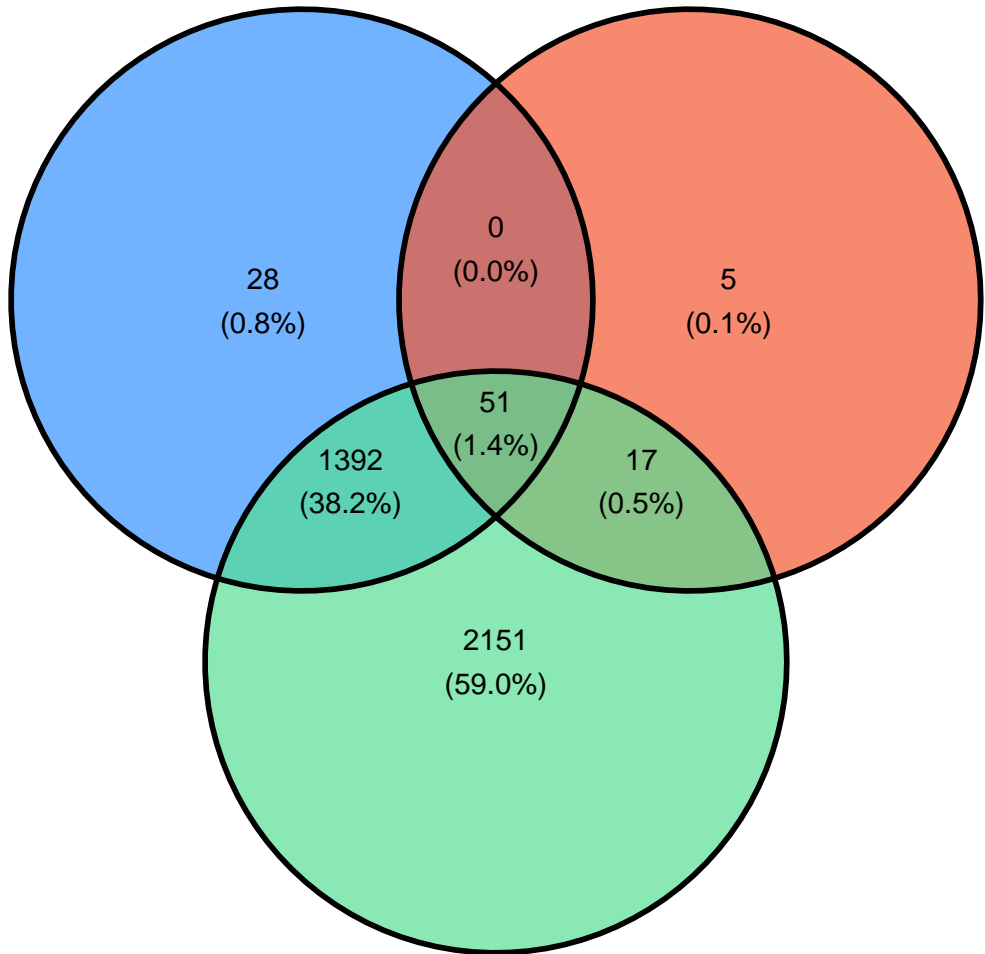

miRanda

Supplement: Supplemental Information 3 [file peerj-12-18476-s003.zip › 1_Analysis/3_miRNA prediction/fig3a.pdf]

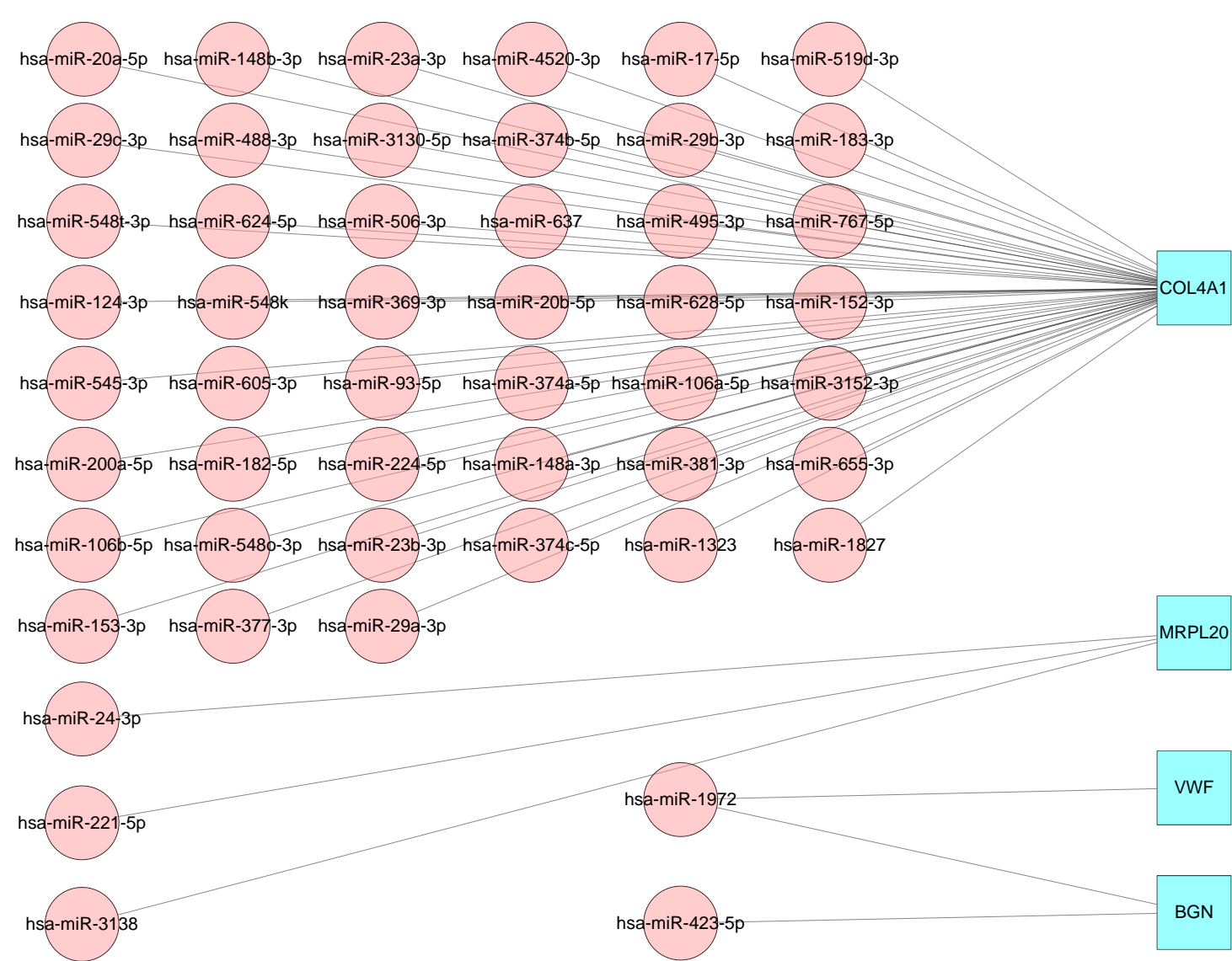

Supplement: Supplemental Information 3 [file peerj-12-18476-s003.zip › 1_Analysis/3_miRNA prediction/fig3b.pdf]

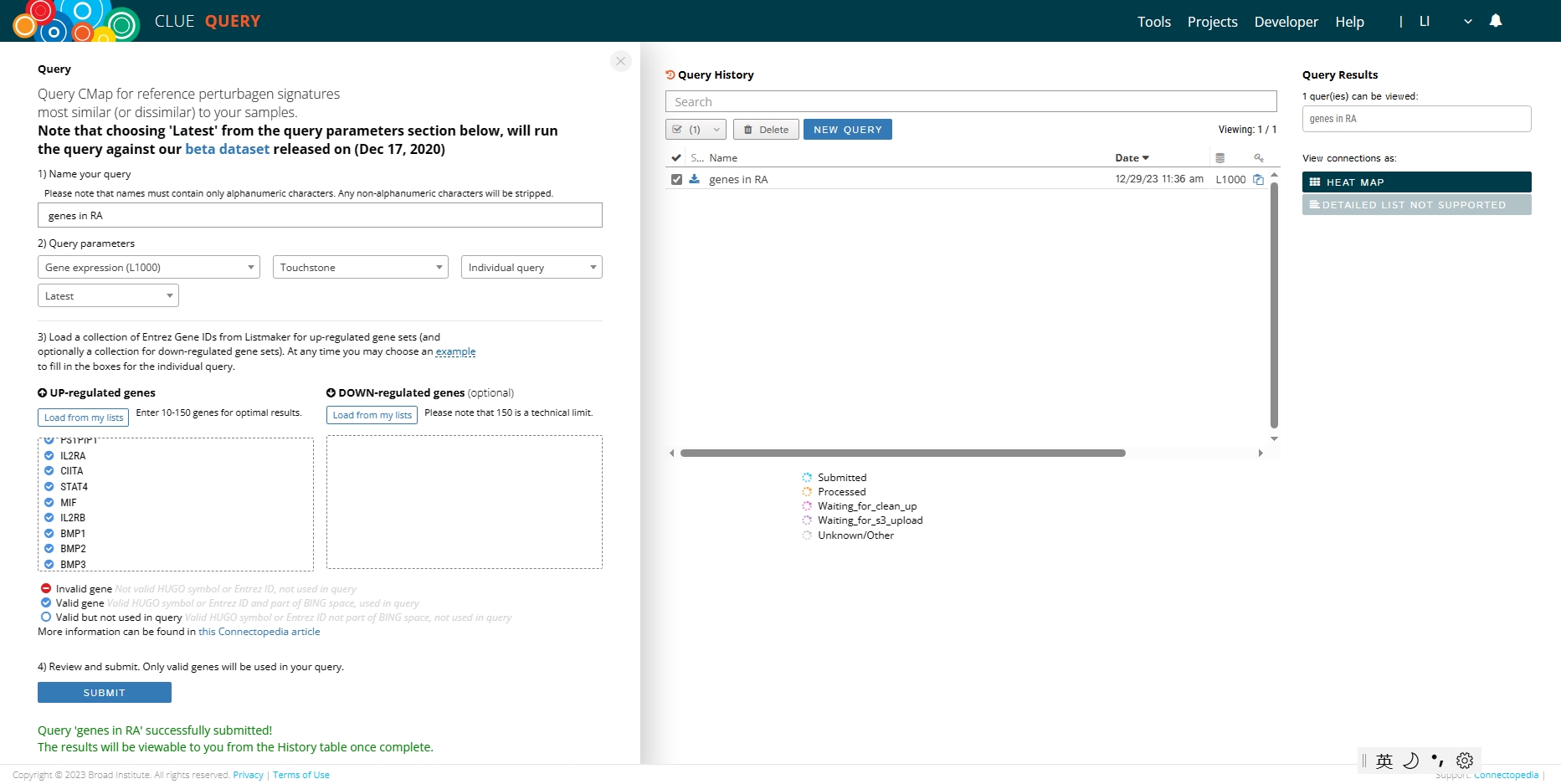

Supplement: Supplemental Information 3 [file peerj-12-18476-s003.zip › Target analysis/cMAP.png]

drug

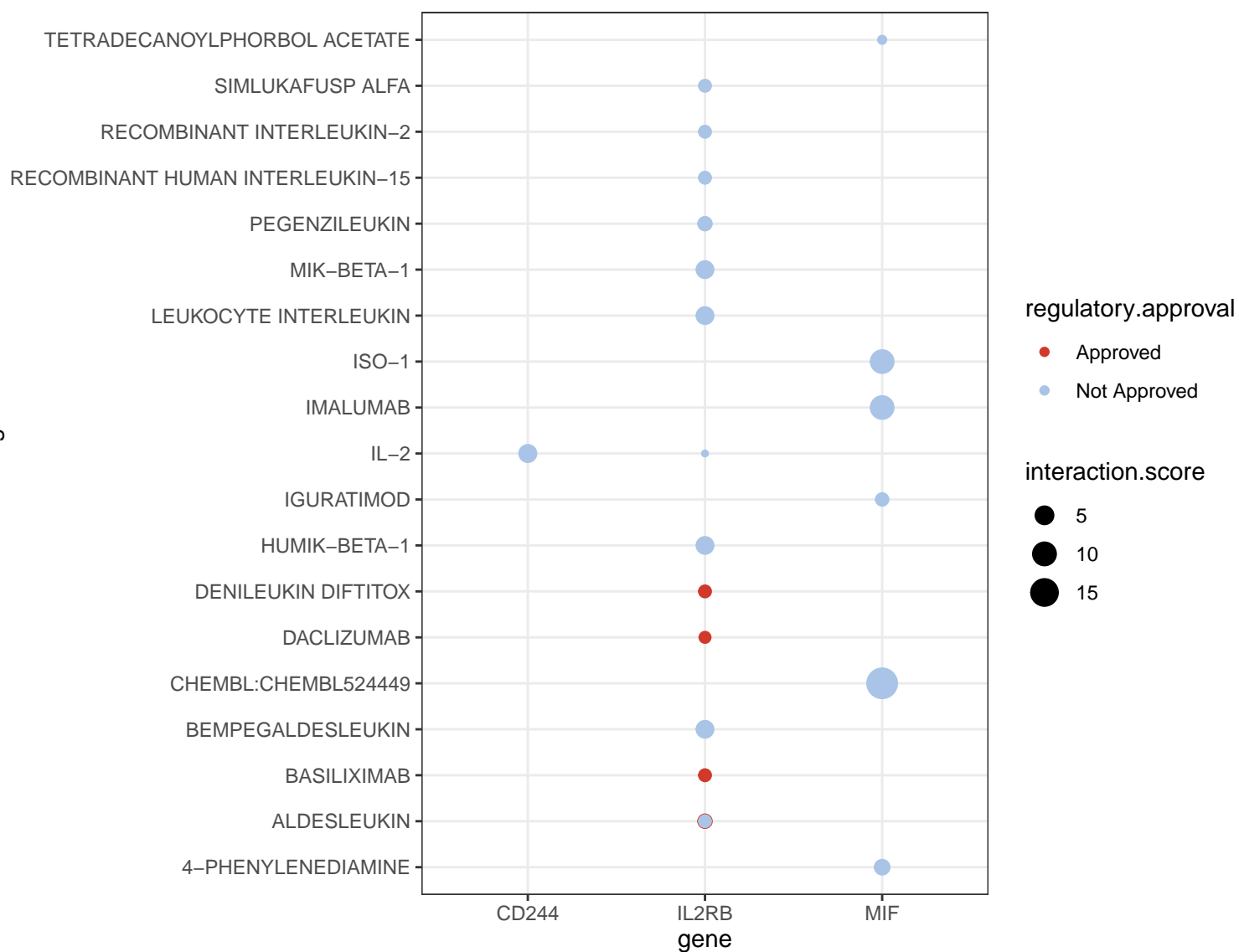

Supplement: Supplemental Information 3 [file peerj-12-18476-s003.zip › Target analysis/fig1a.pdf]

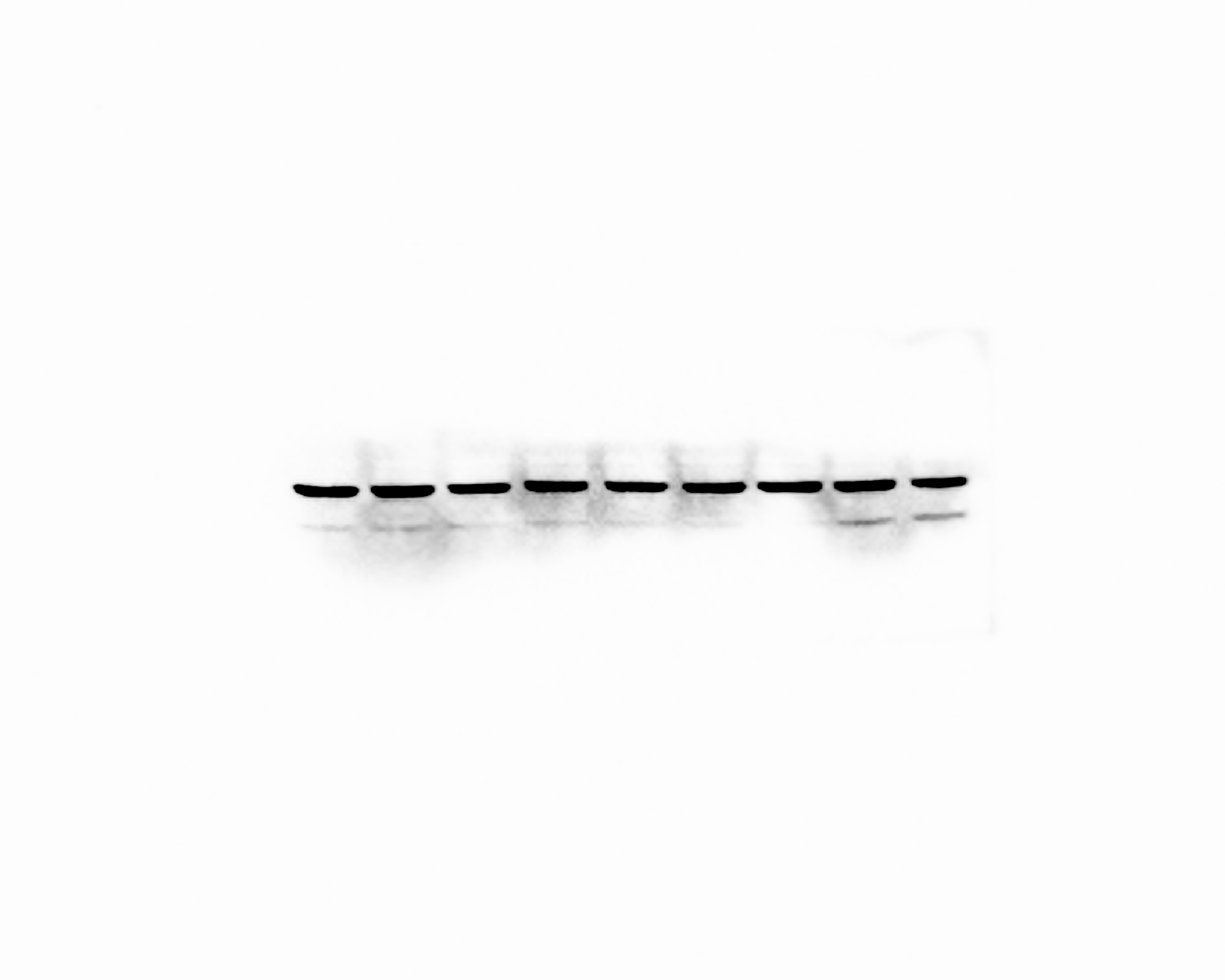

Supplement: Supplemental Information 5 [file peerj-12-18476-s005.zip › Figure 6 raw data/vgd 2(Chemiluminescence).tif]

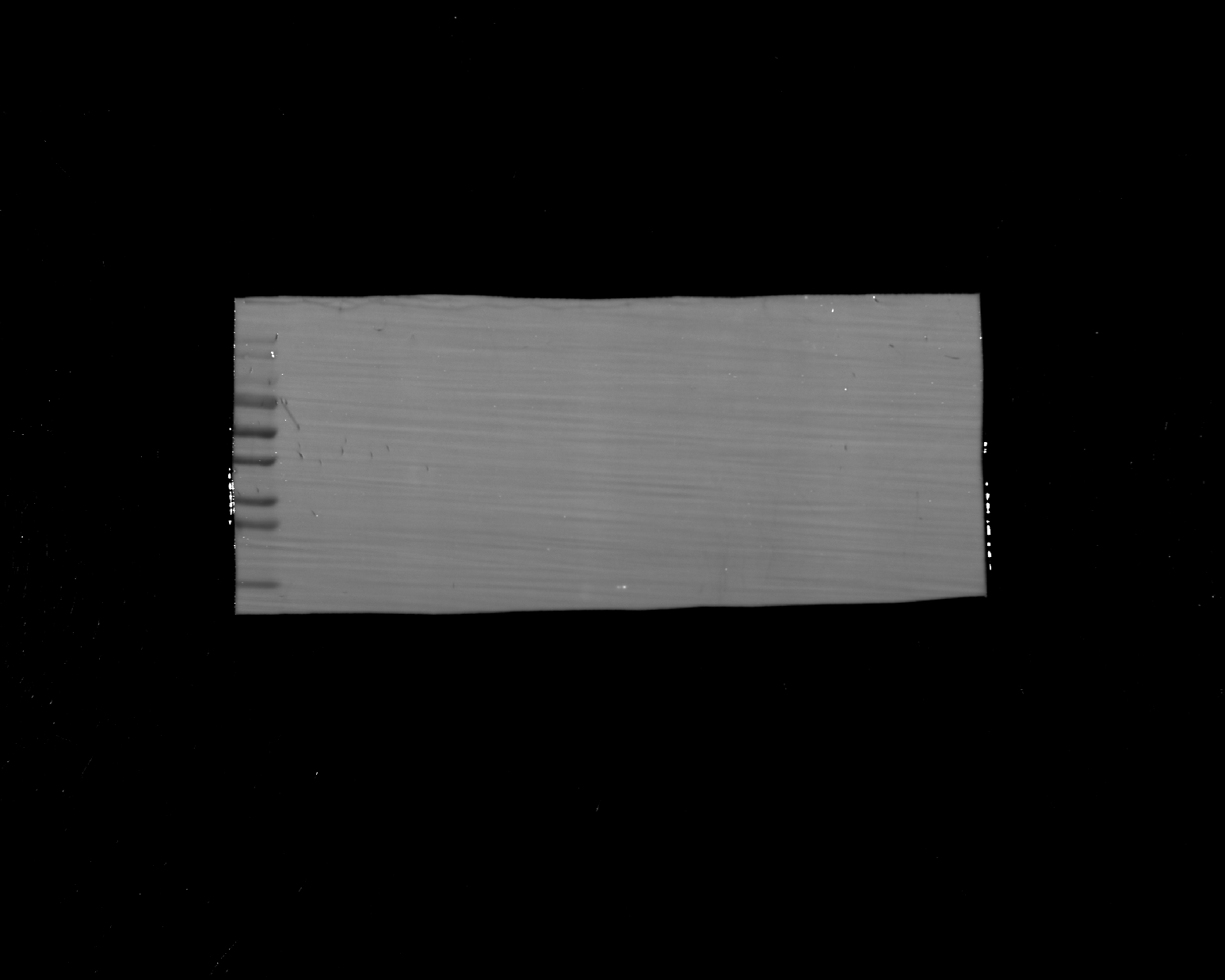

Supplement: Supplemental Information 5 [file peerj-12-18476-s005.zip › Figure 6 raw data/vgd 2(Colorimetric).tif]

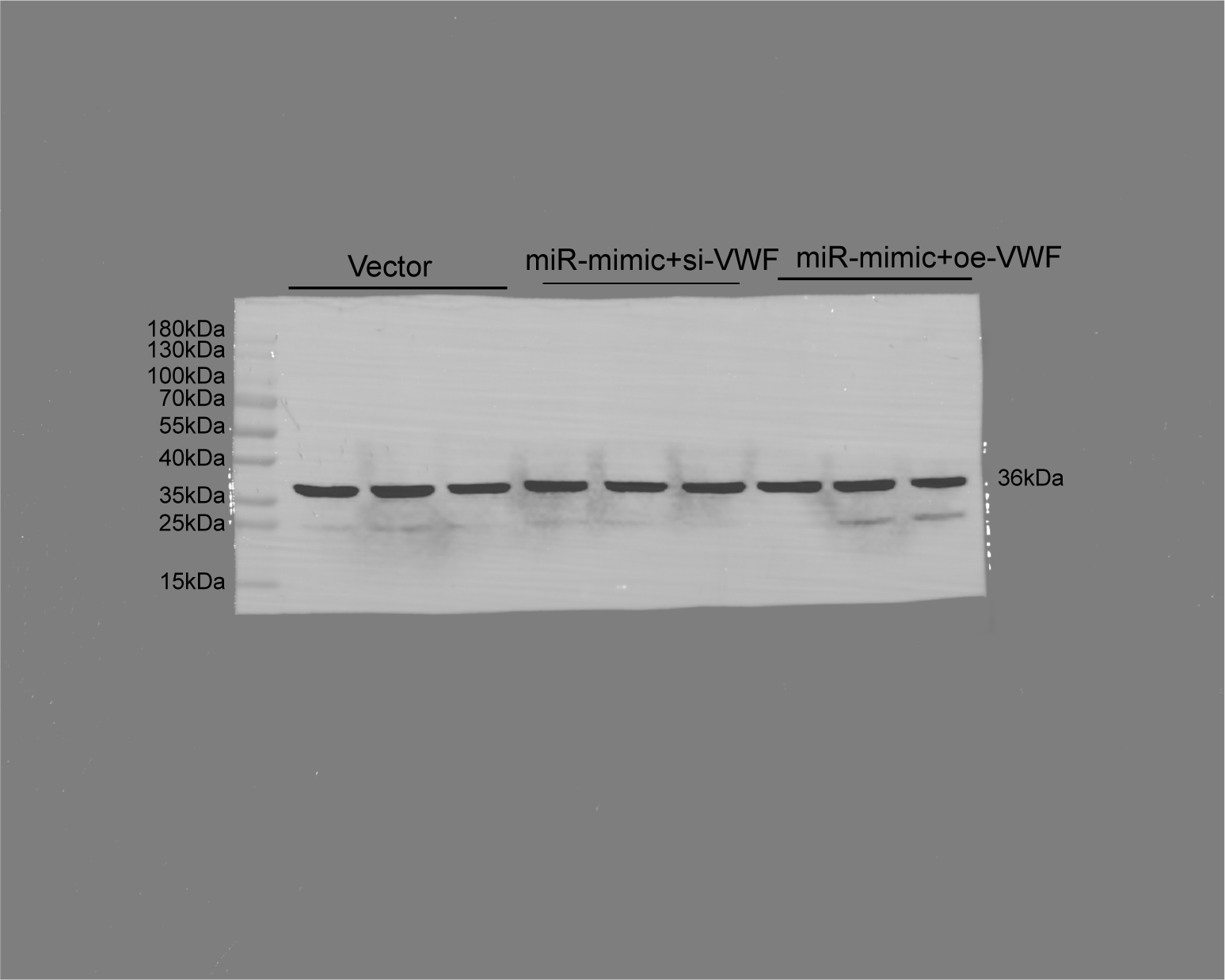

Supplement: Supplemental Information 5 [file peerj-12-18476-s005.zip › Figure 6 raw data/vgd 2(Composite)-01.tif]

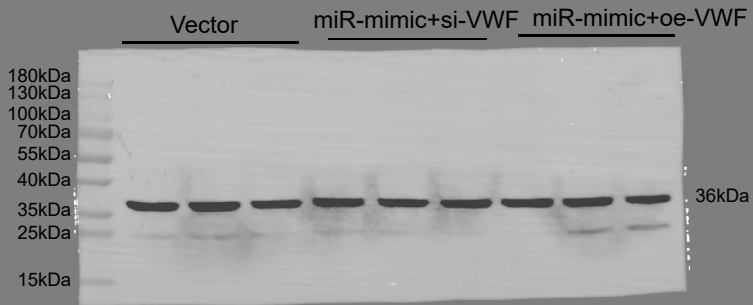

Supplement: Supplemental Information 5 [file peerj-12-18476-s005.zip › Figure 6 raw data/vgd 2(Composite).pdf]

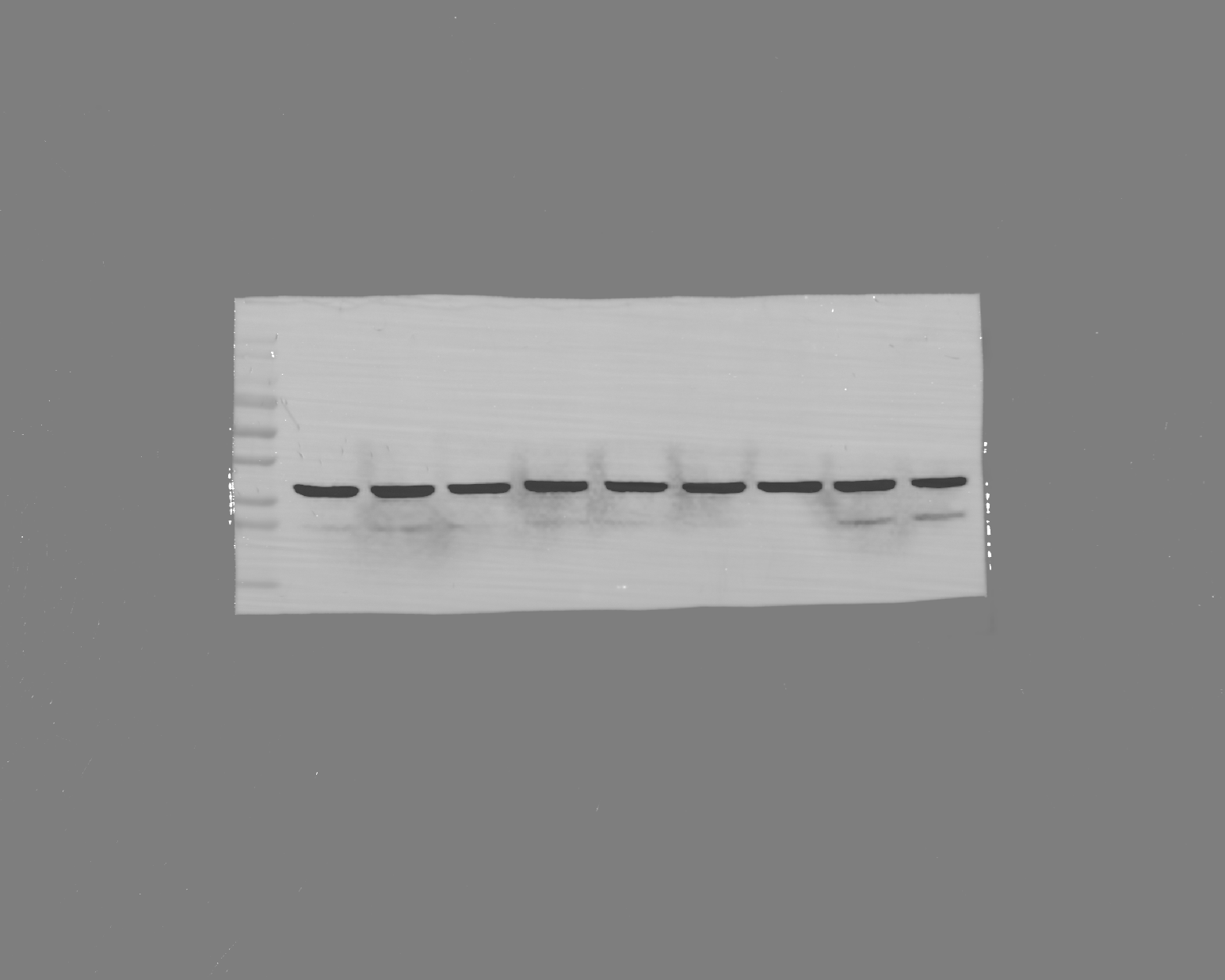

Supplement: Supplemental Information 5 [file peerj-12-18476-s005.zip › Figure 6 raw data/vgd 2(Composite).tif]

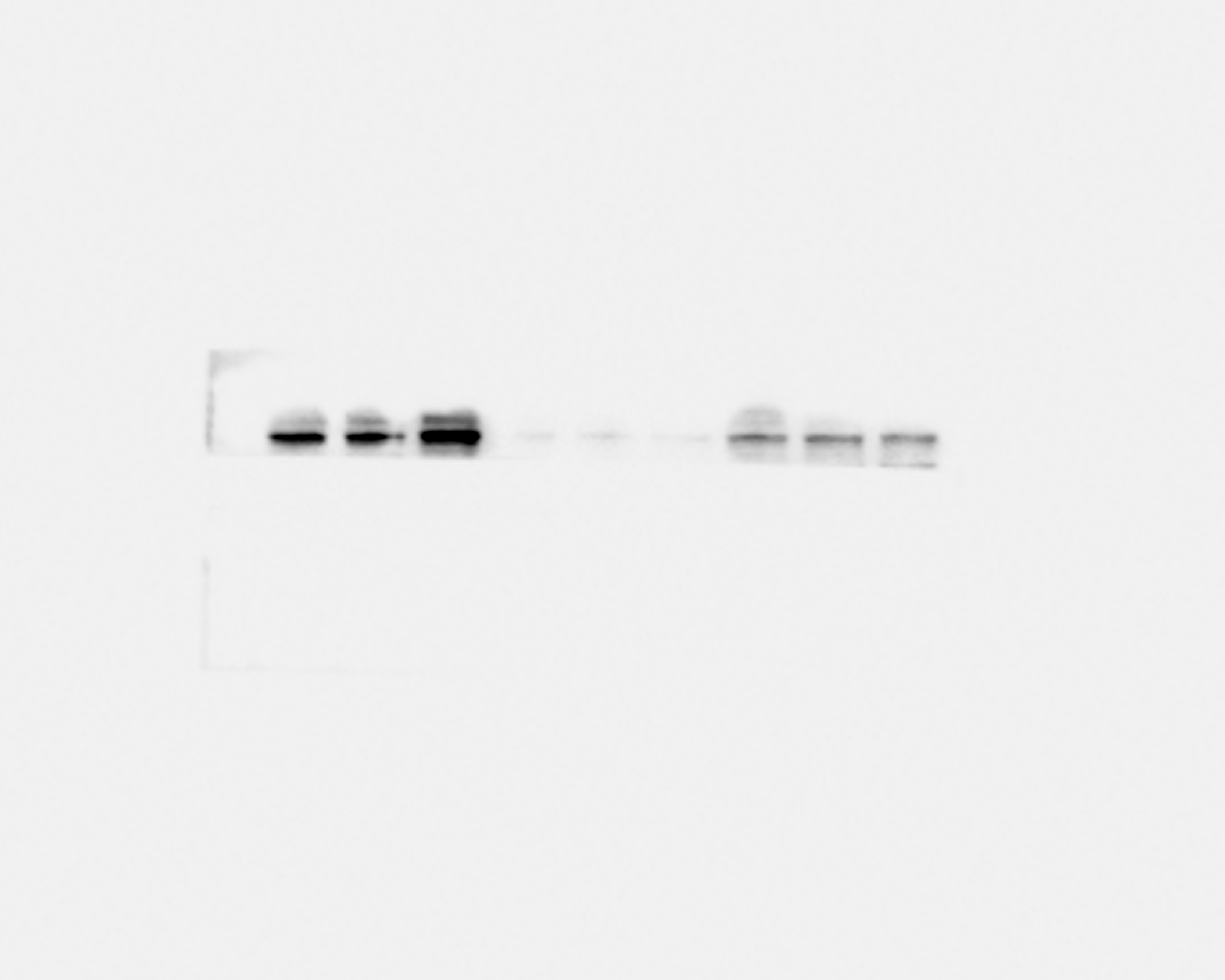

Supplement: Supplemental Information 5 [file peerj-12-18476-s005.zip › Figure 6 raw data/vwf 2(Chemiluminescence).tif]

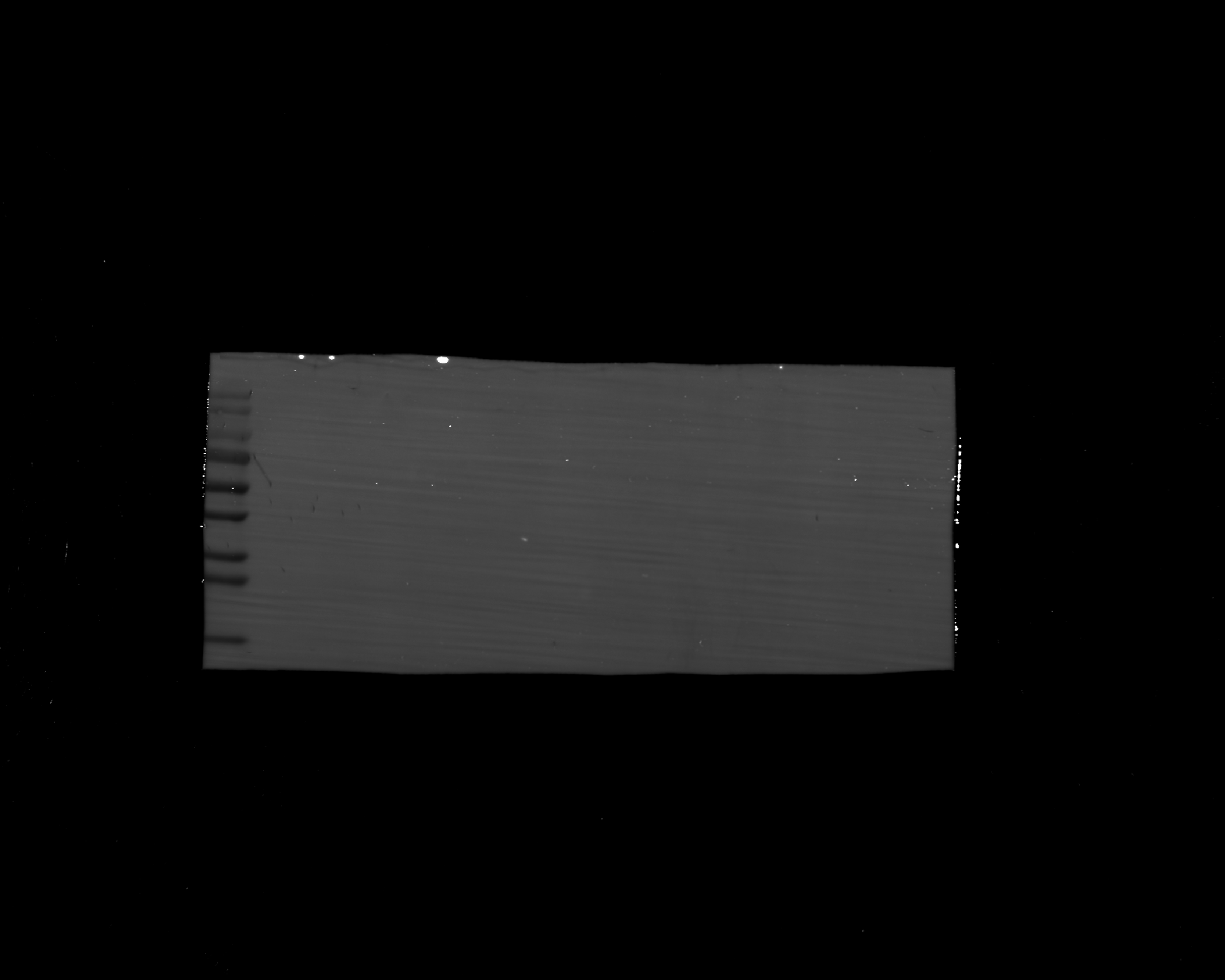

Supplement: Supplemental Information 5 [file peerj-12-18476-s005.zip › Figure 6 raw data/vwf 2(Colorimetric).tif]

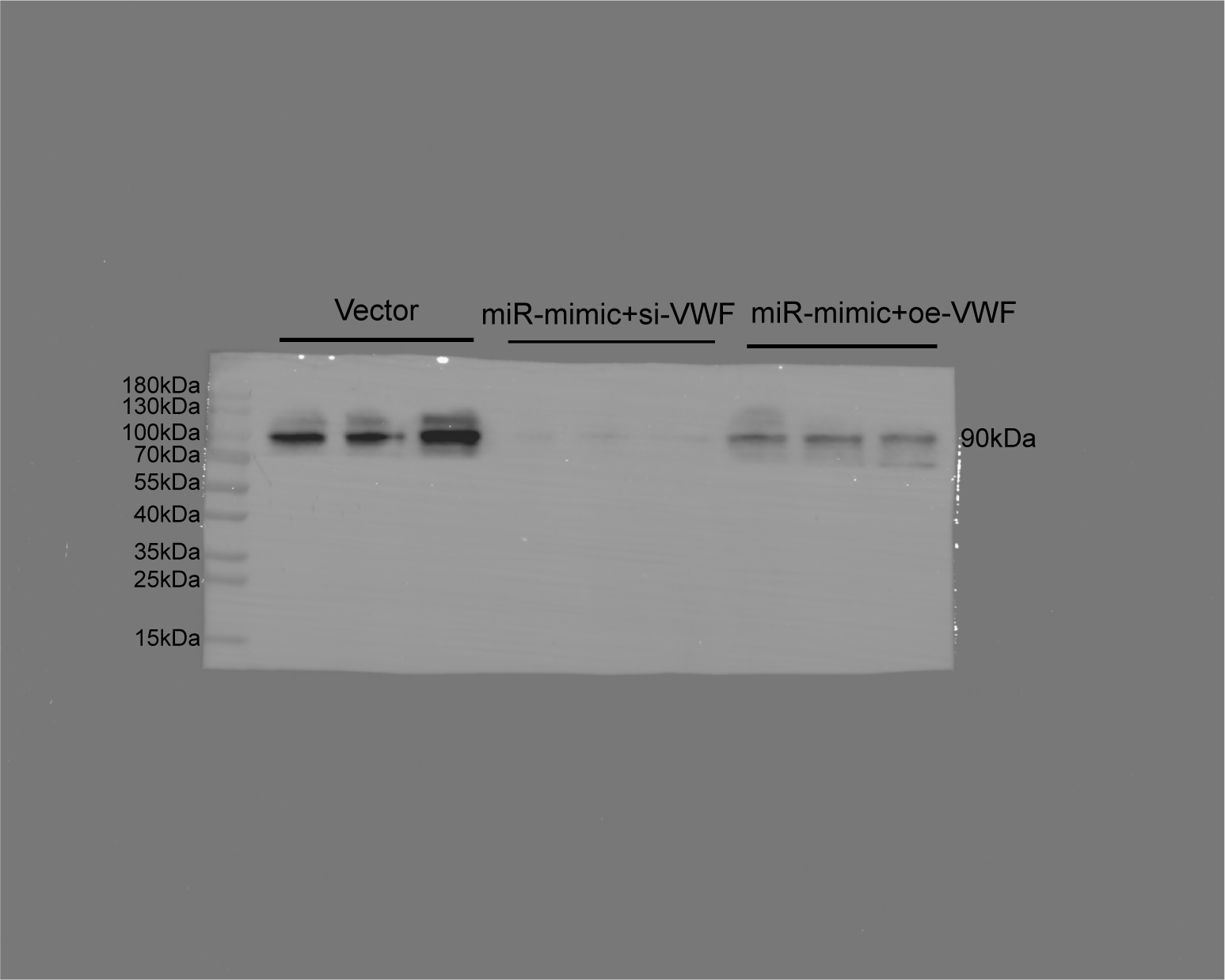

Supplement: Supplemental Information 5 [file peerj-12-18476-s005.zip › Figure 6 raw data/vwf 2(Composite)-01.tif]

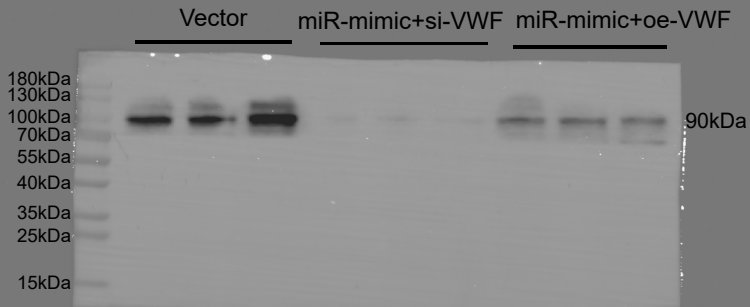

Supplement: Supplemental Information 5 [file peerj-12-18476-s005.zip › Figure 6 raw data/vwf 2(Composite).pdf]

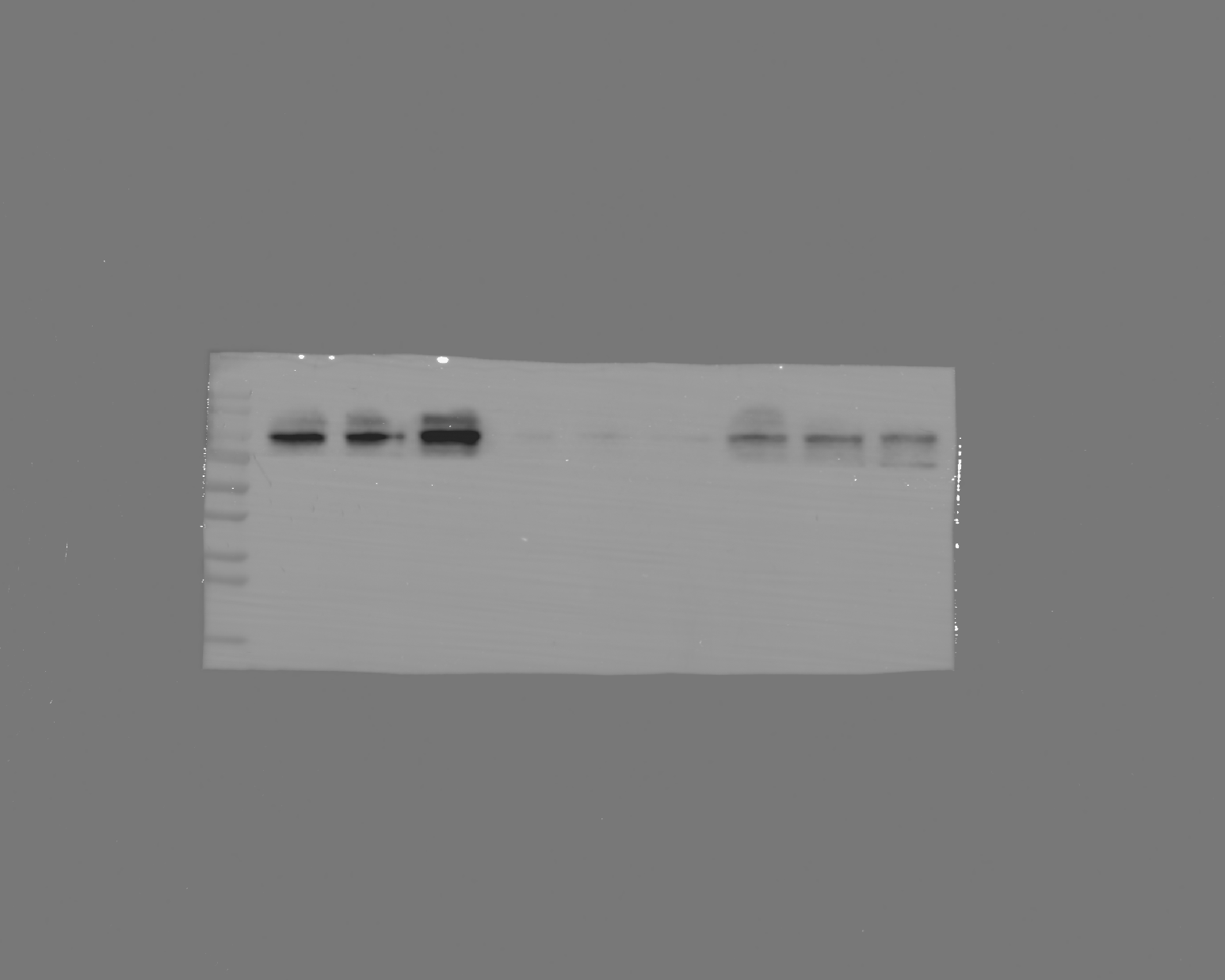

Supplement: Supplemental Information 5 [file peerj-12-18476-s005.zip › Figure 6 raw data/vwf 2(Composite).tif]

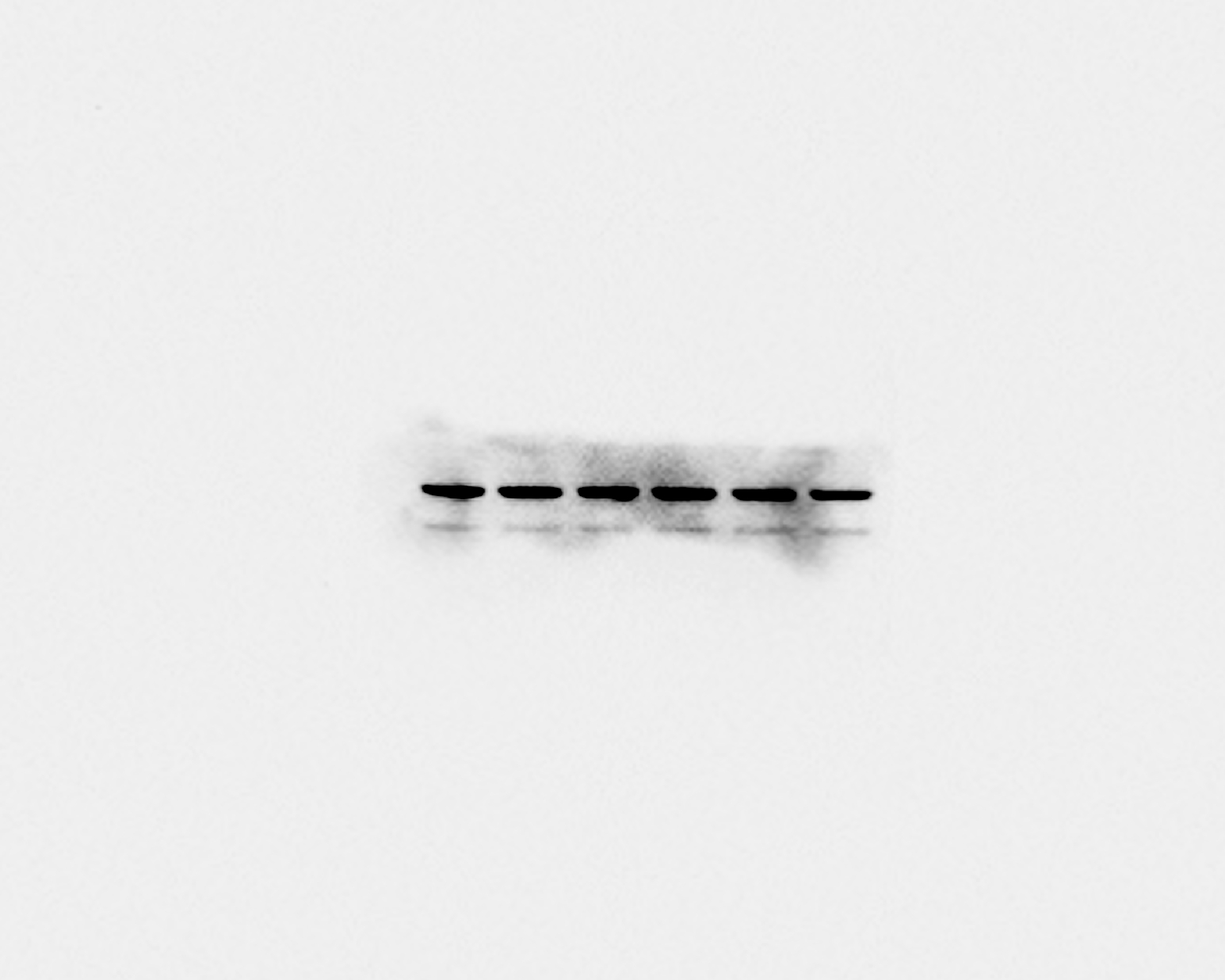

Supplement: Supplemental Information 6 [file peerj-12-18476-s006.zip › wb/repeat 1/gapdh 1(Chemiluminescence).tif]

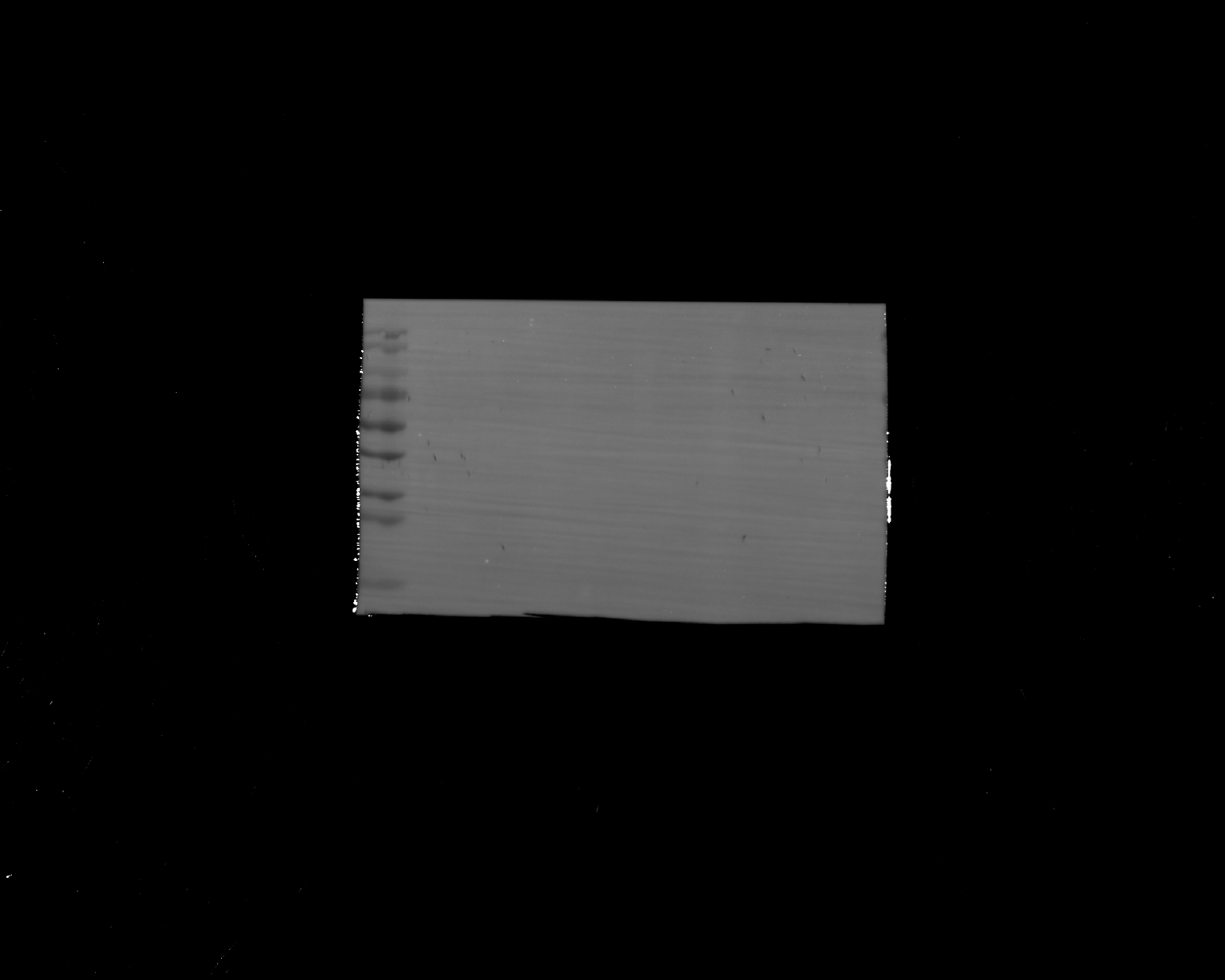

Supplement: Supplemental Information 6 [file peerj-12-18476-s006.zip › wb/repeat 1/gapdh 1(Colorimetric).tif]

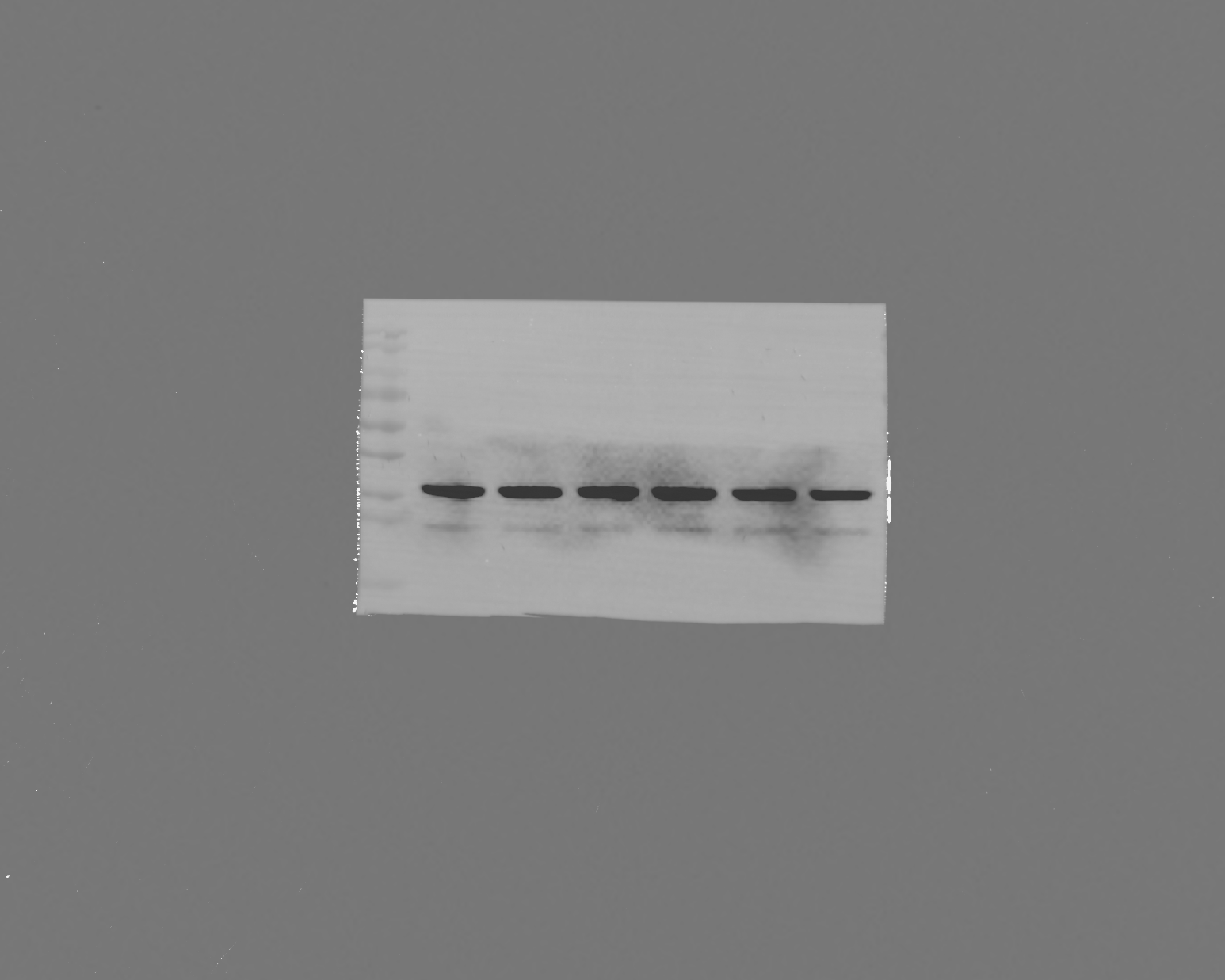

Supplement: Supplemental Information 6 [file peerj-12-18476-s006.zip › wb/repeat 1/gapdh 1(Composite).tif]

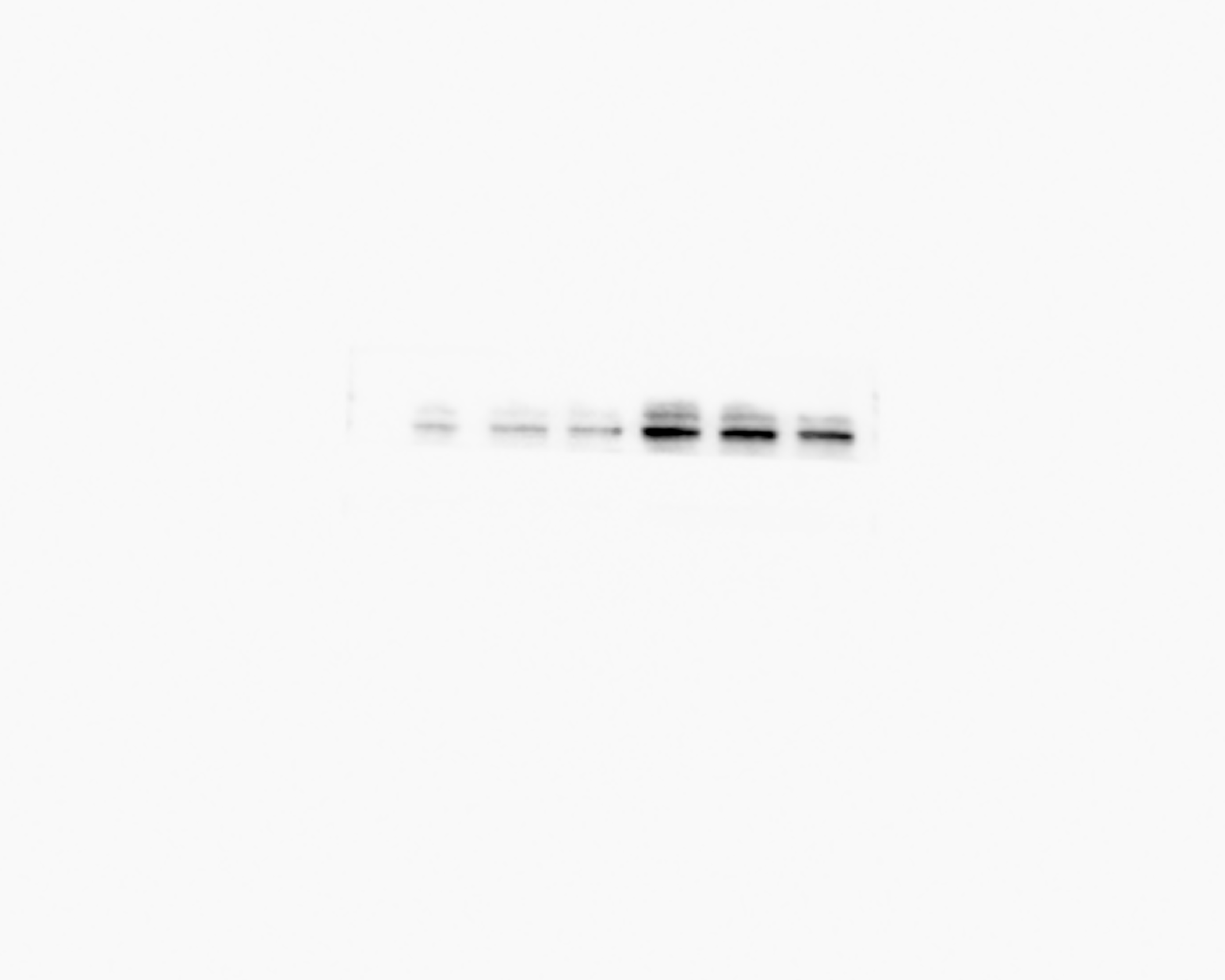

Supplement: Supplemental Information 6 [file peerj-12-18476-s006.zip › wb/repeat 1/vwf 1(Chemiluminescence).tif]

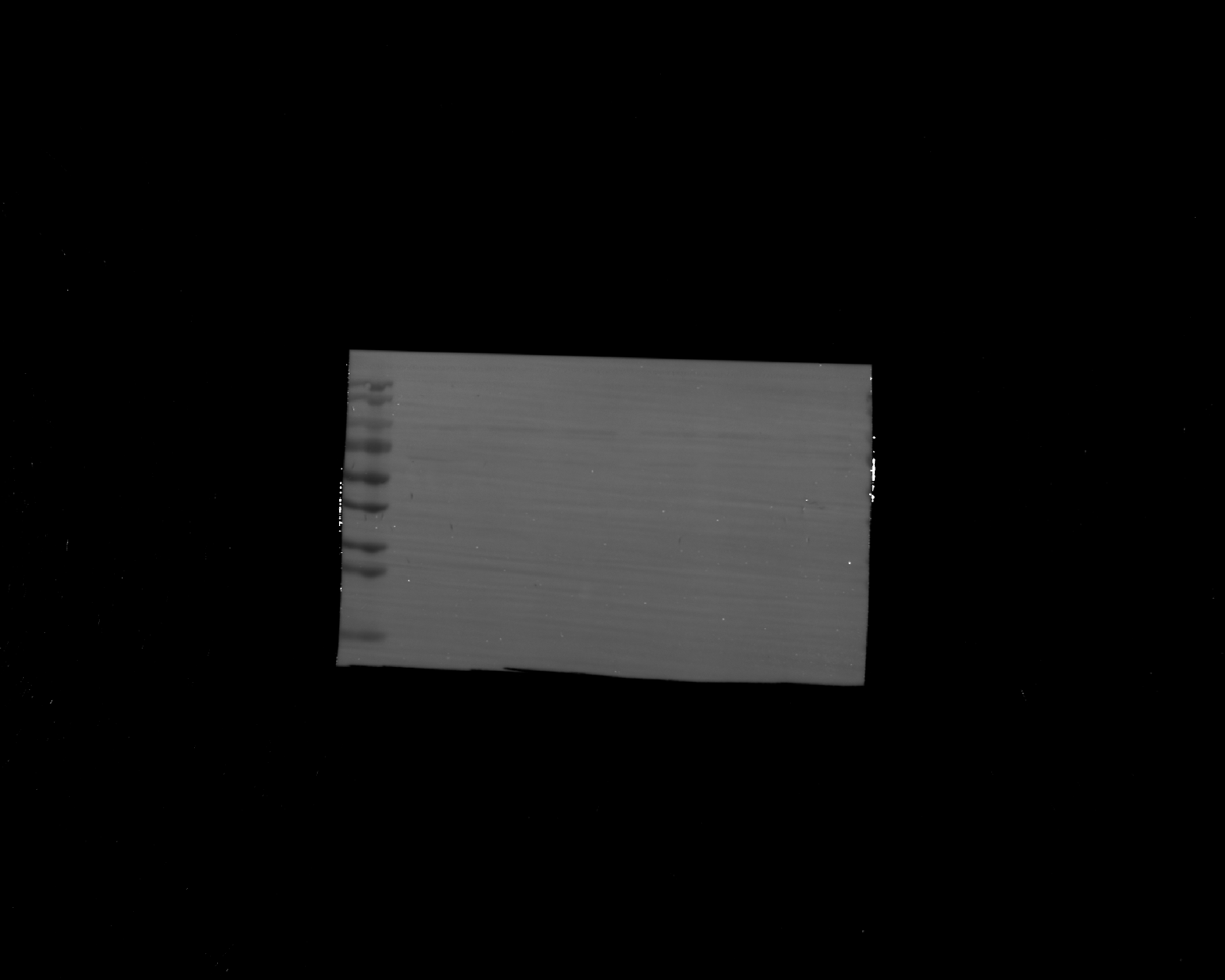

Supplement: Supplemental Information 6 [file peerj-12-18476-s006.zip › wb/repeat 1/vwf 1(Colorimetric).tif]

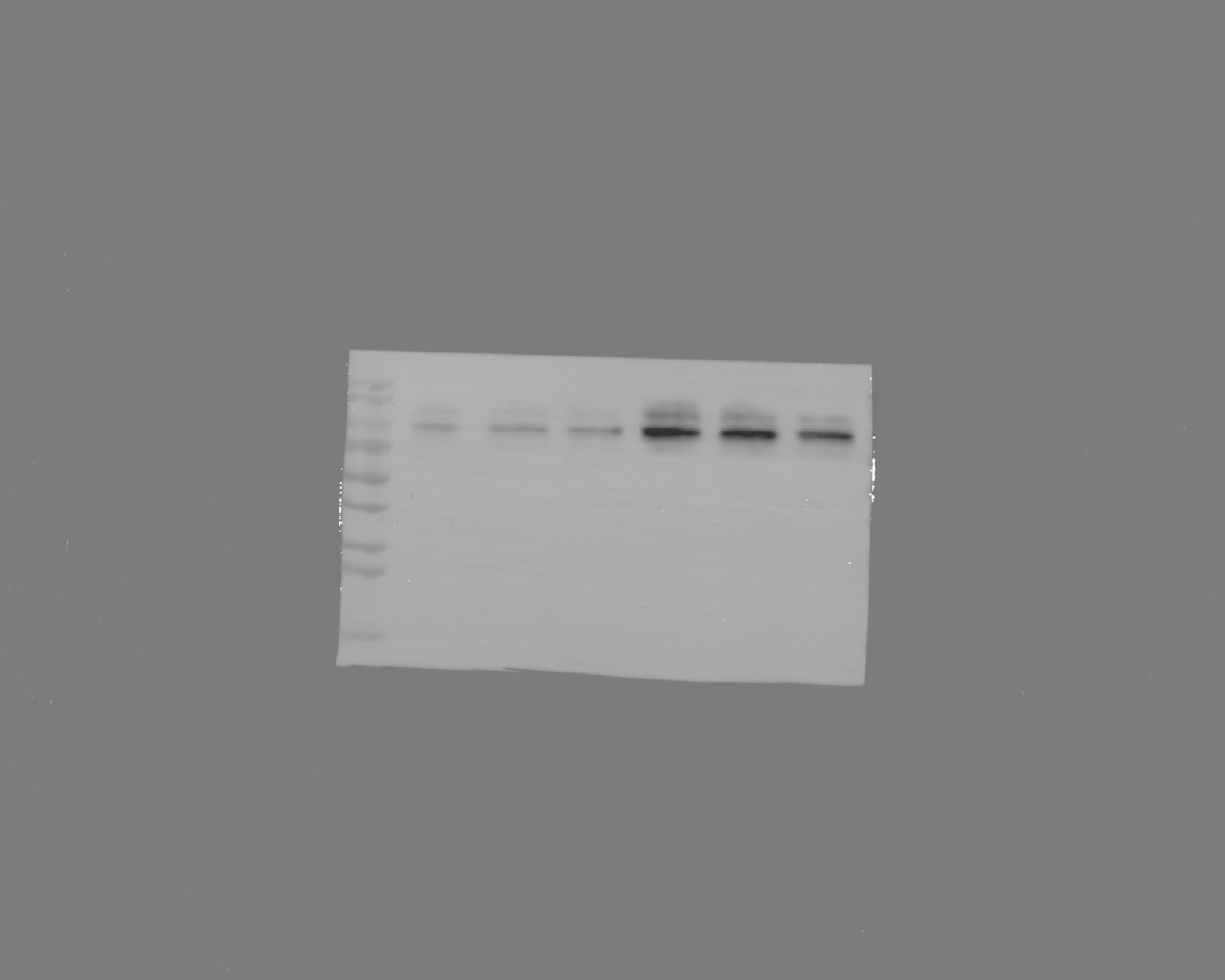

Supplement: Supplemental Information 6 [file peerj-12-18476-s006.zip › wb/repeat 1/vwf 1(Composite).tif]

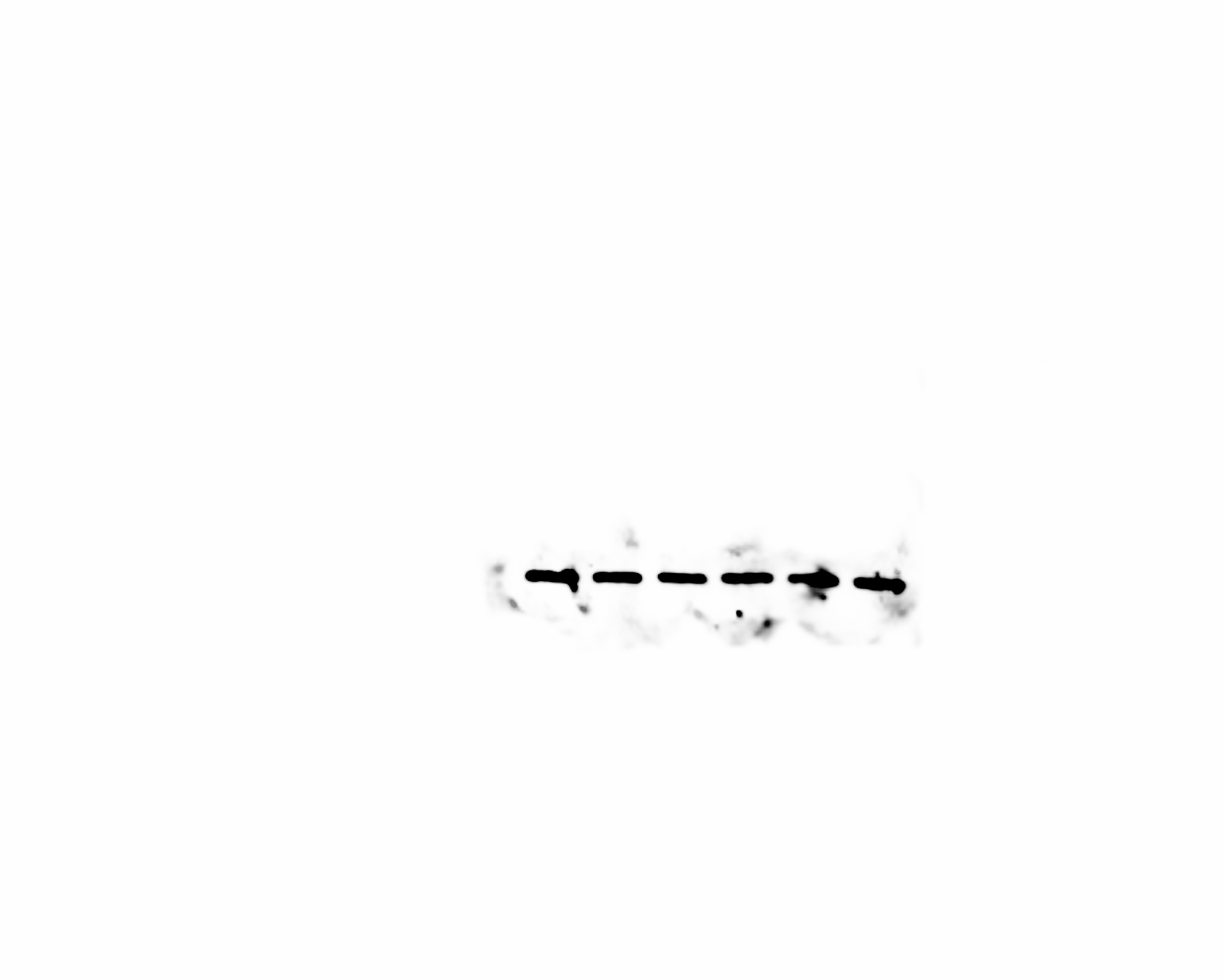

Supplement: Supplemental Information 6 [file peerj-12-18476-s006.zip › wb/repeat 2/gapdh 2(Chemiluminescence).tif]

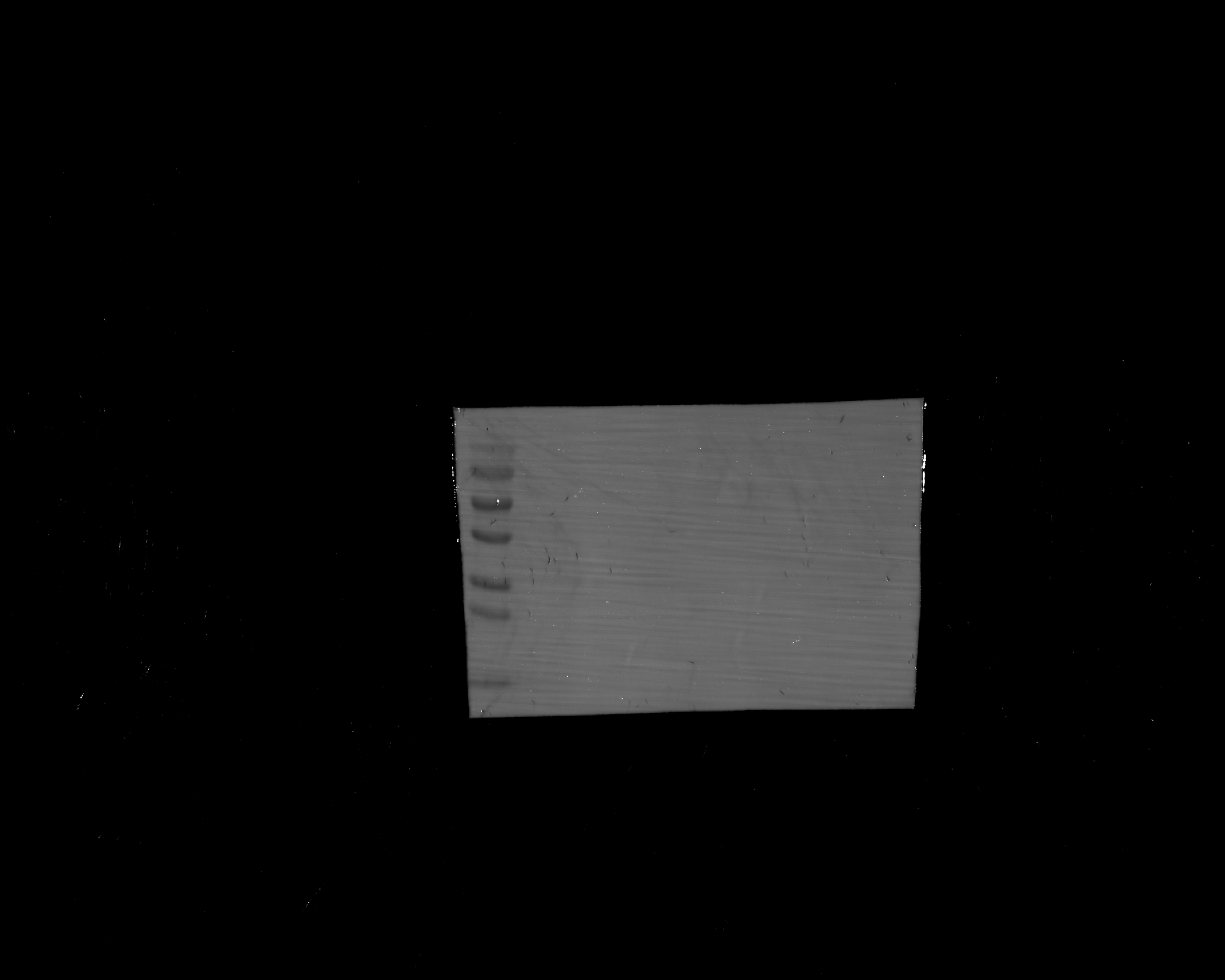

Supplement: Supplemental Information 6 [file peerj-12-18476-s006.zip › wb/repeat 2/gapdh 2(Colorimetric).tif]

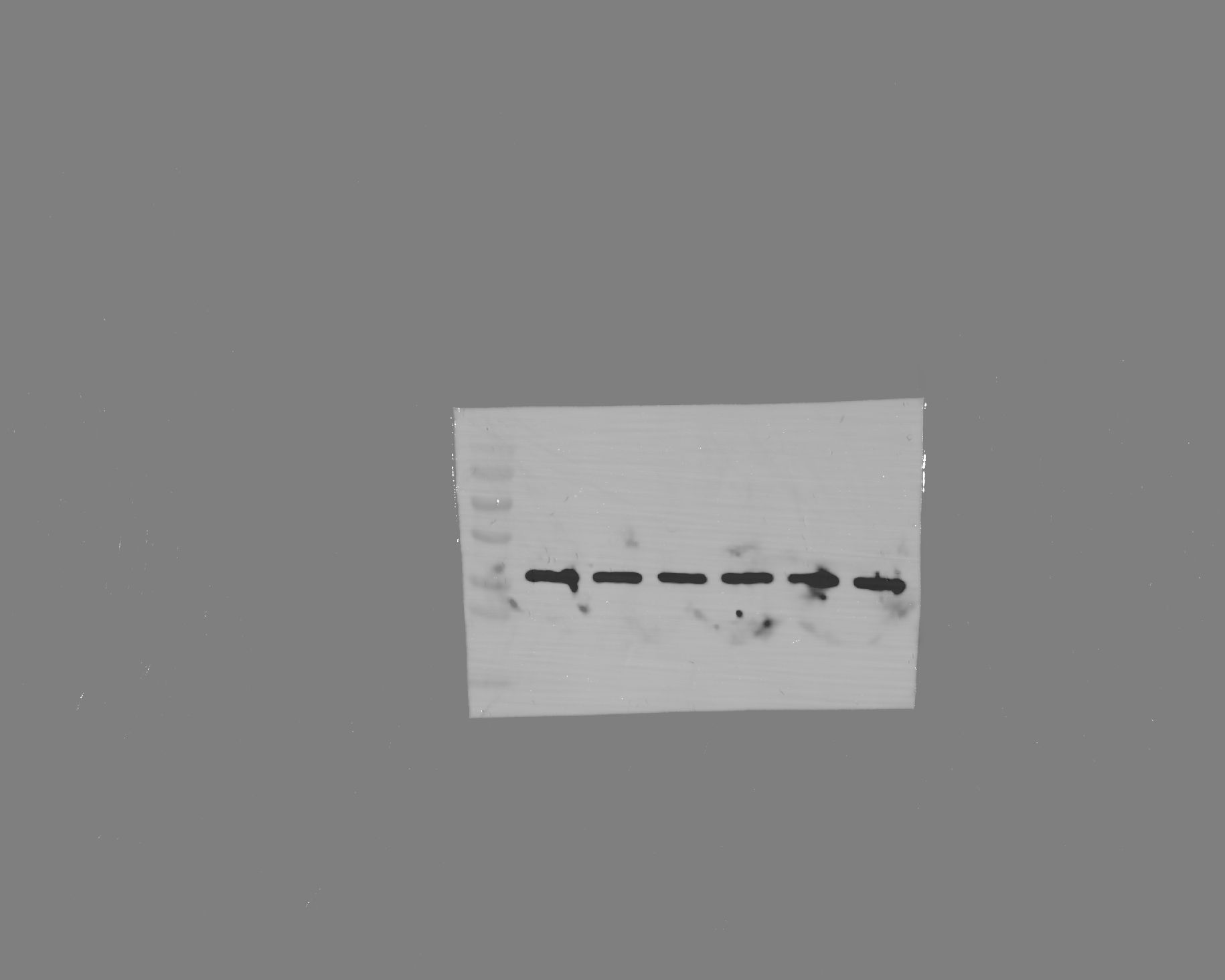

Supplement: Supplemental Information 6 [file peerj-12-18476-s006.zip › wb/repeat 2/gapdh 2(Composite).tif]

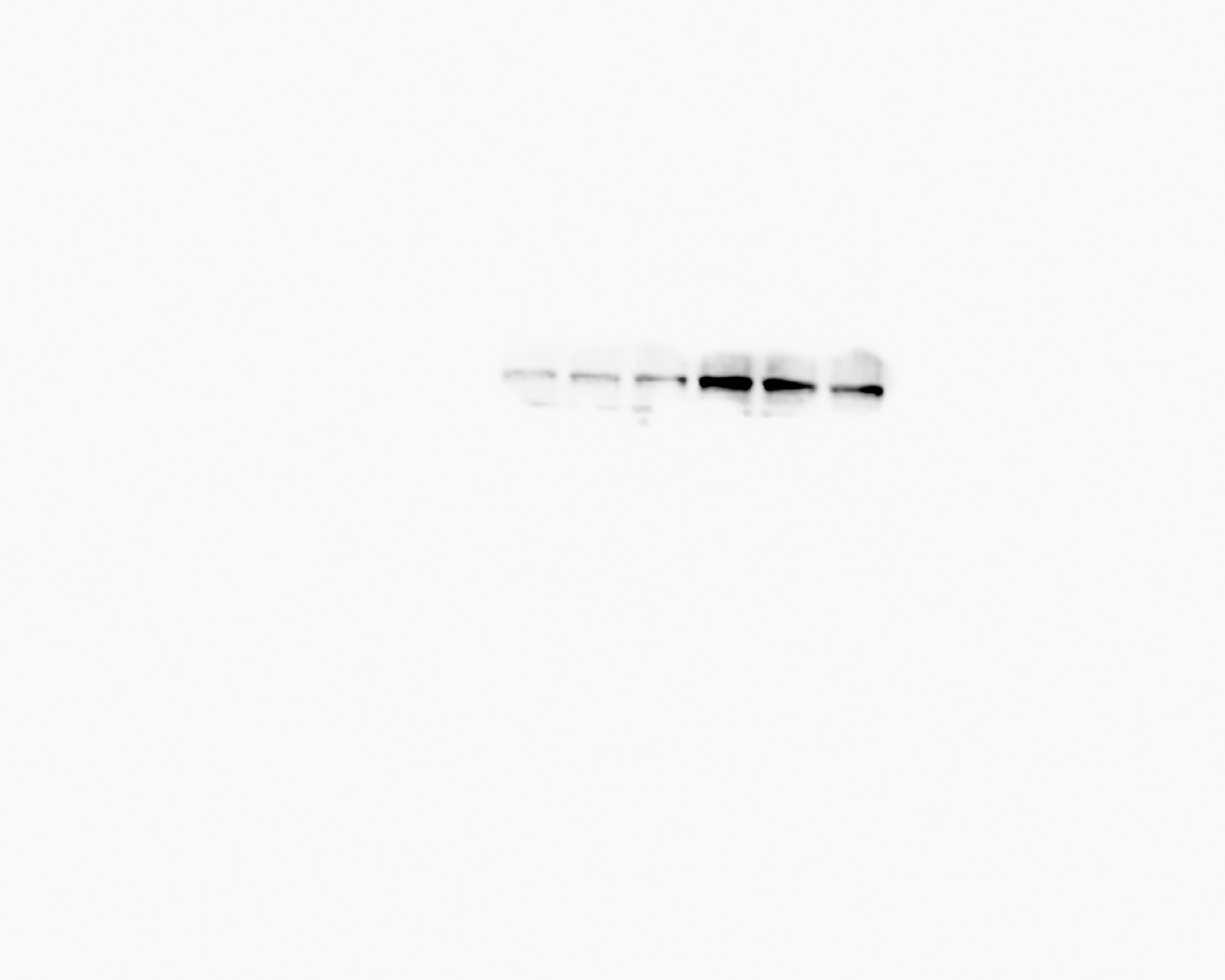

Supplement: Supplemental Information 6 [file peerj-12-18476-s006.zip › wb/repeat 2/vwf-2(Chemiluminescence).tif]

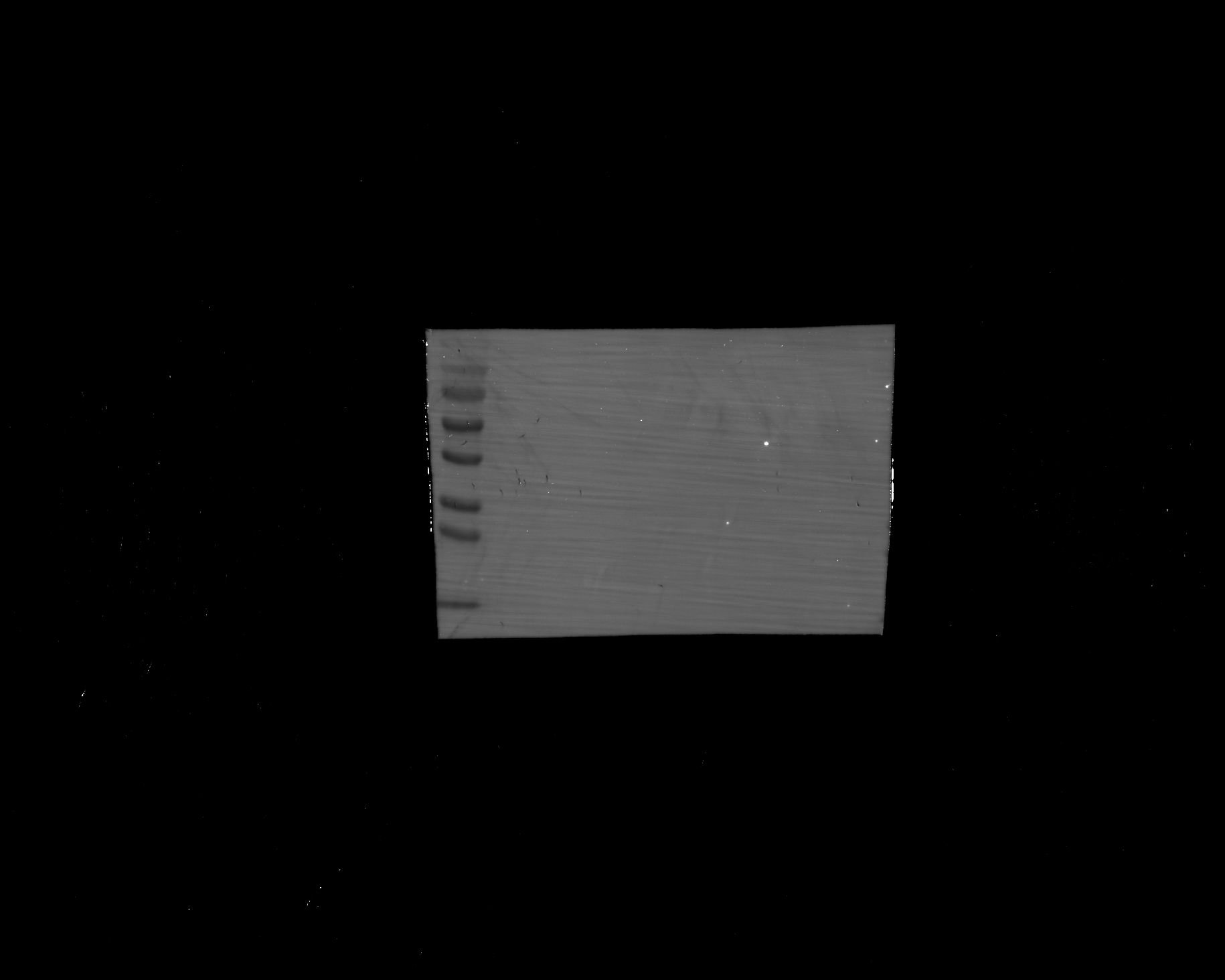

Supplement: Supplemental Information 6 [file peerj-12-18476-s006.zip › wb/repeat 2/vwf-2(Colorimetric).tif]

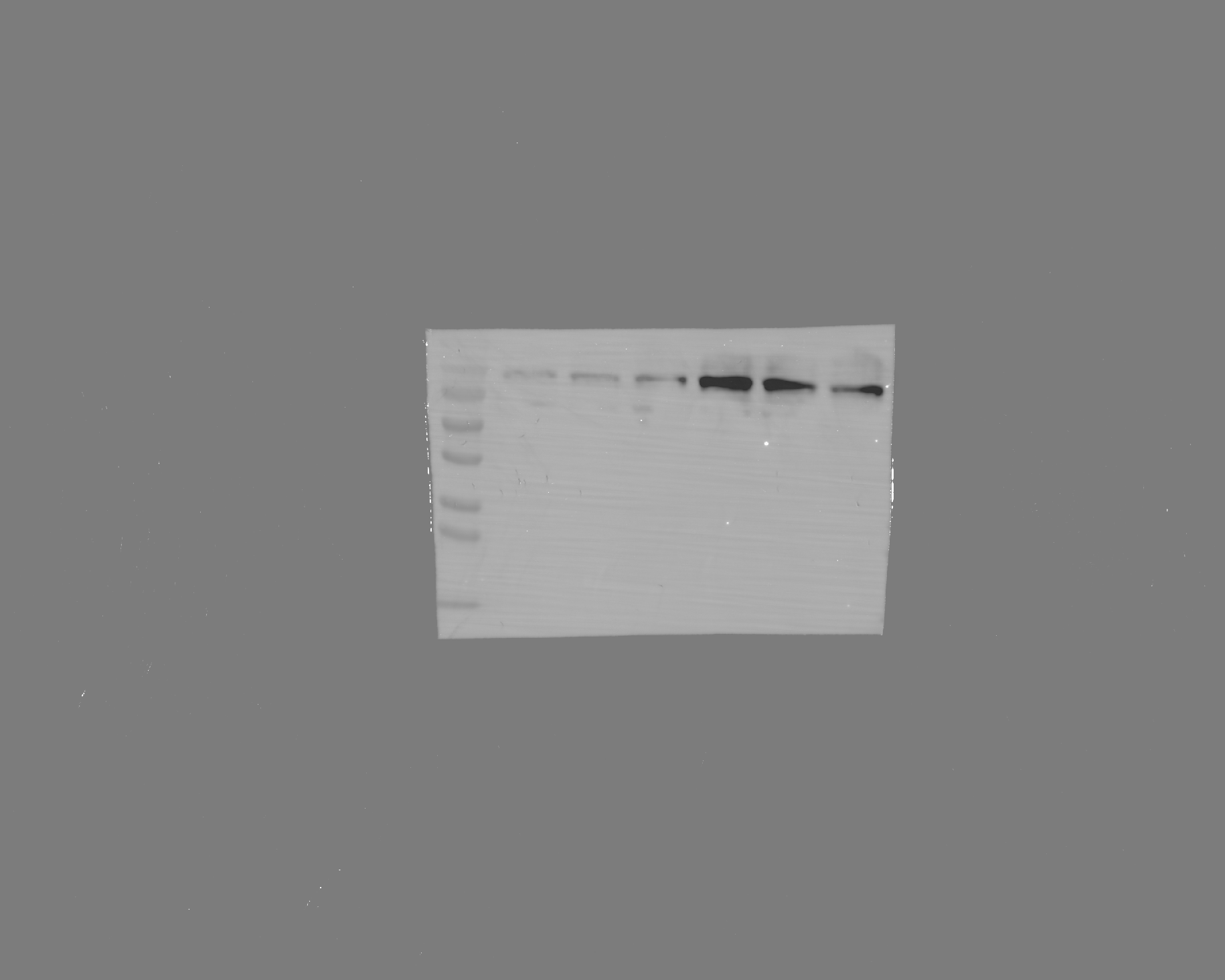

Supplement: Supplemental Information 6 [file peerj-12-18476-s006.zip › wb/repeat 2/vwf-2(Composite).tif]

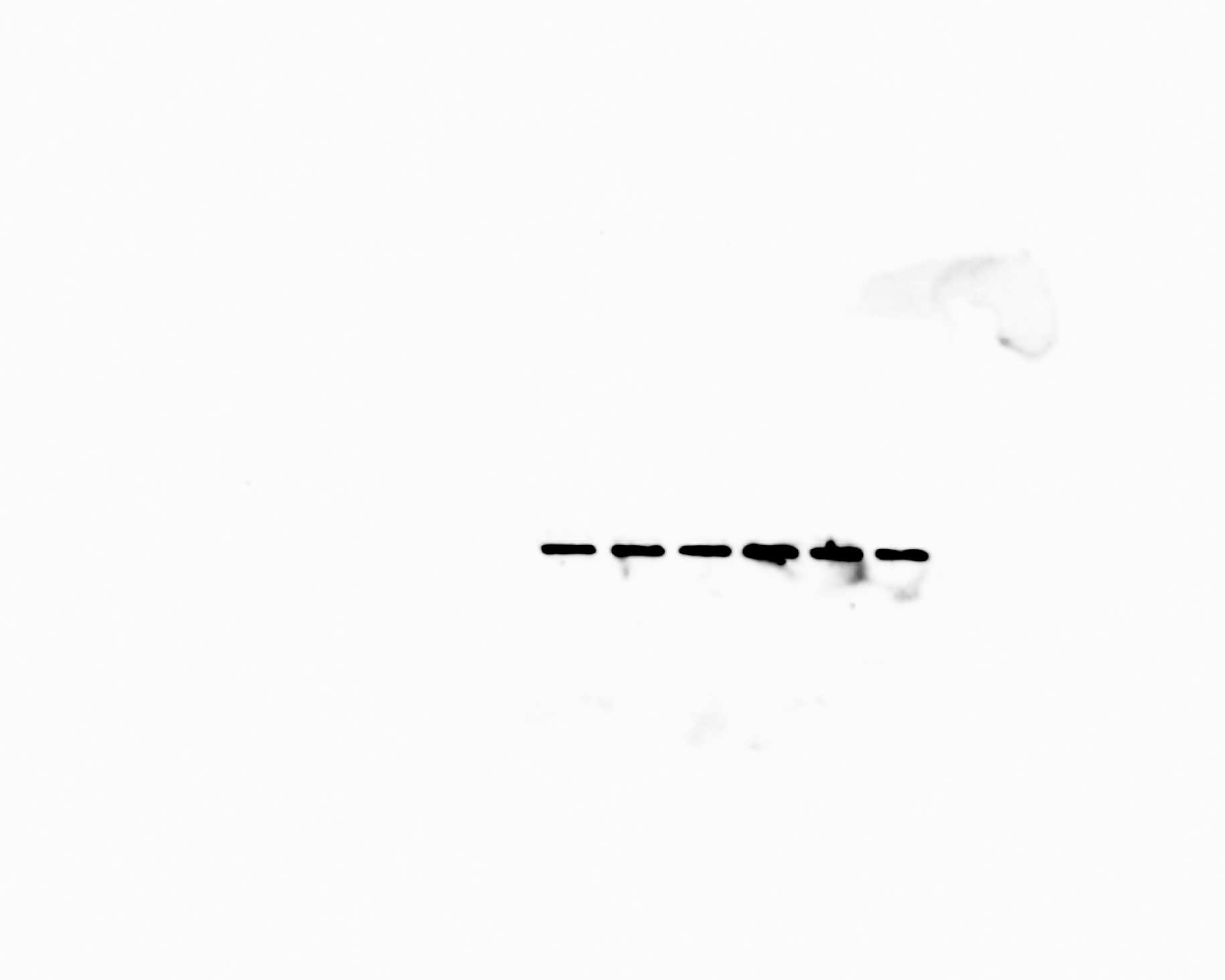

Supplement: Supplemental Information 6 [file peerj-12-18476-s006.zip › wb/repeat 3/gapdh 3(Chemiluminescence).tif]

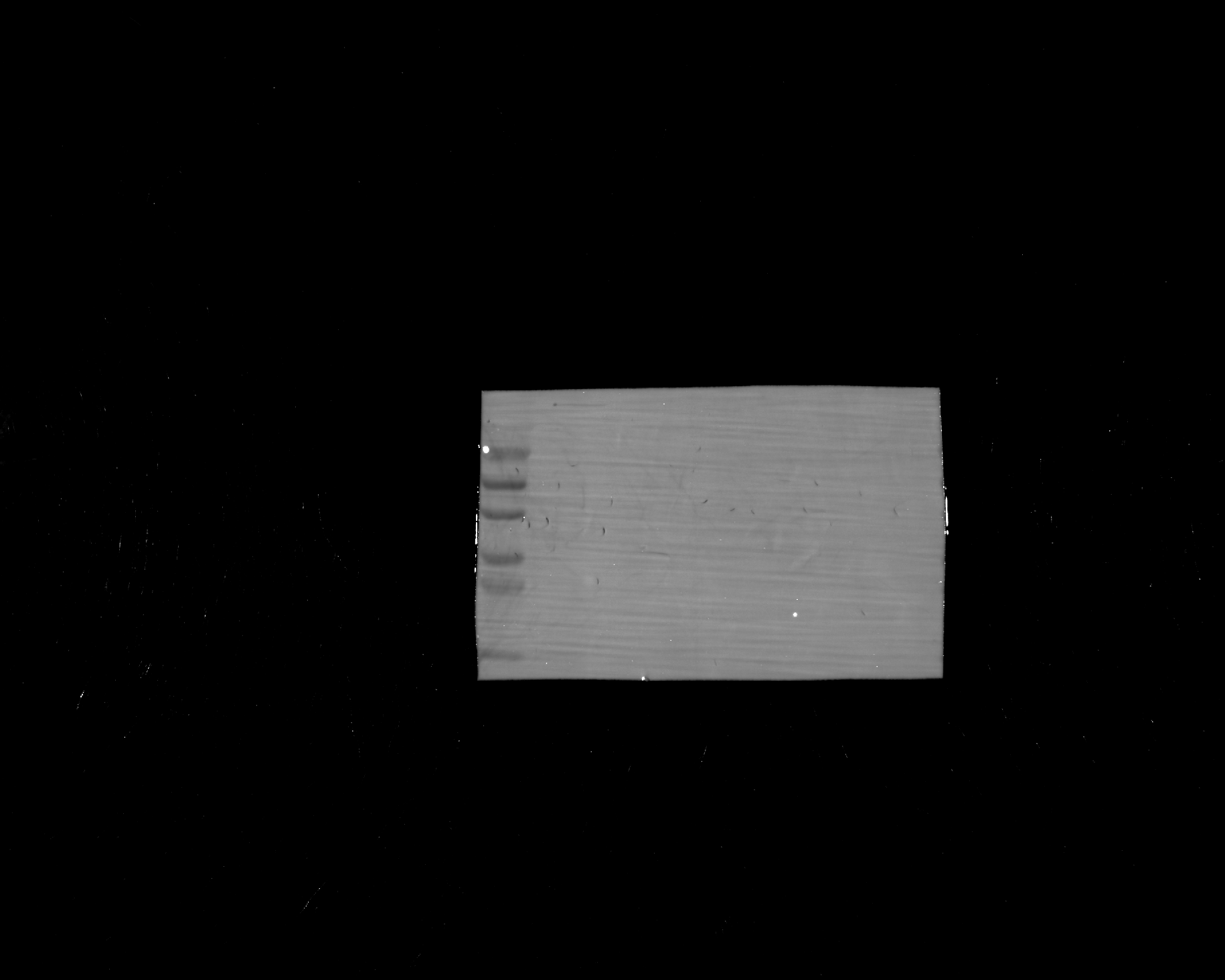

Supplement: Supplemental Information 6 [file peerj-12-18476-s006.zip › wb/repeat 3/gapdh 3(Colorimetric).tif]

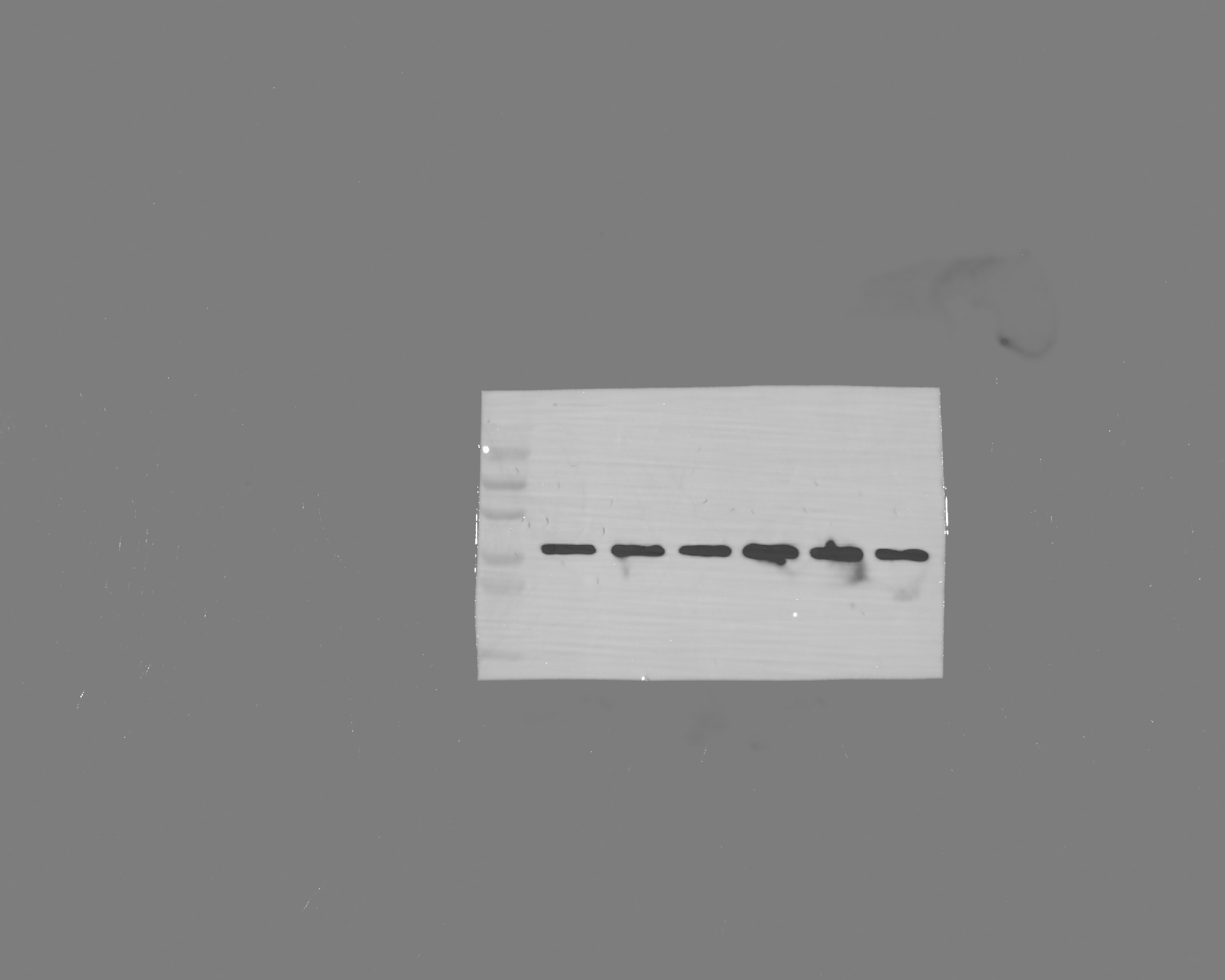

Supplement: Supplemental Information 6 [file peerj-12-18476-s006.zip › wb/repeat 3/gapdh 3(Composite).tif]

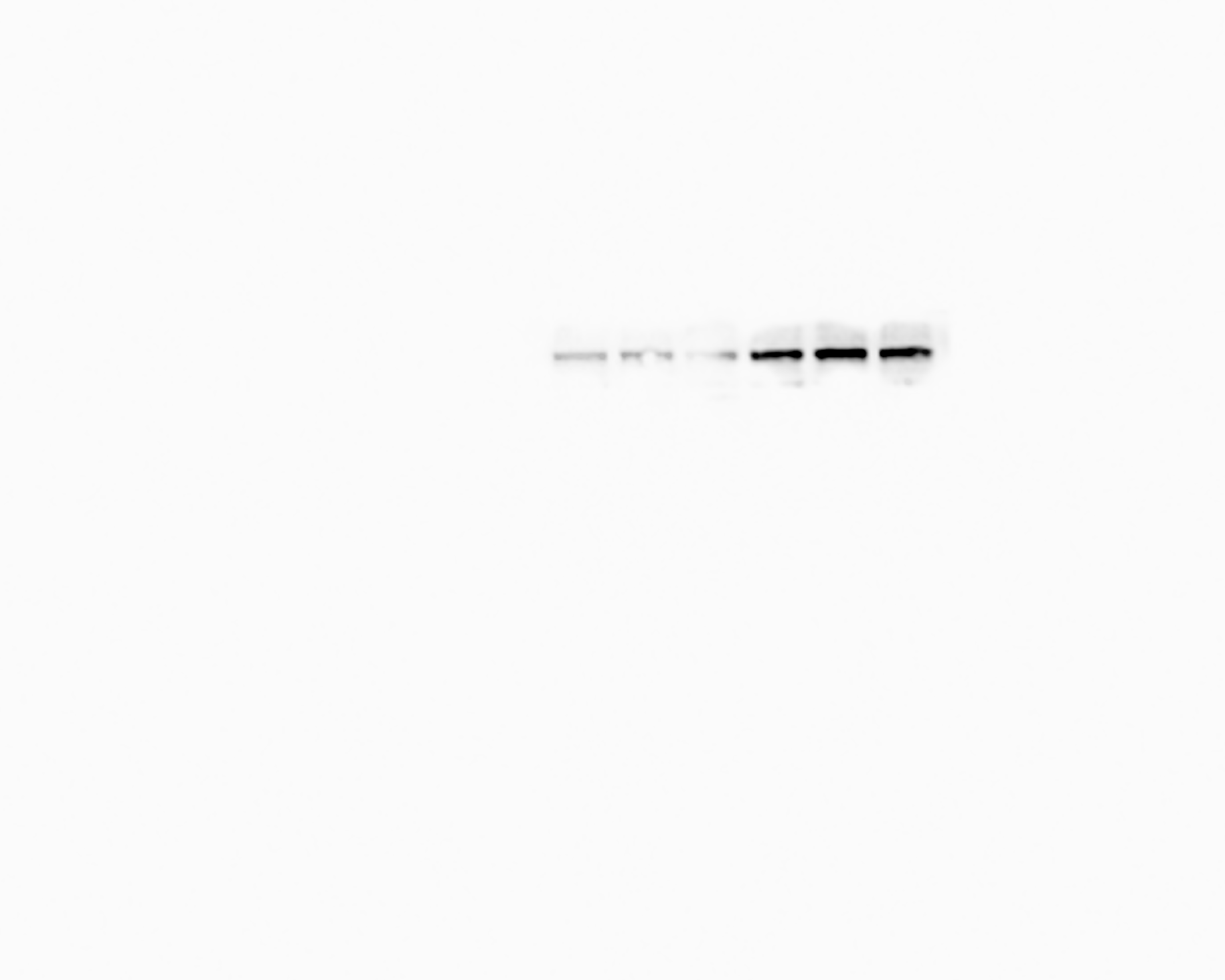

Supplement: Supplemental Information 6 [file peerj-12-18476-s006.zip › wb/repeat 3/vwf-3(Chemiluminescence).tif]

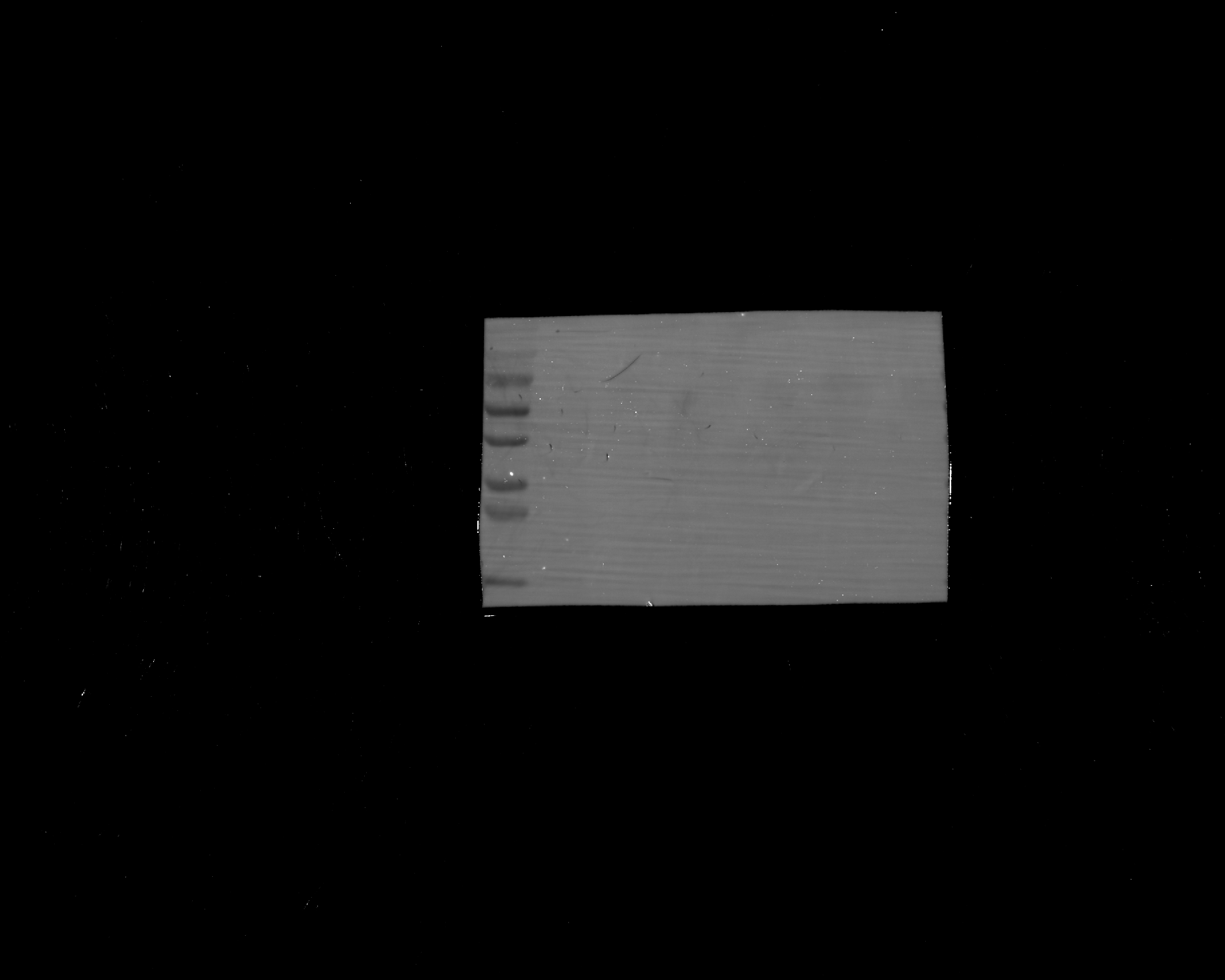

Supplement: Supplemental Information 6 [file peerj-12-18476-s006.zip › wb/repeat 3/vwf-3(Colorimetric).tif]

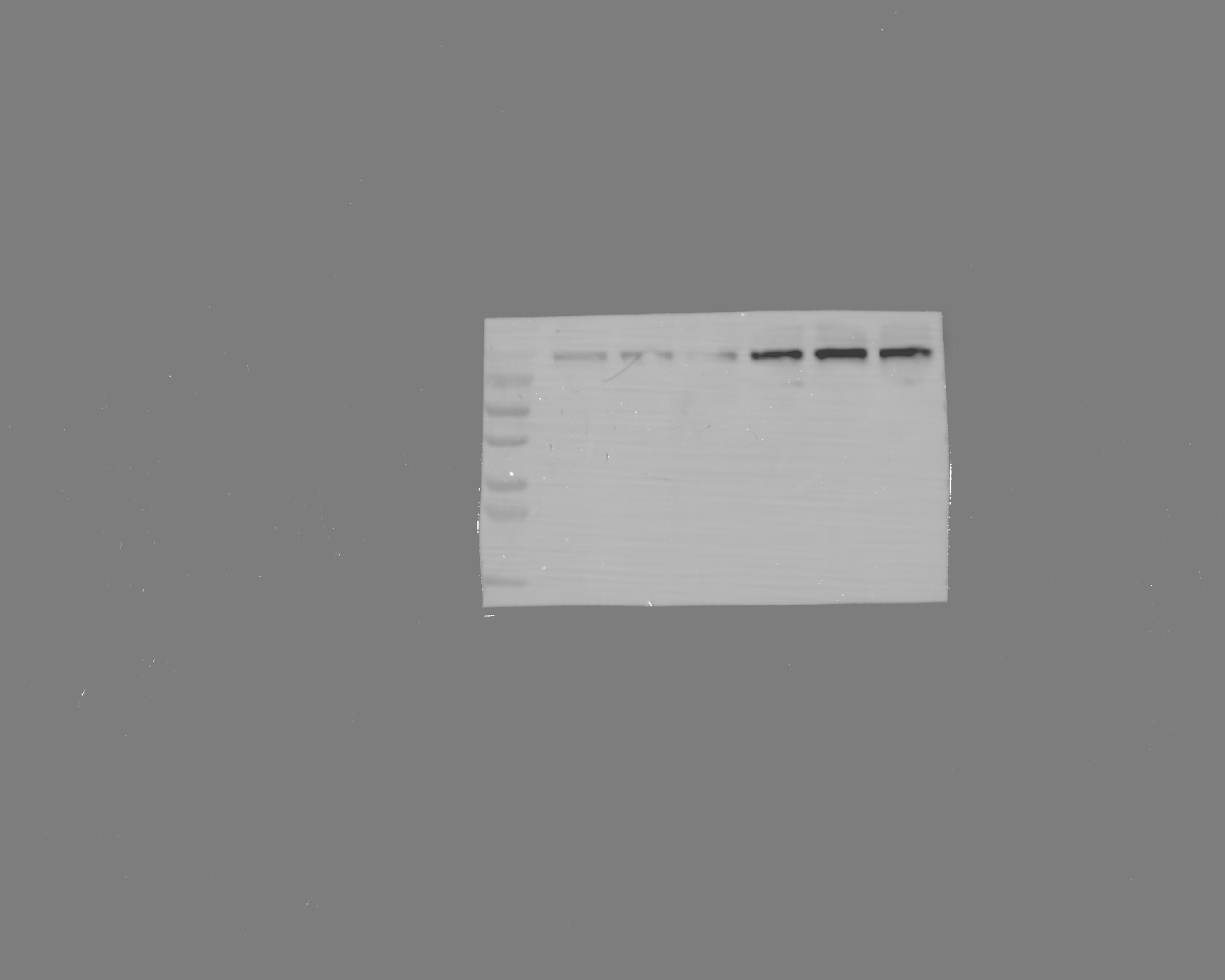

Supplement: Supplemental Information 6 [file peerj-12-18476-s006.zip › wb/repeat 3/vwf-3(Composite).tif]

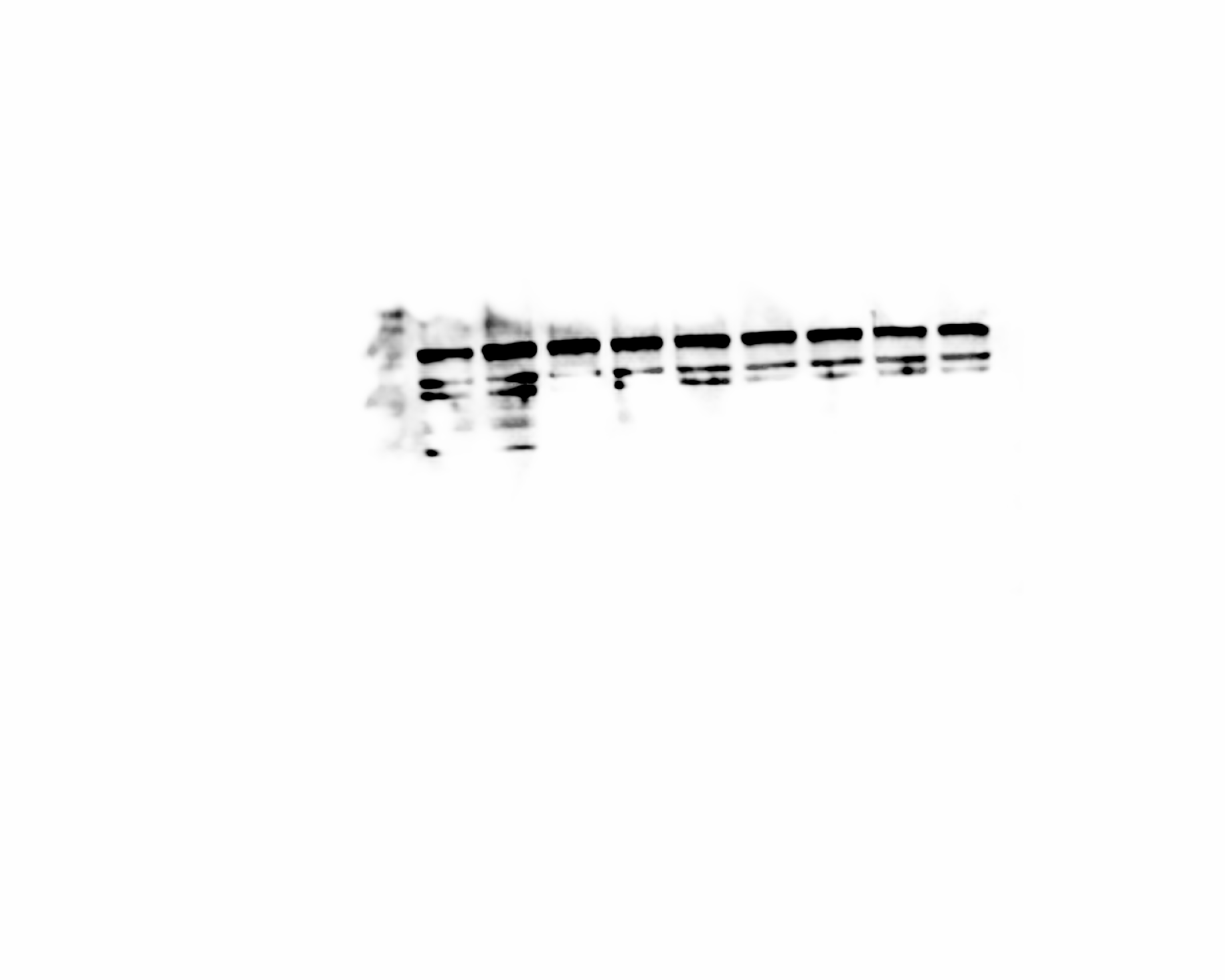

Supplement: Supplemental Information 8 [file peerj-12-18476-s008.zip › wb/Repeat 2/gapdh 2(Chemiluminescence).tif]

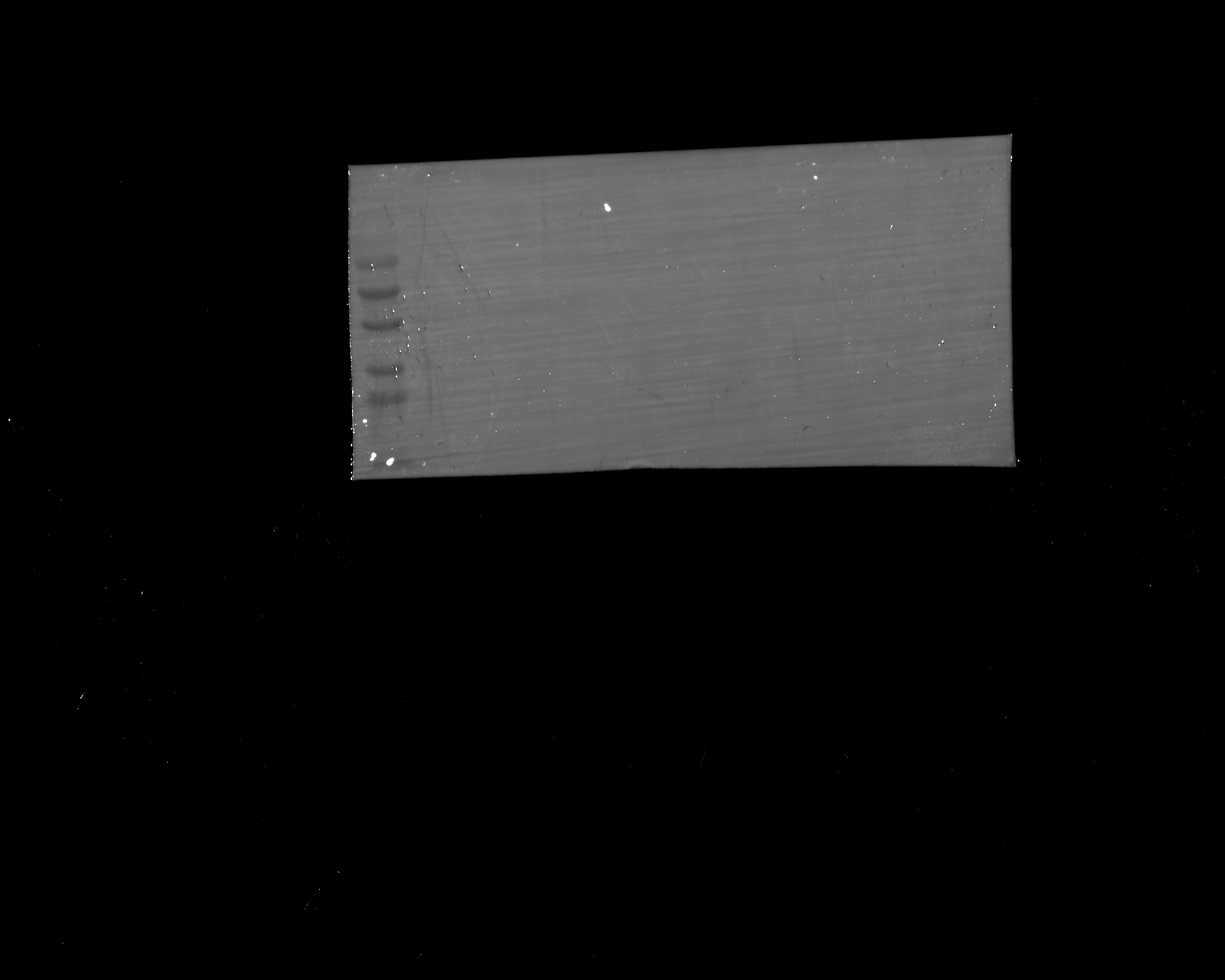

Supplement: Supplemental Information 8 [file peerj-12-18476-s008.zip › wb/Repeat 2/gapdh 2(Colorimetric).tif]

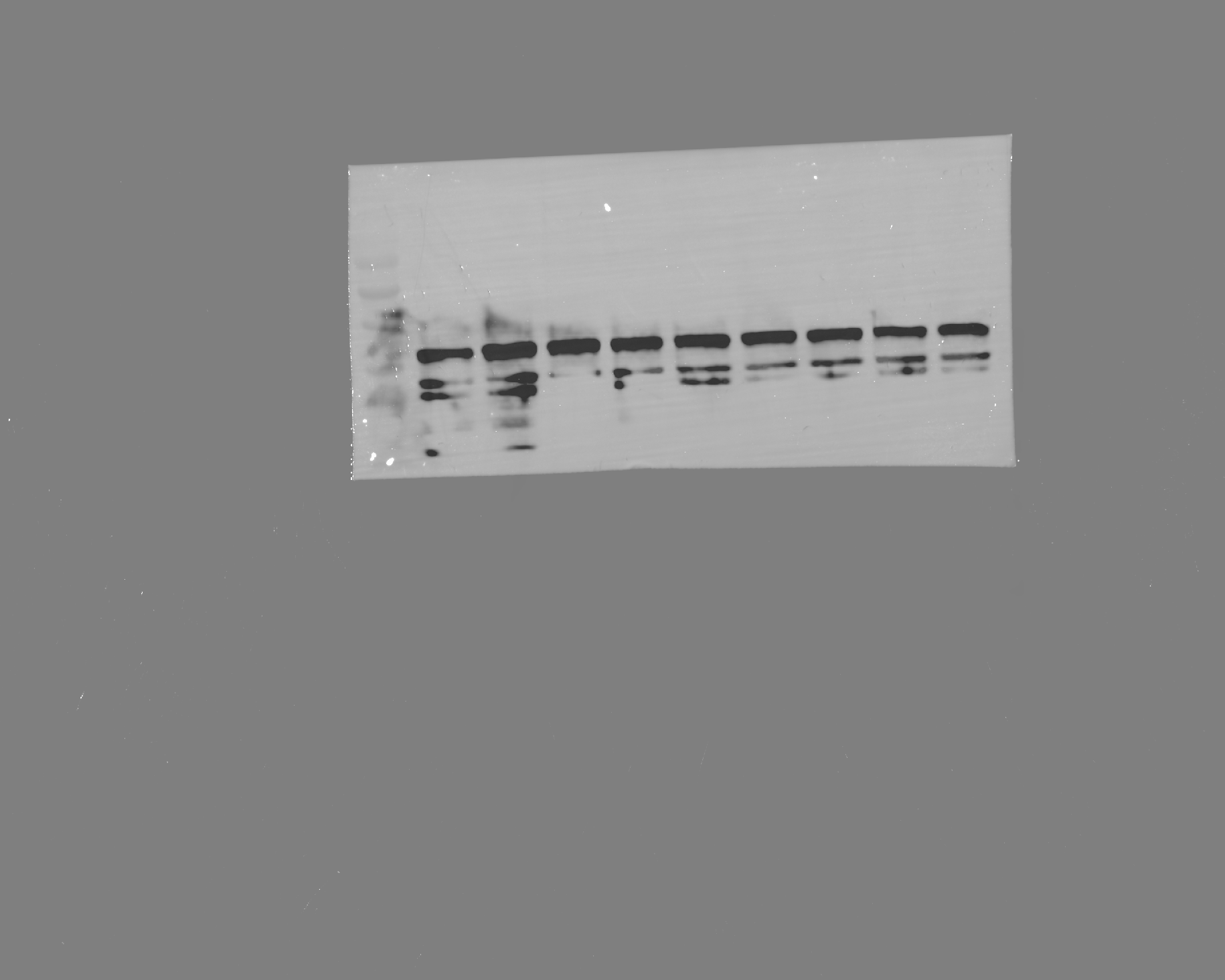

Supplement: Supplemental Information 8 [file peerj-12-18476-s008.zip › wb/Repeat 2/gapdh 2(Composite).tif]

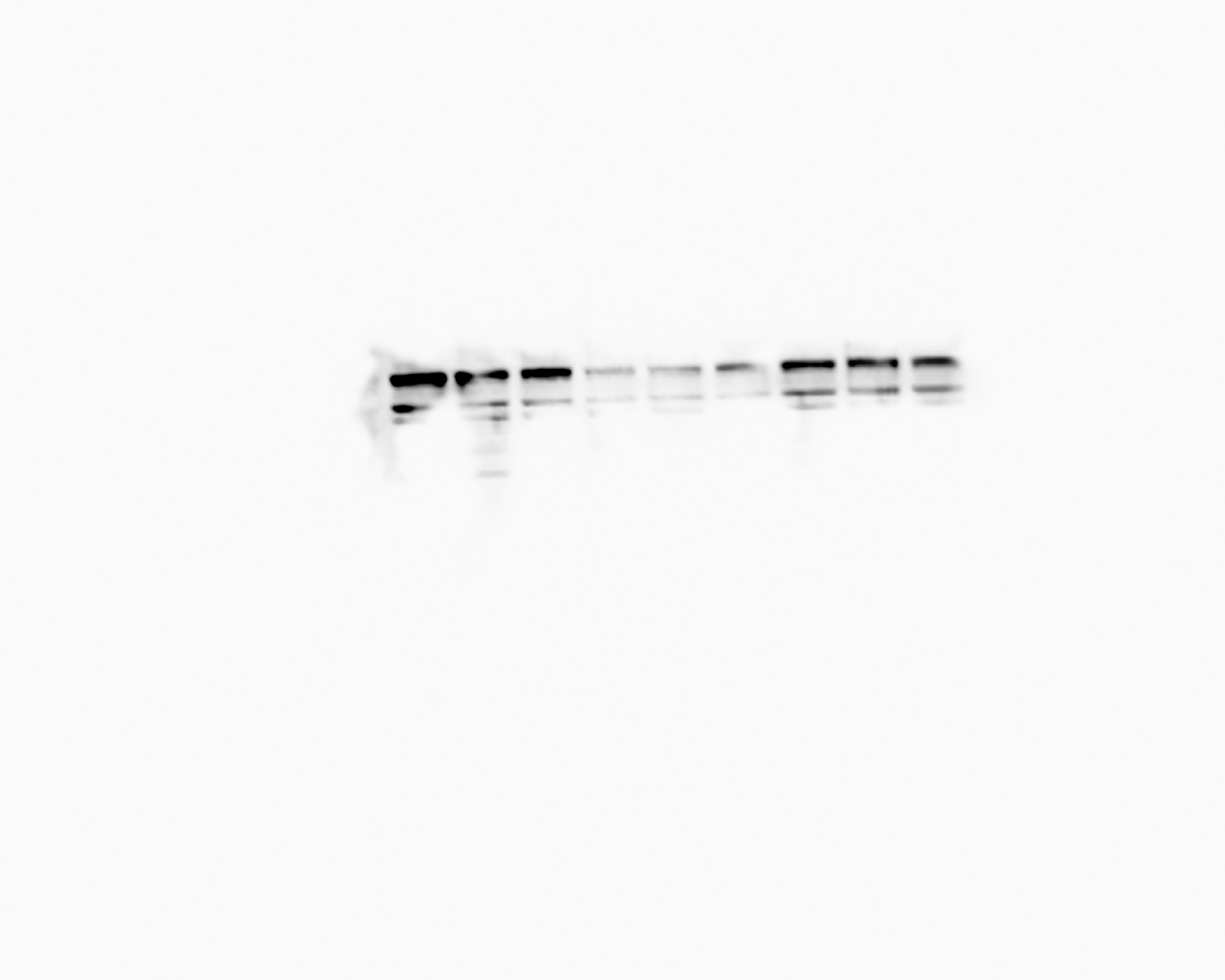

Supplement: Supplemental Information 8 [file peerj-12-18476-s008.zip › wb/Repeat 2/vwf-2(Chemiluminescence).tif]

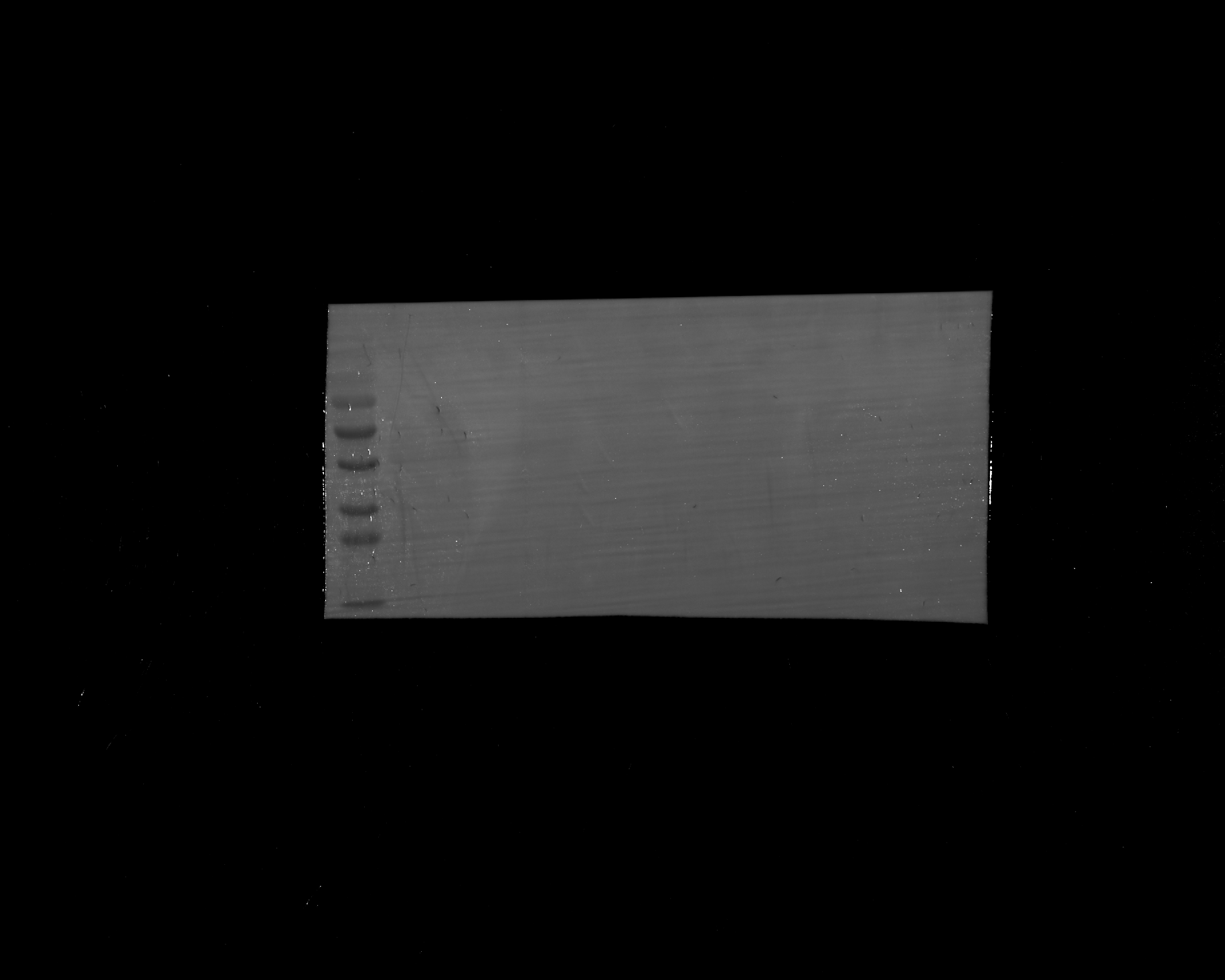

Supplement: Supplemental Information 8 [file peerj-12-18476-s008.zip › wb/Repeat 2/vwf-2(Colorimetric).tif]

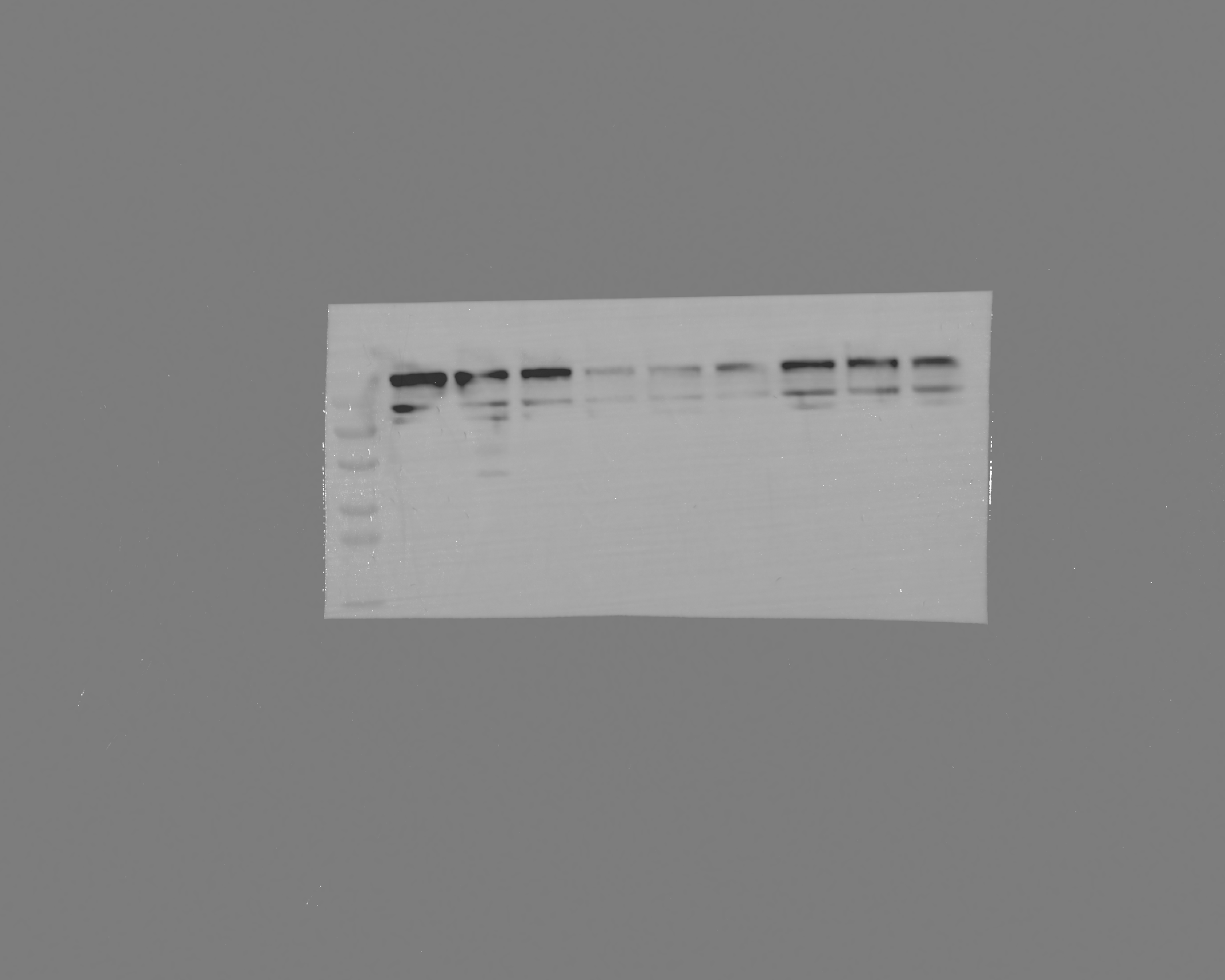

Supplement: Supplemental Information 8 [file peerj-12-18476-s008.zip › wb/Repeat 2/vwf-2(Composite).tif]

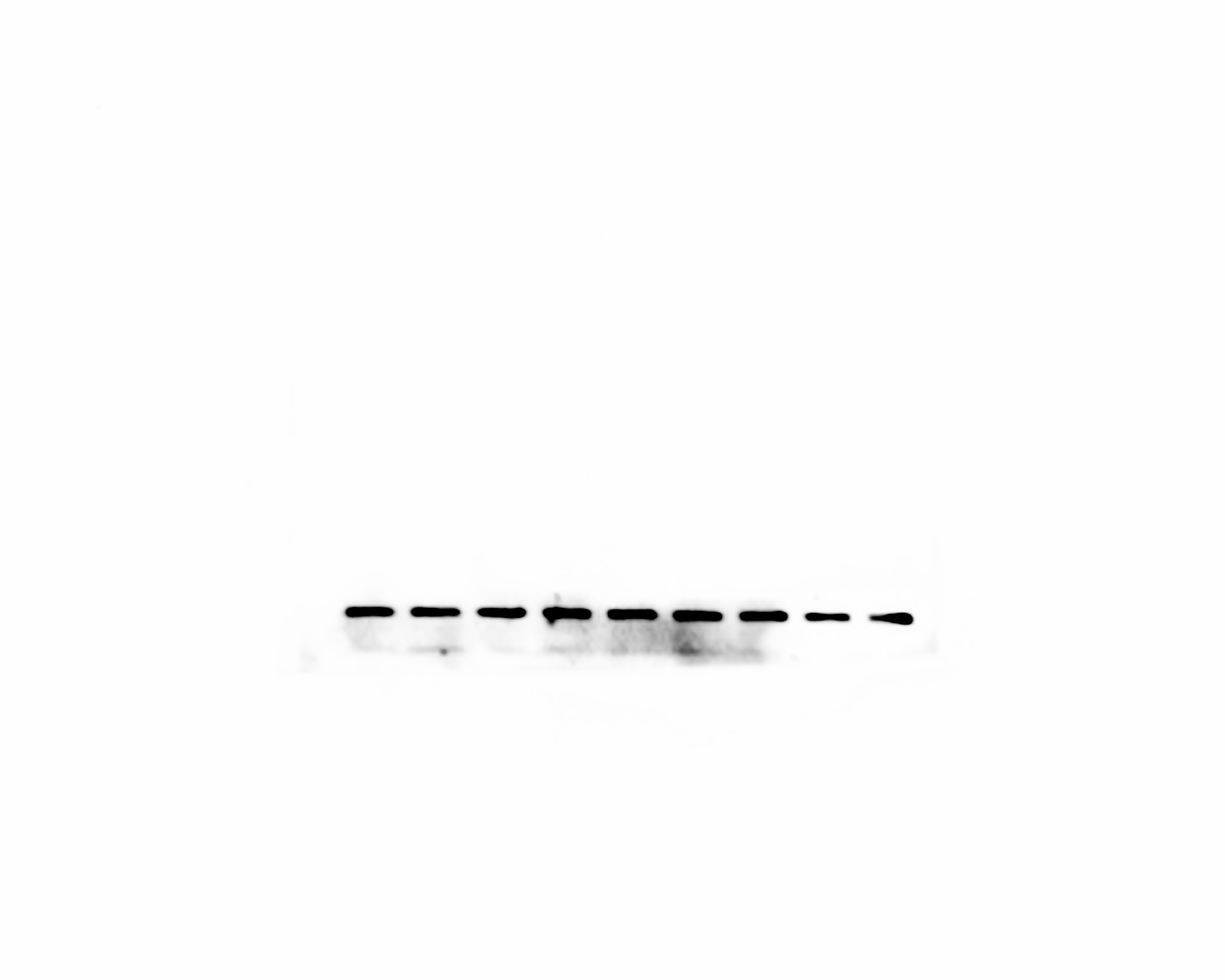

Supplement: Supplemental Information 8 [file peerj-12-18476-s008.zip › wb/Repeat 3/gapdh 3(Chemiluminescence).tif]

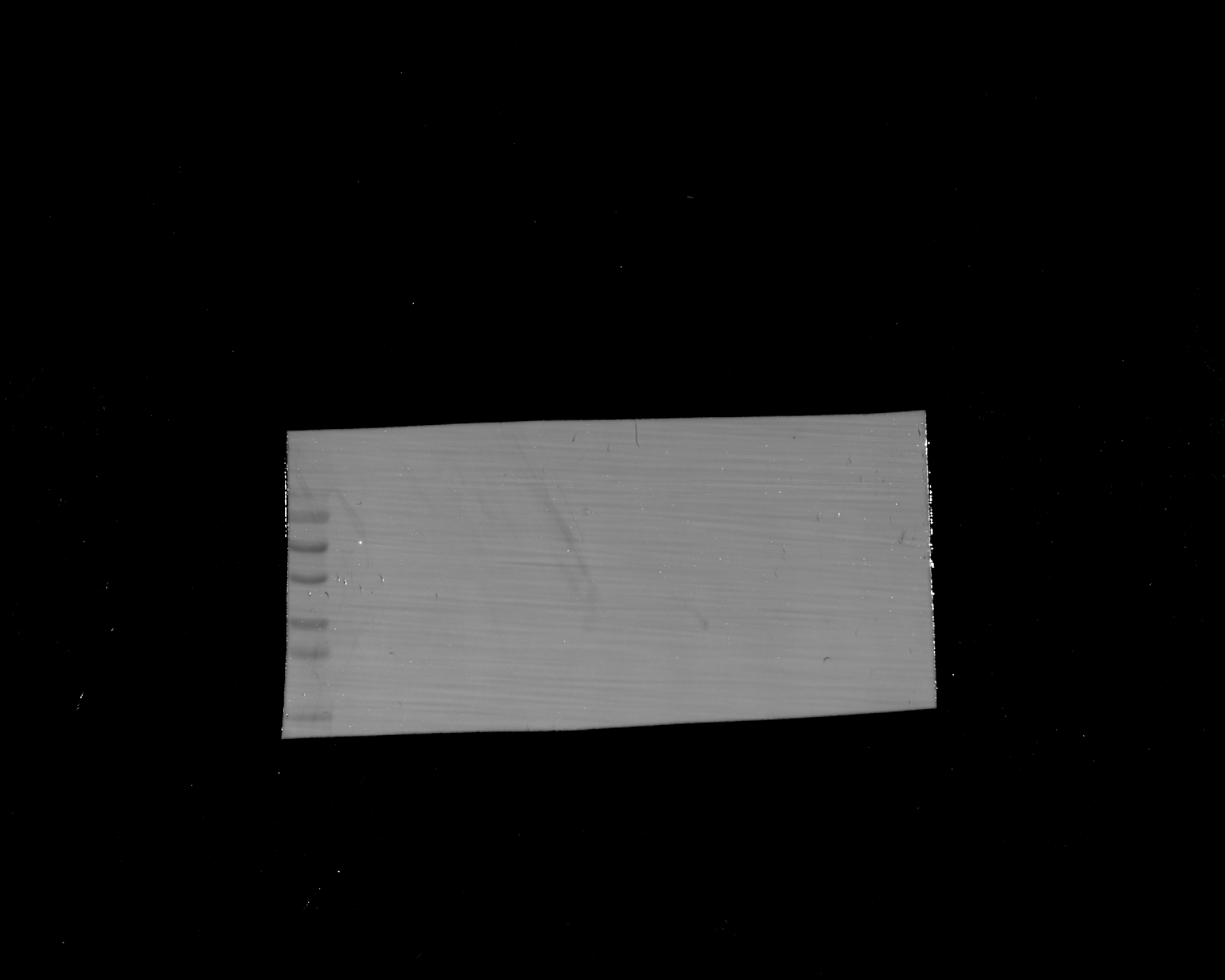

Supplement: Supplemental Information 8 [file peerj-12-18476-s008.zip › wb/Repeat 3/gapdh 3(Colorimetric).tif]

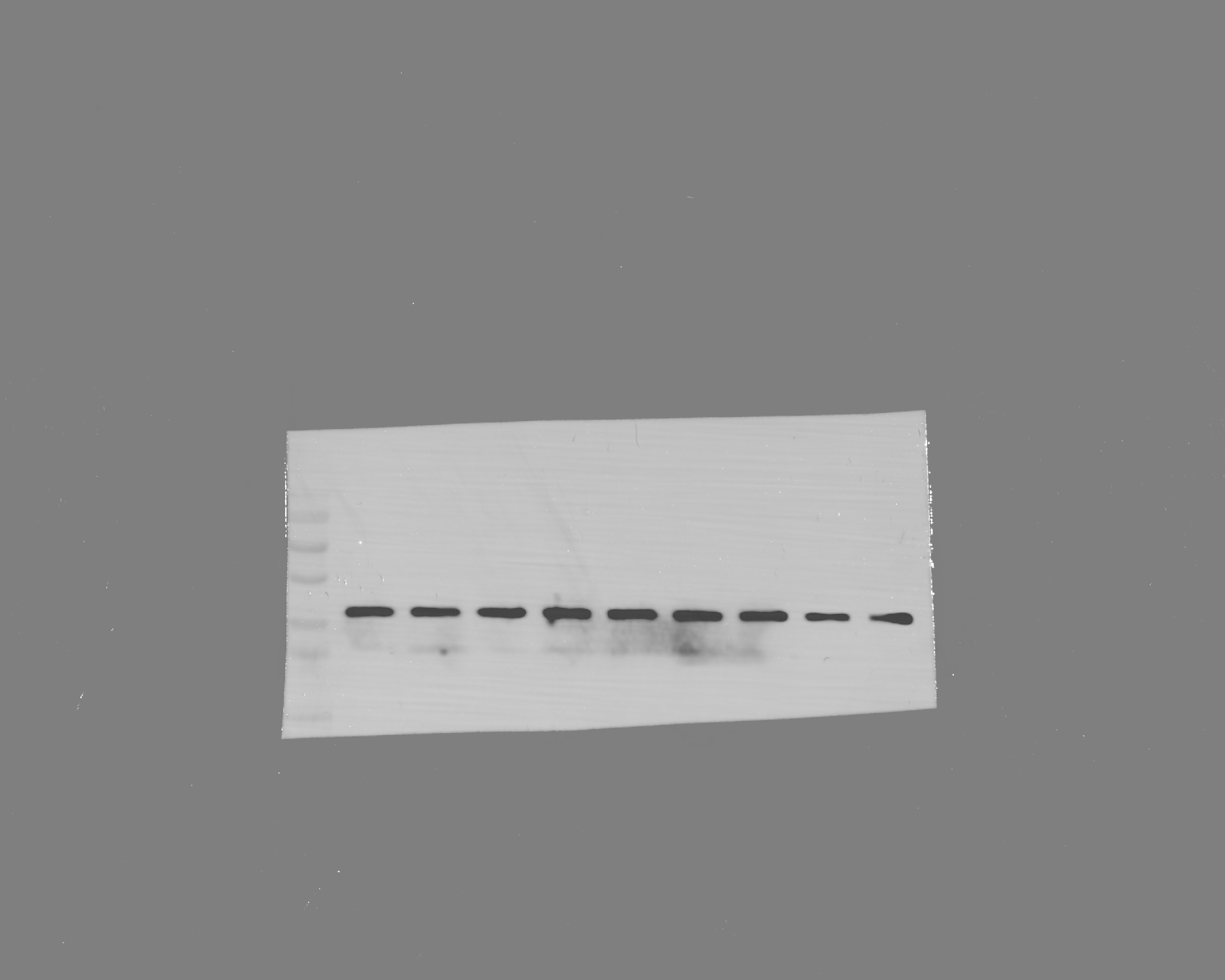

Supplement: Supplemental Information 8 [file peerj-12-18476-s008.zip › wb/Repeat 3/gapdh 3(Composite).tif]

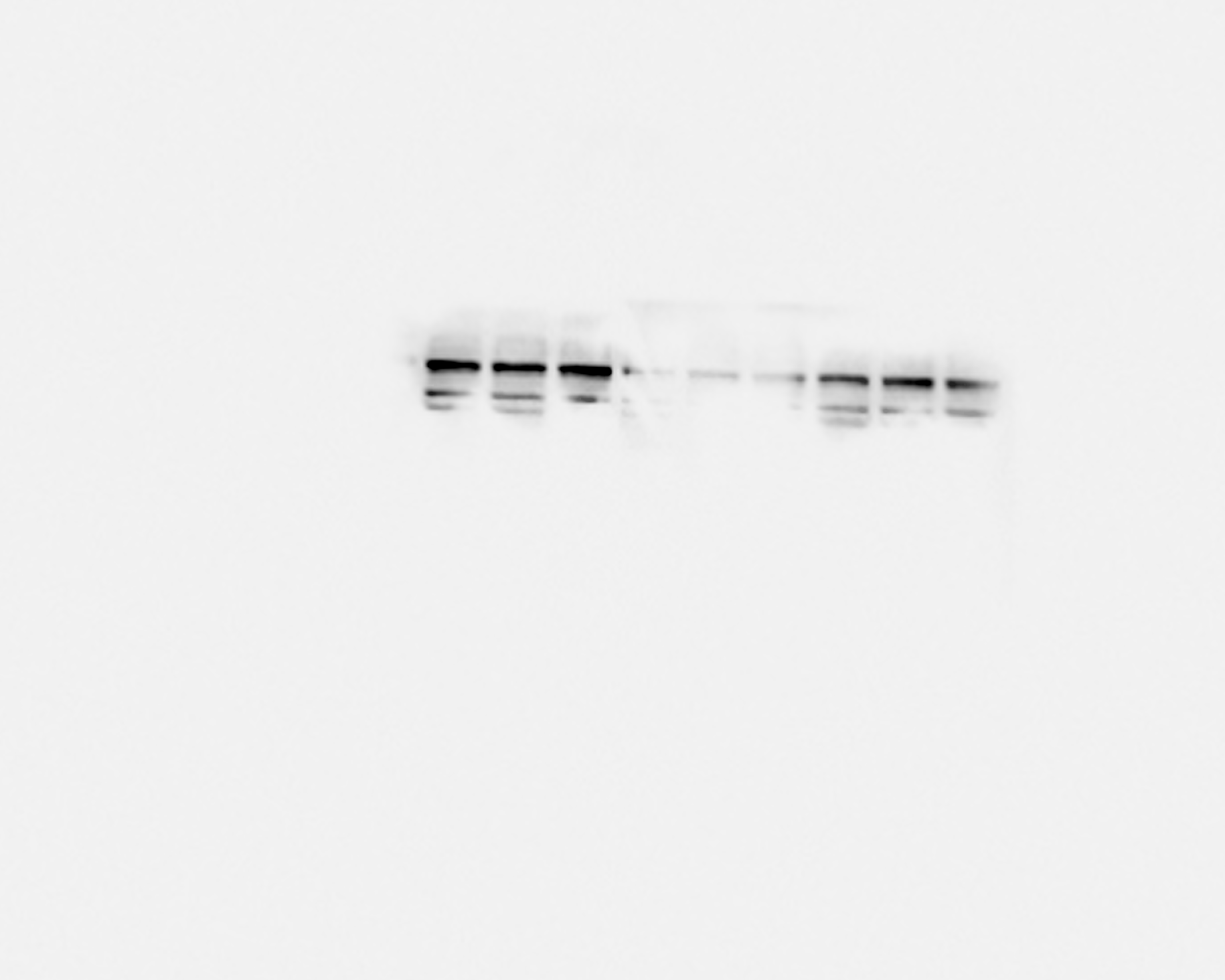

Supplement: Supplemental Information 8 [file peerj-12-18476-s008.zip › wb/Repeat 3/vwf-3(Chemiluminescence).tif]

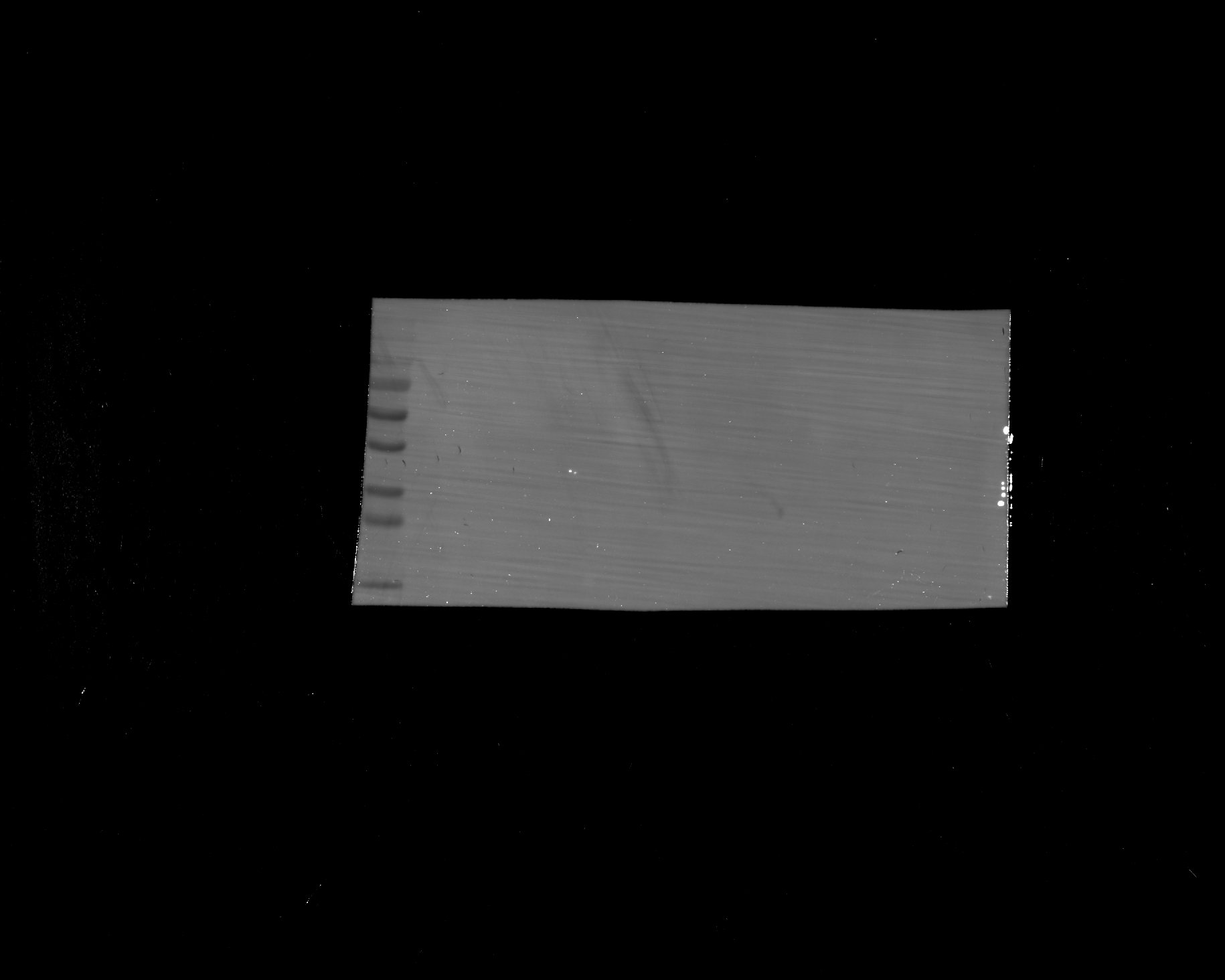

Supplement: Supplemental Information 8 [file peerj-12-18476-s008.zip › wb/Repeat 3/vwf-3(Colorimetric).tif]

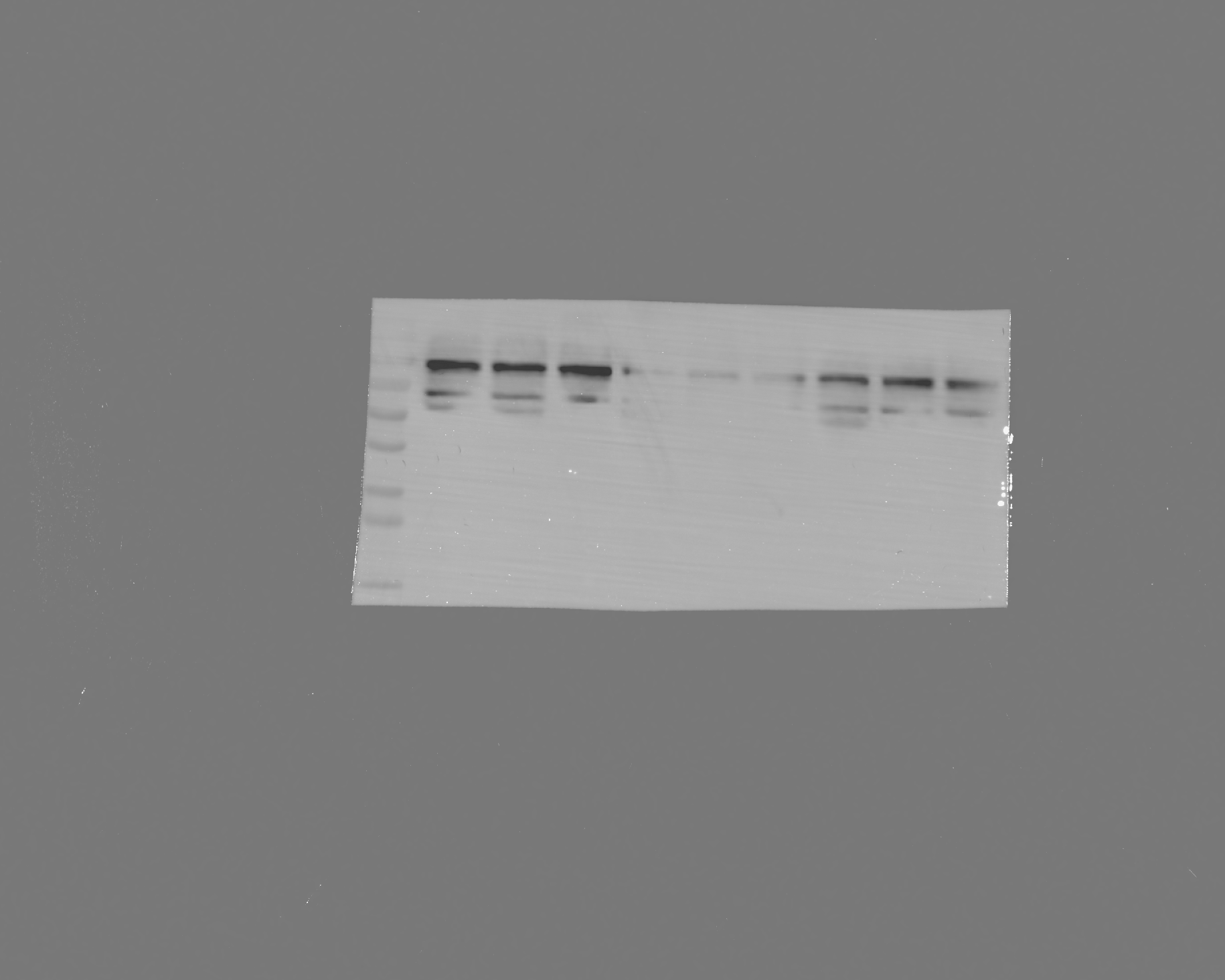

Supplement: Supplemental Information 8 [file peerj-12-18476-s008.zip › wb/Repeat 3/vwf-3(Composite).tif]

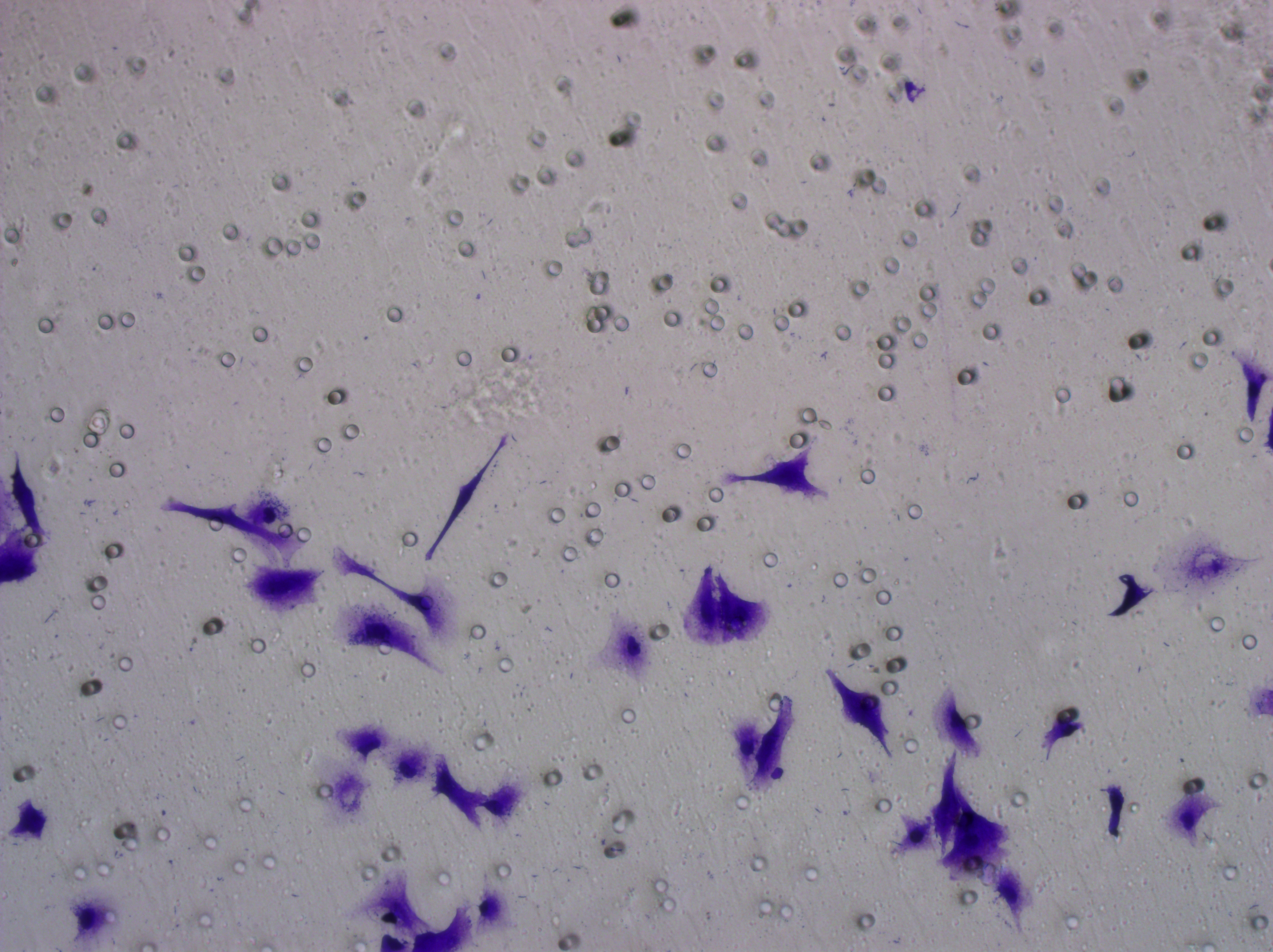

Supplement: Supplemental Information 10 [file peerj-12-18476-s010.png]

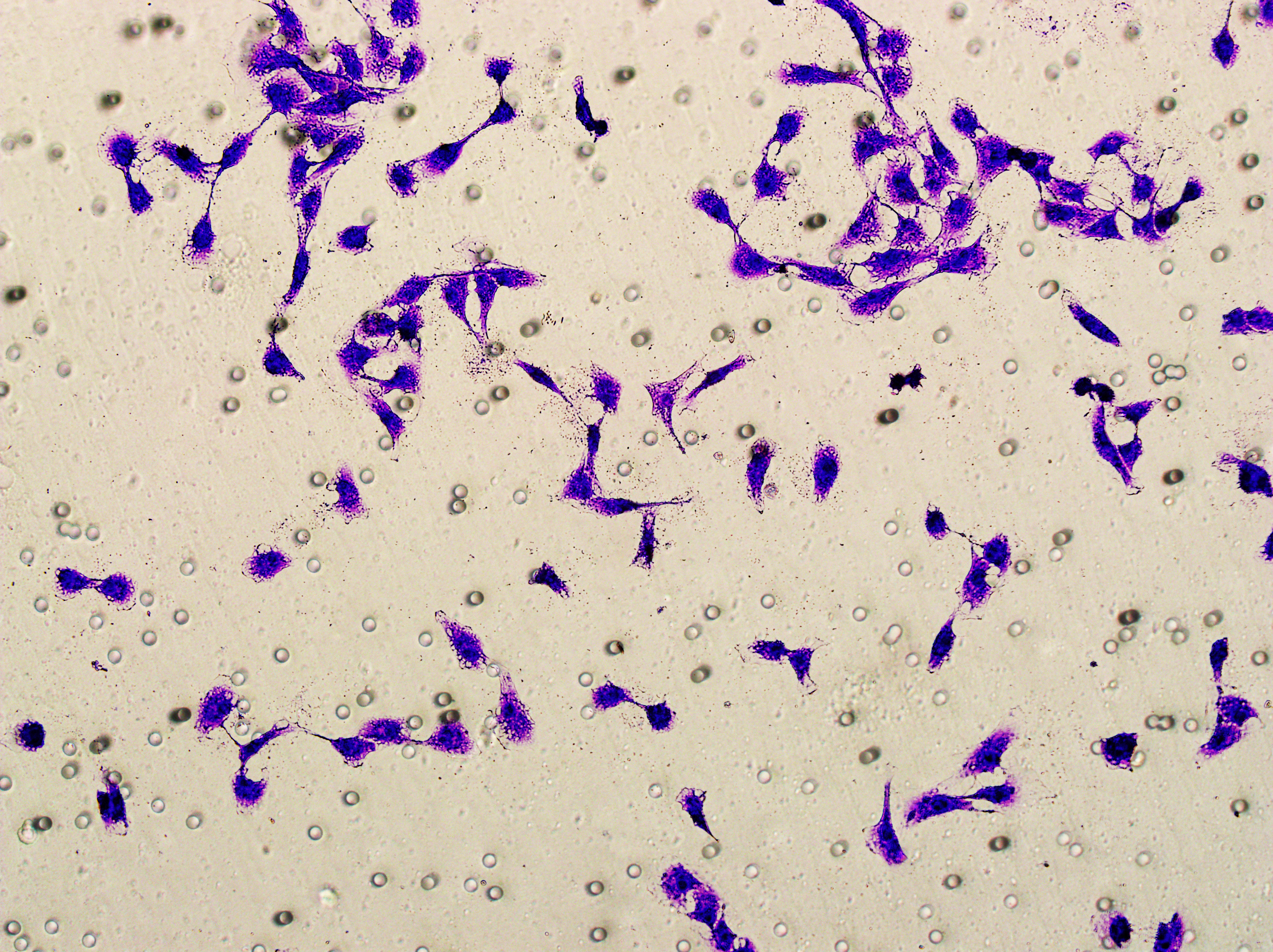

Supplement: Supplemental Information 11 [file peerj-12-18476-s011.png]

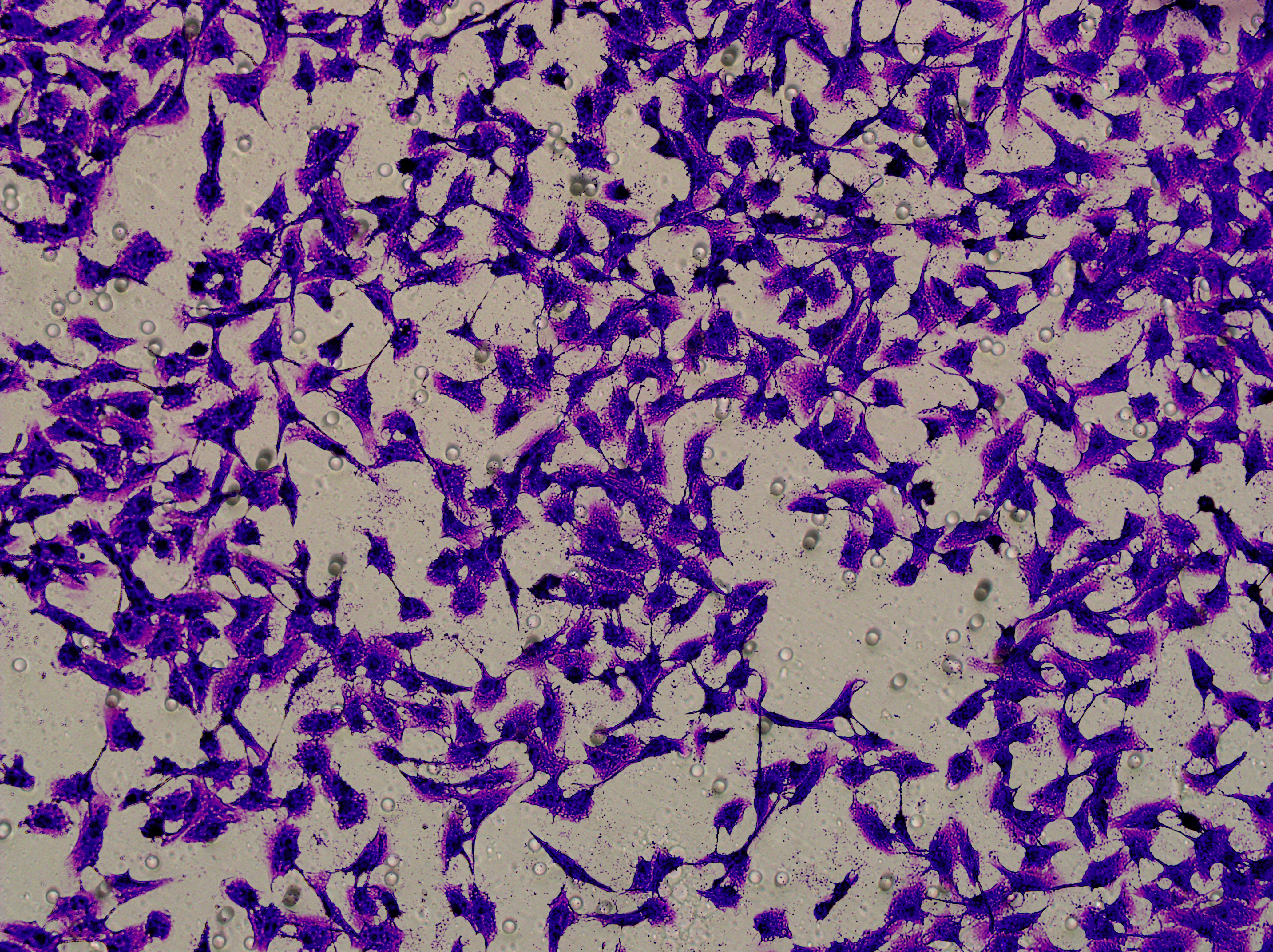

Supplement: Supplemental Information 12 [file peerj-12-18476-s012.png]

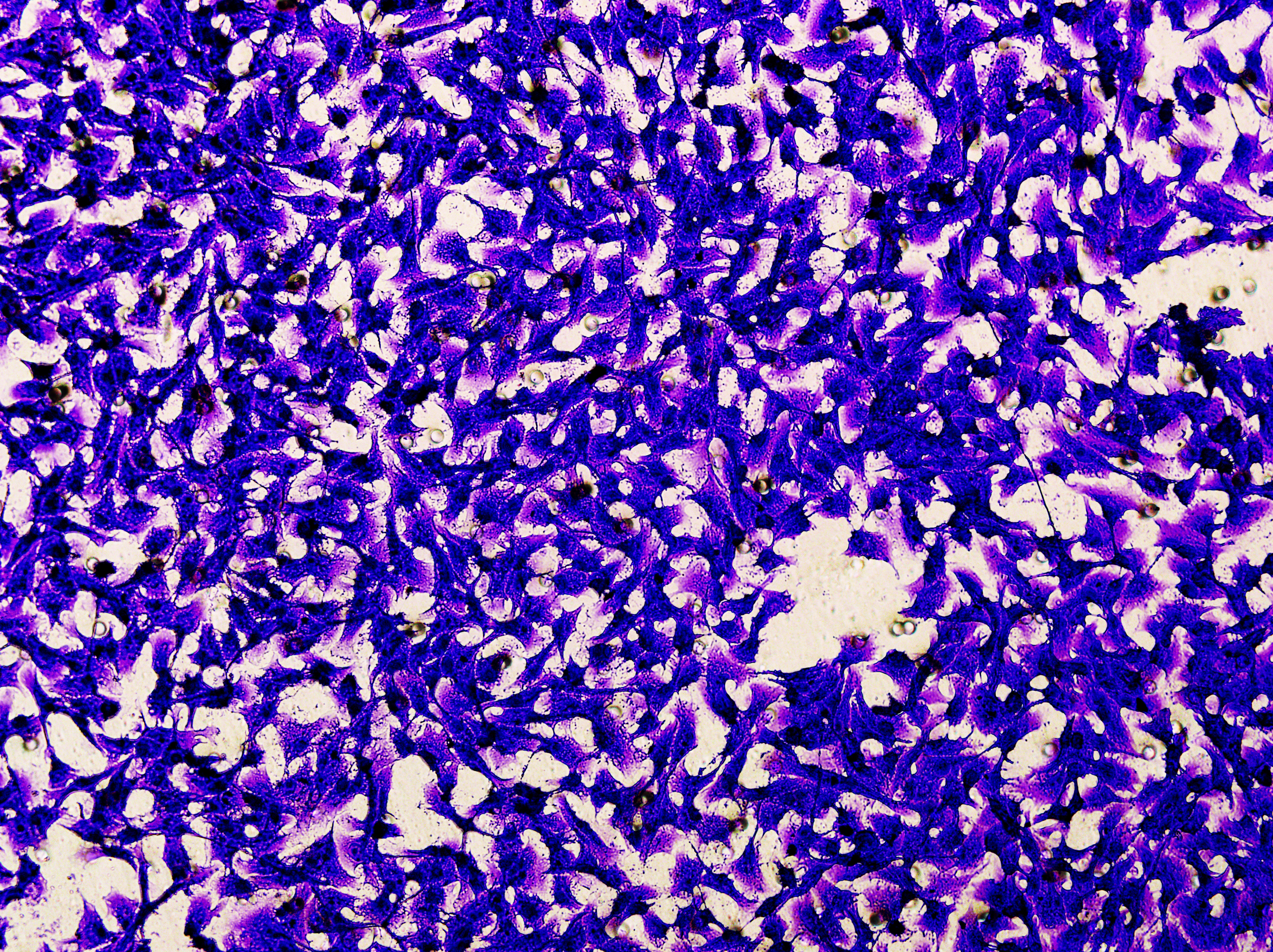

Supplement: Supplemental Information 13 [file peerj-12-18476-s013.png]

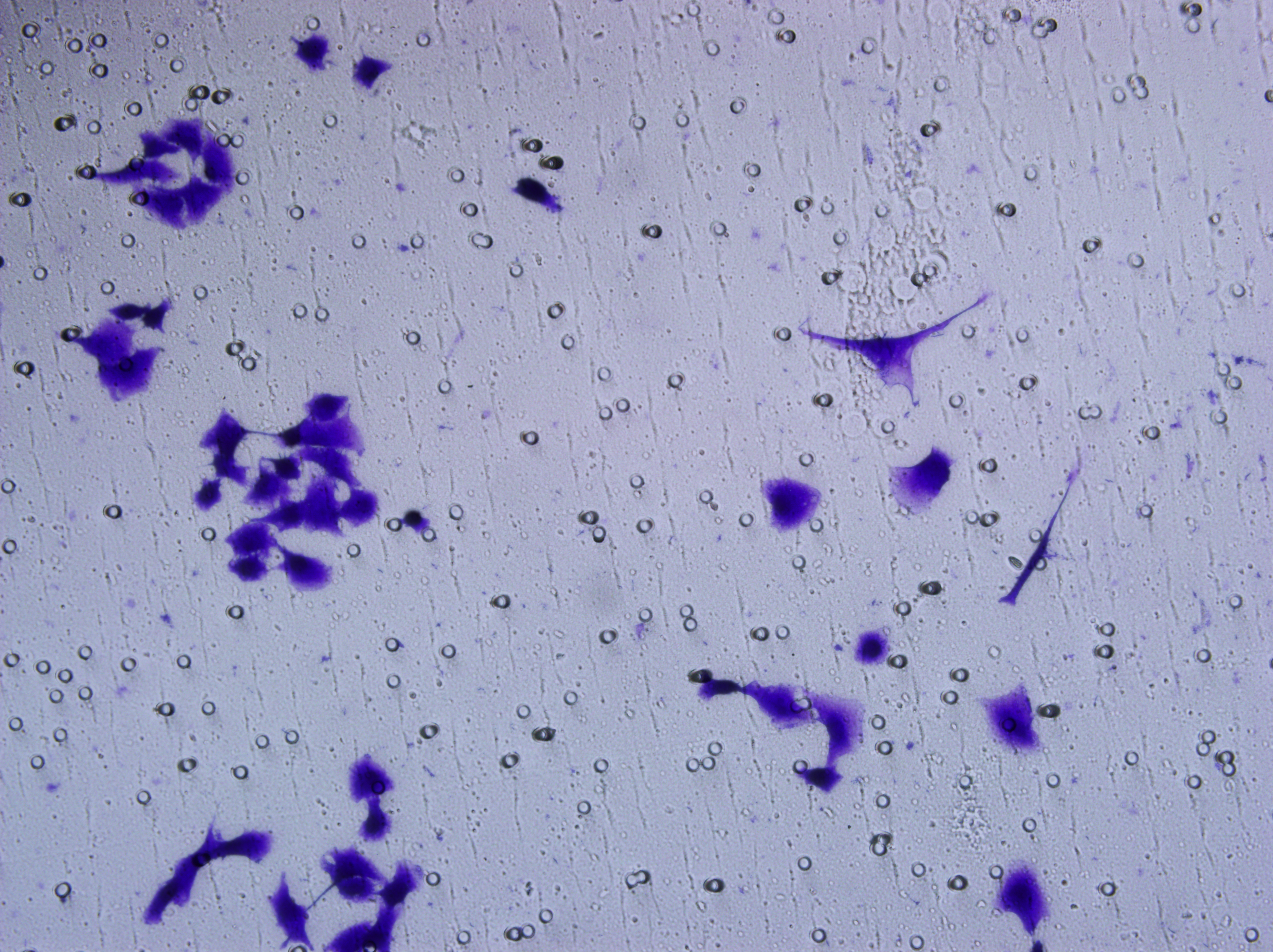

Supplement: Supplemental Information 14 [file peerj-12-18476-s014.png]

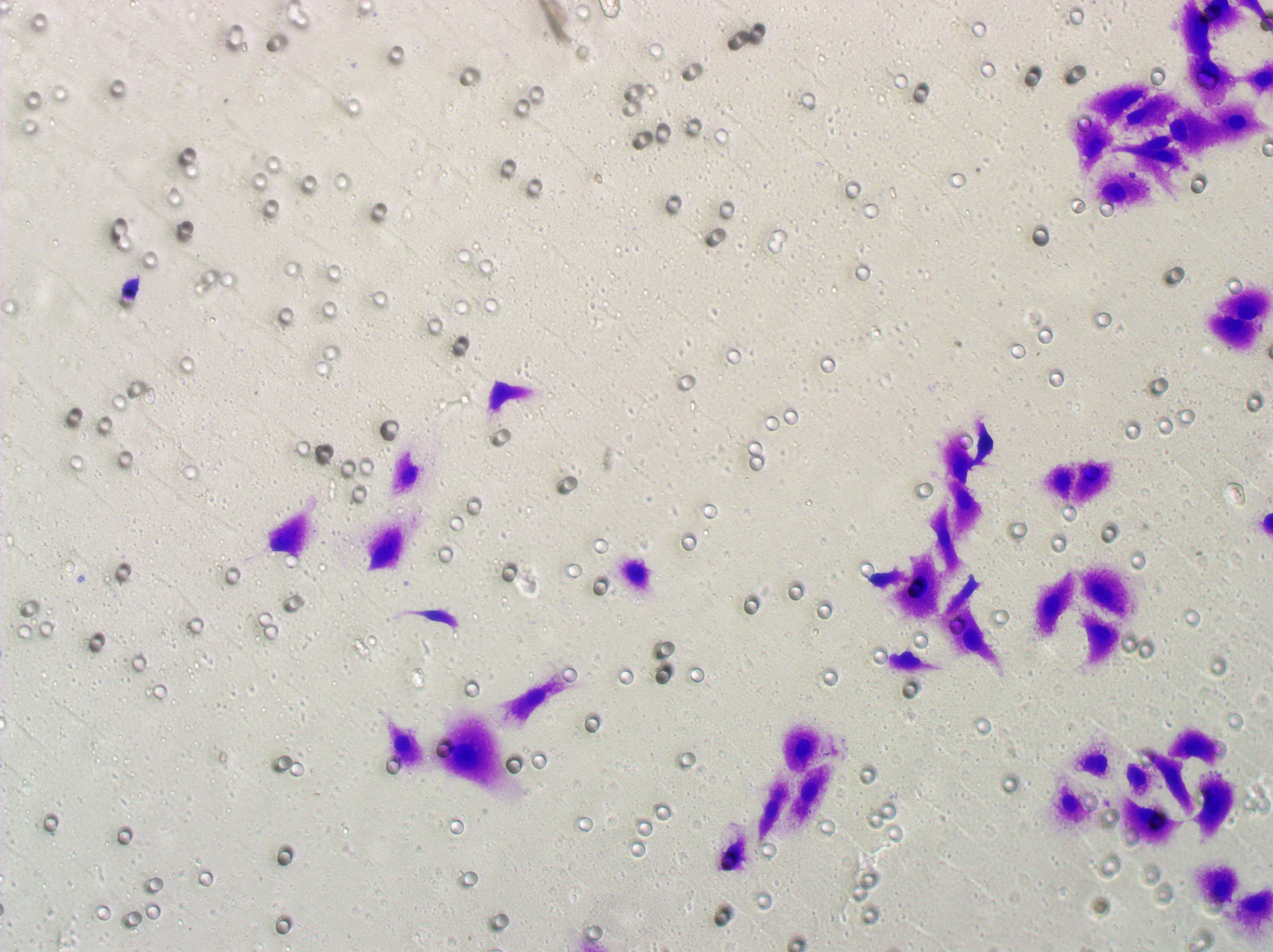

Supplement: Supplemental Information 15 [file peerj-12-18476-s015.png]

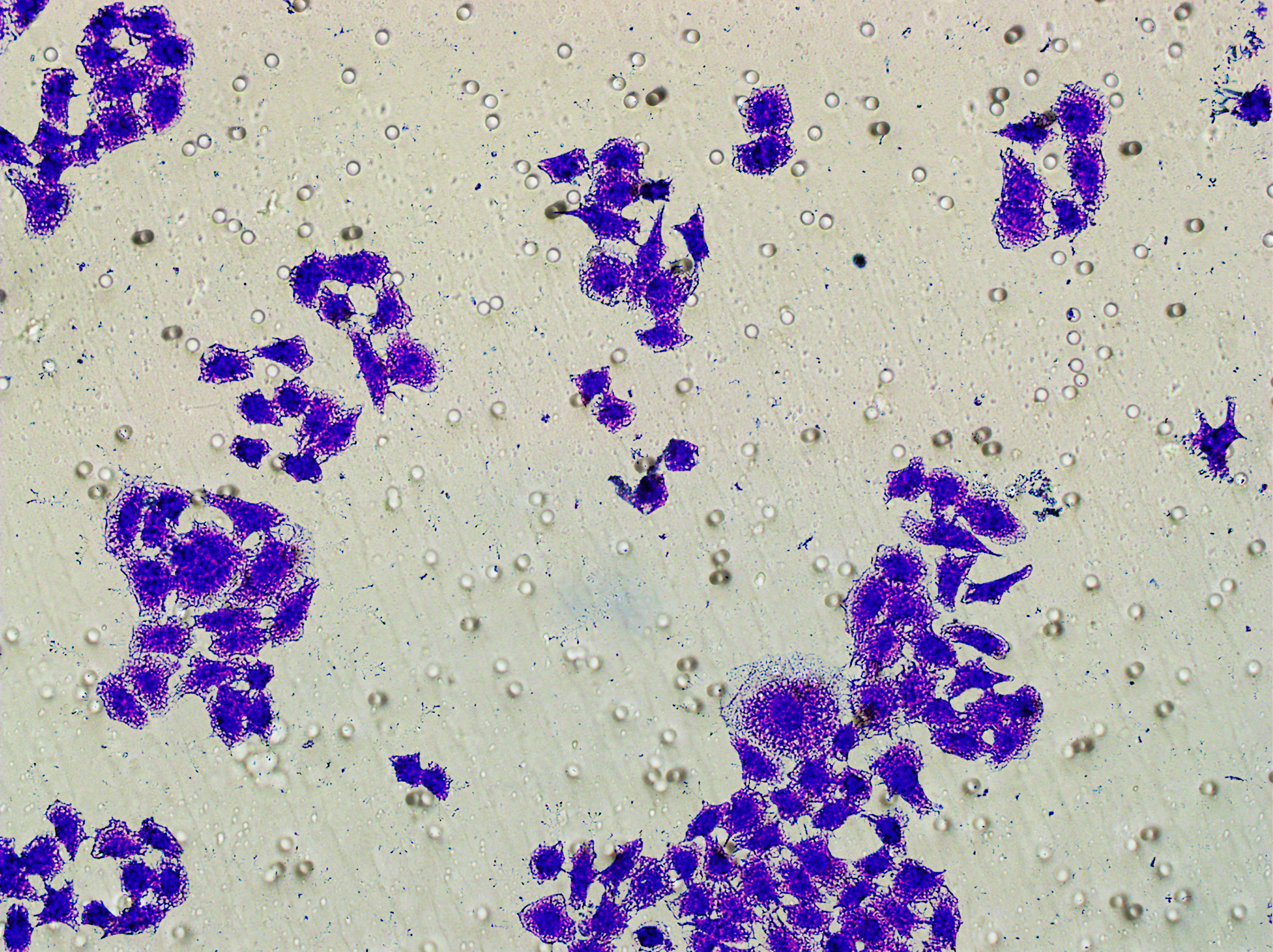

Supplement: Supplemental Information 16 [file peerj-12-18476-s016.png]

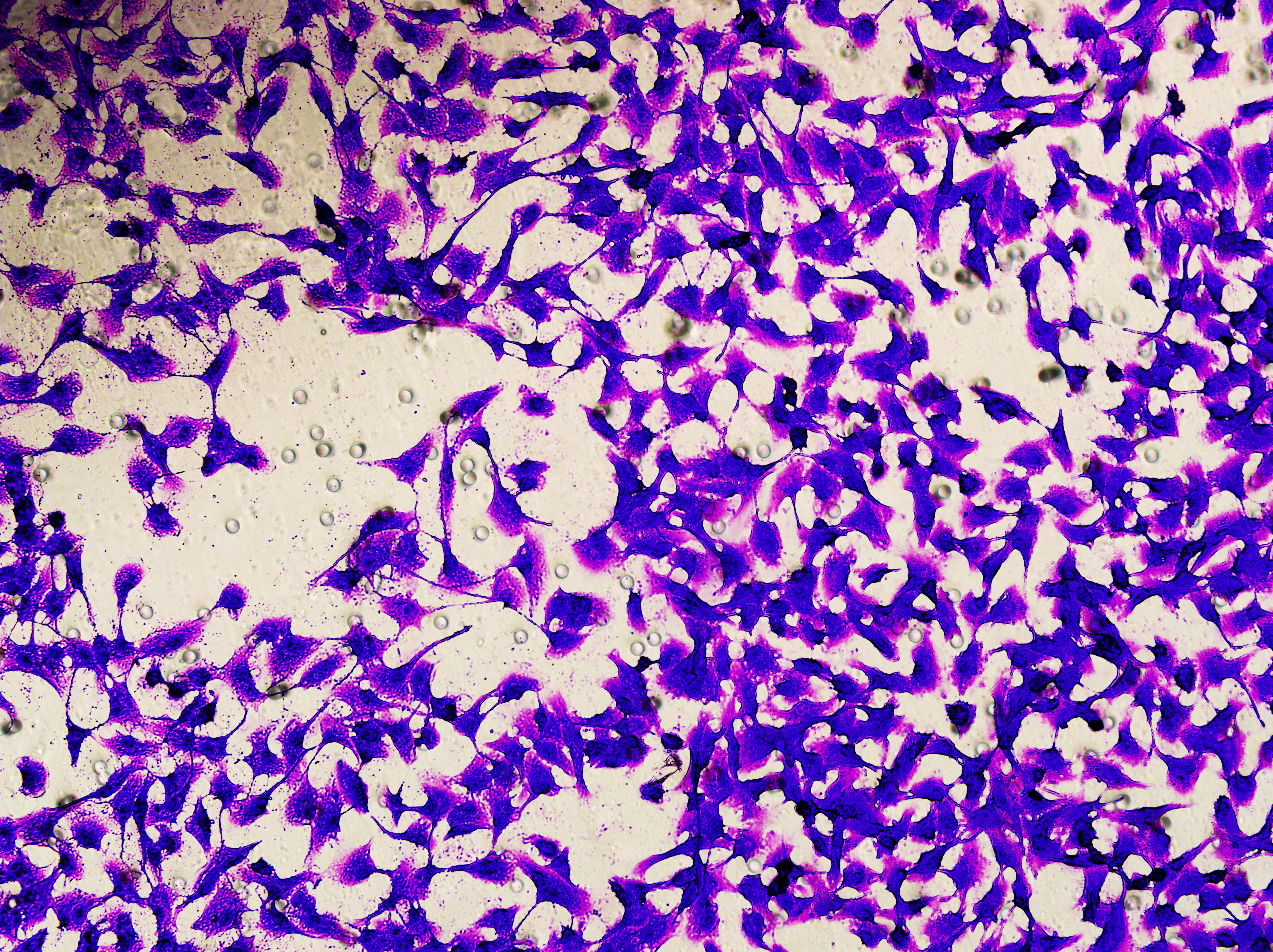

Supplement: Supplemental Information 17 [file peerj-12-18476-s017.png]

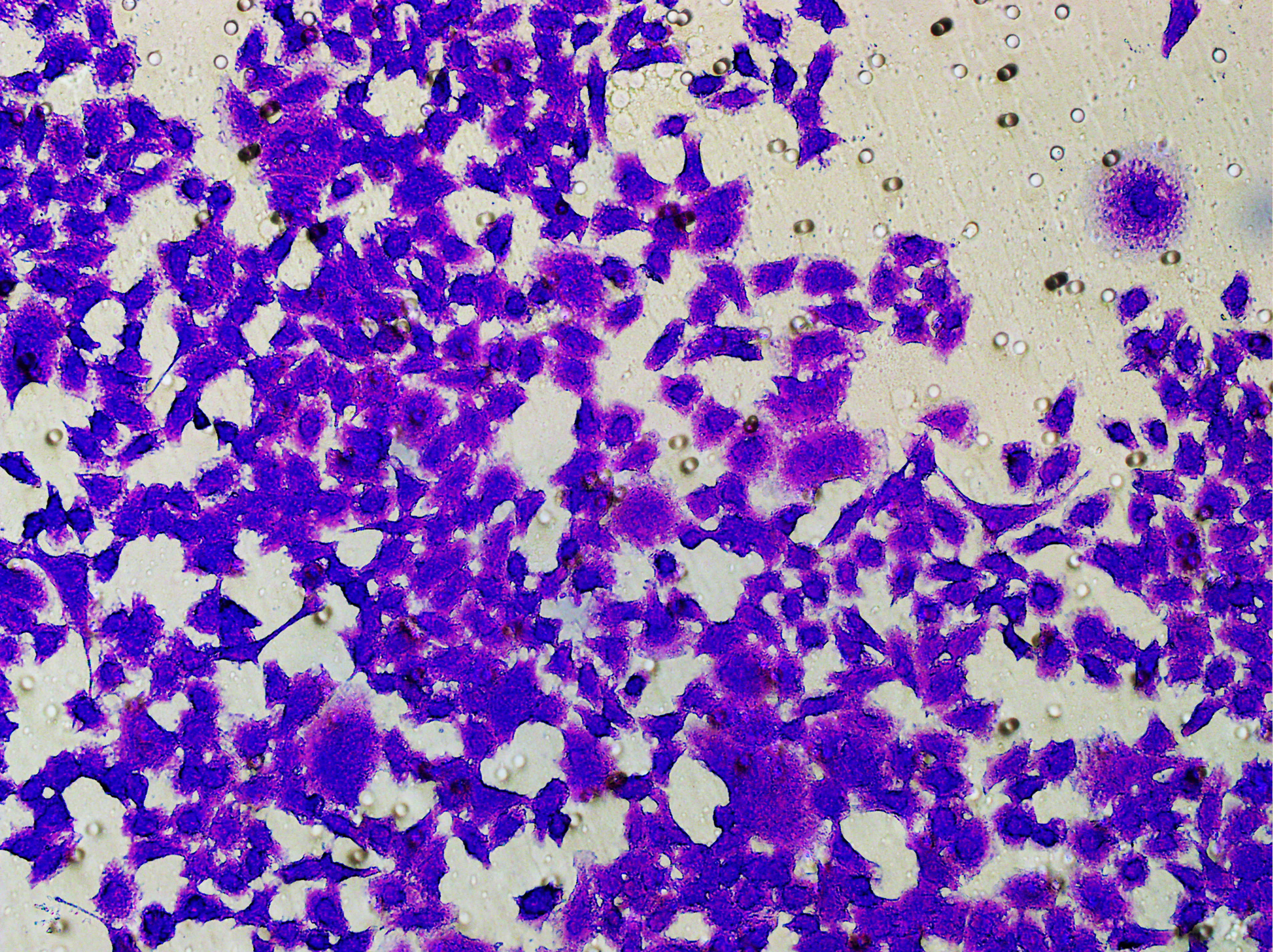

Supplement: Supplemental Information 18 [file peerj-12-18476-s018.png]

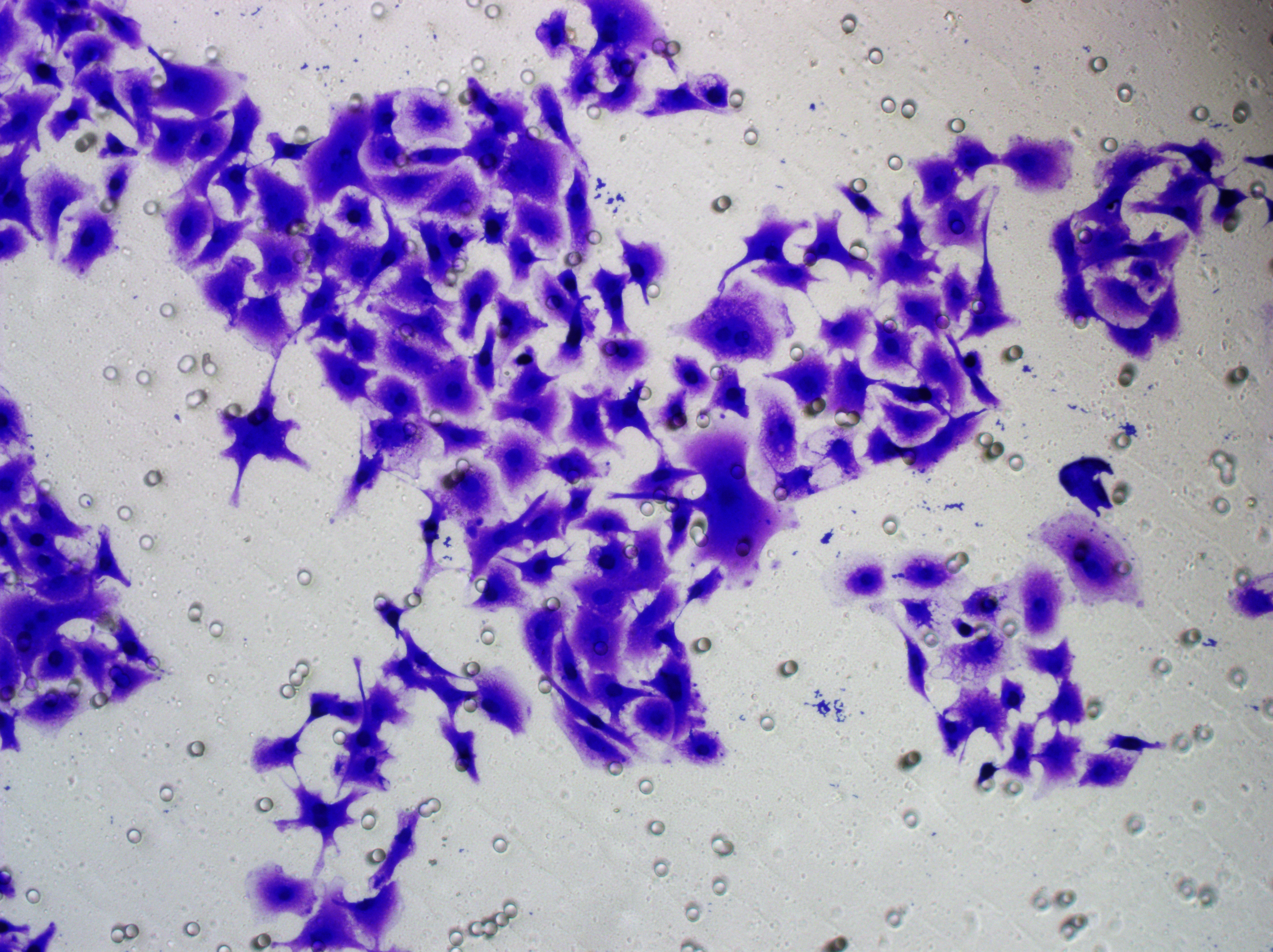

Supplement: Supplemental Information 19 [file peerj-12-18476-s019.png]

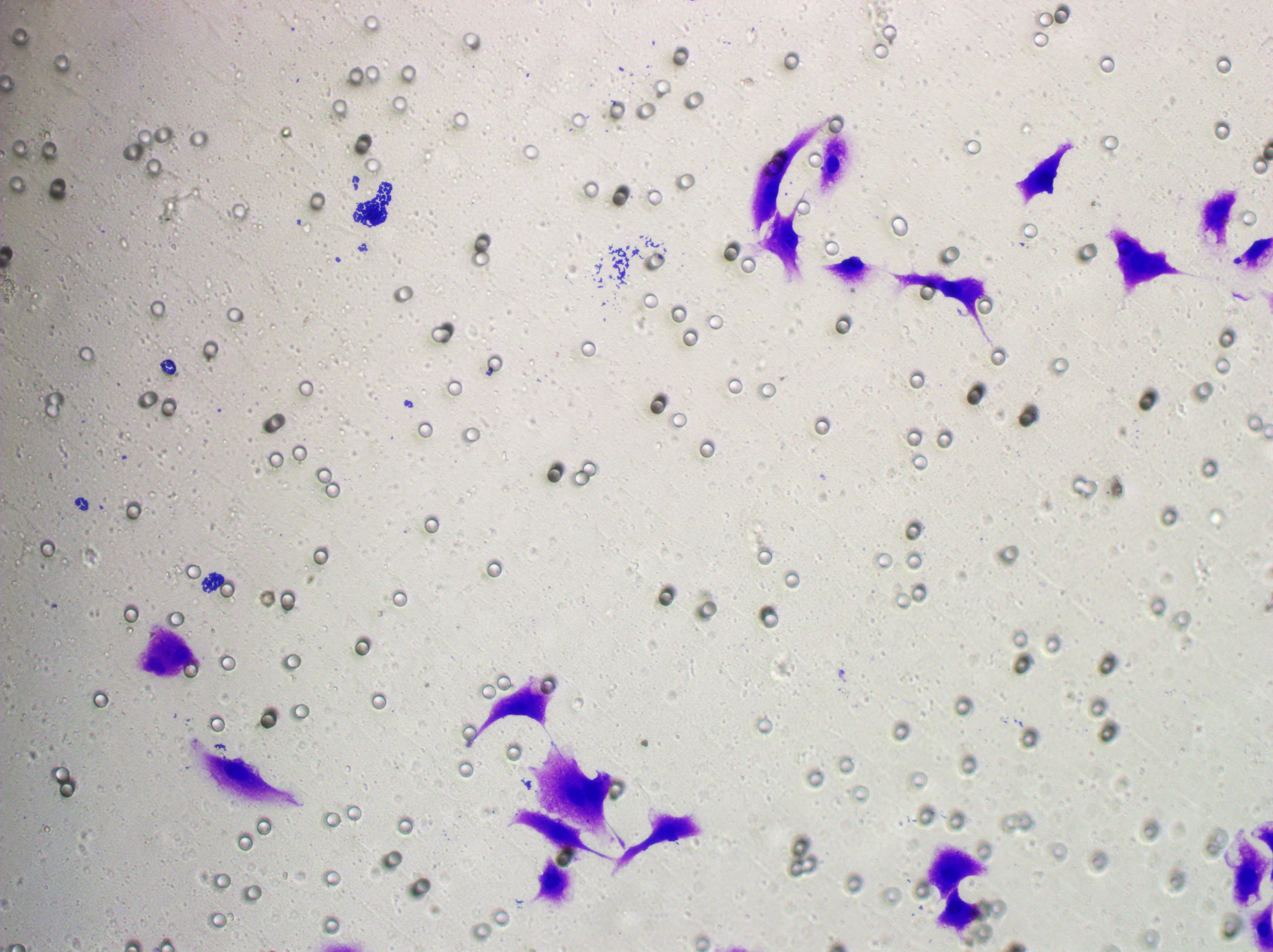

Supplement: Supplemental Information 20 [file peerj-12-18476-s020.png]

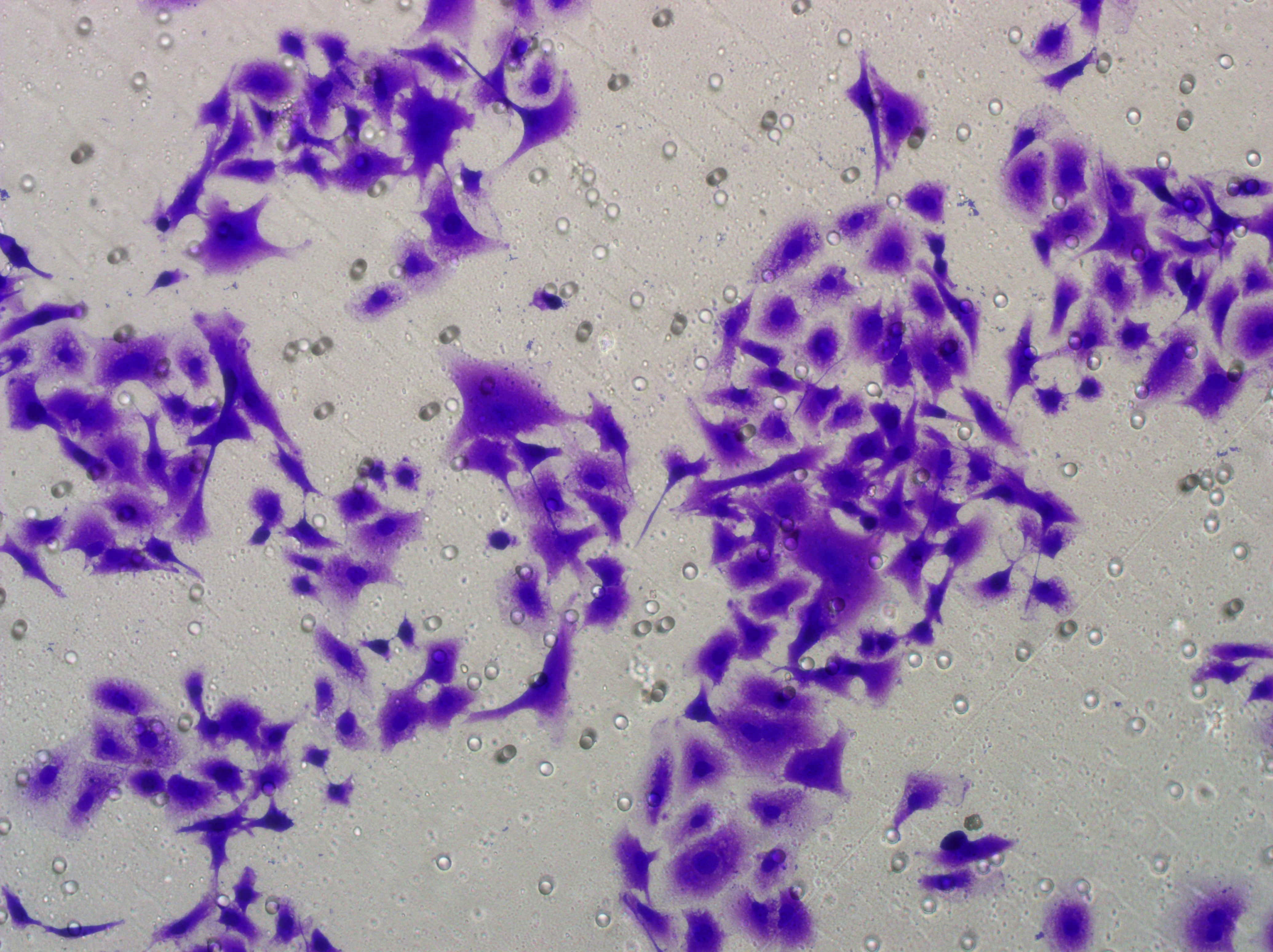

Supplement: Supplemental Information 21 [file peerj-12-18476-s021.png]

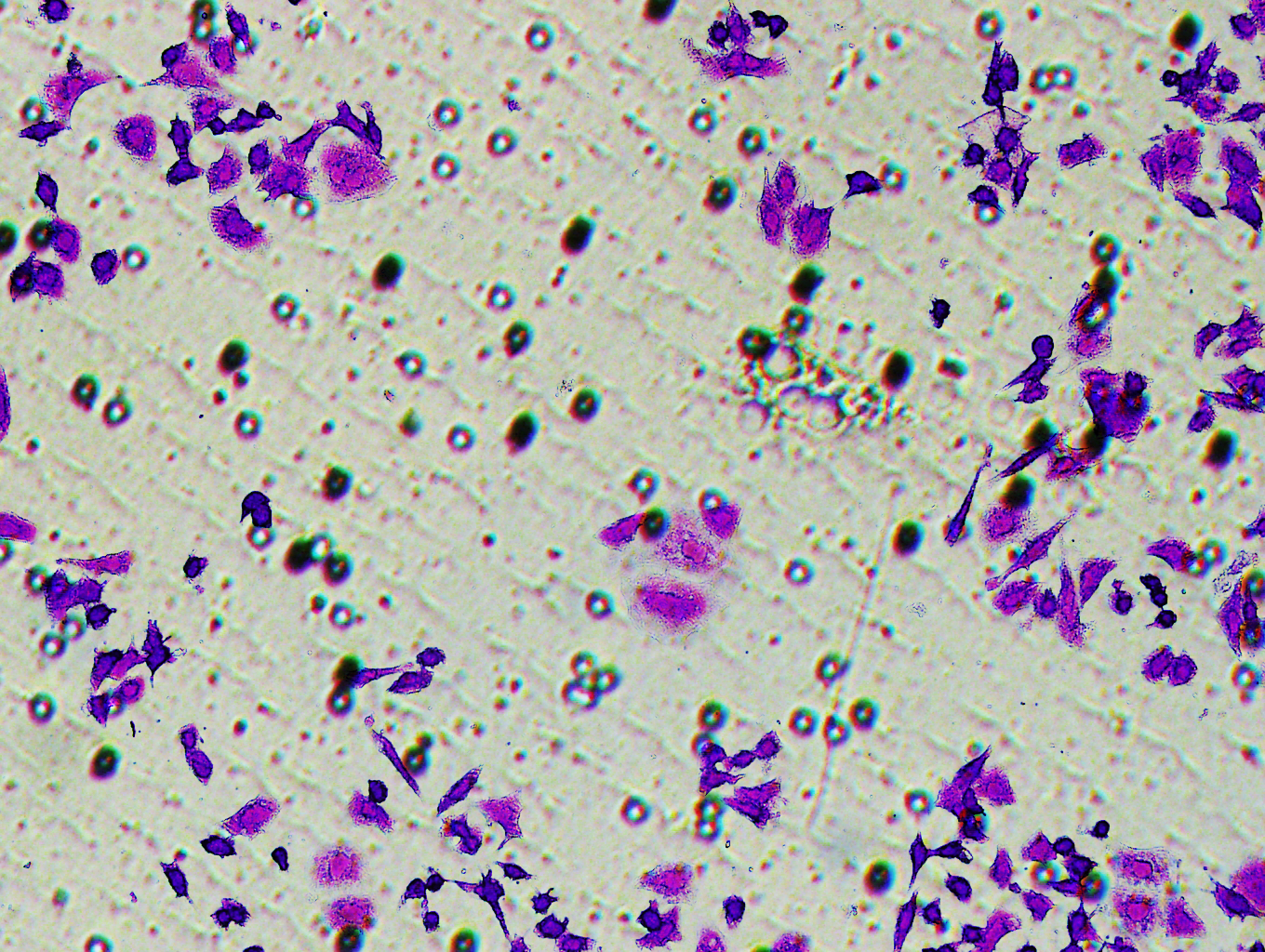

Supplement: Supplemental Information 22 [file peerj-12-18476-s022.png]

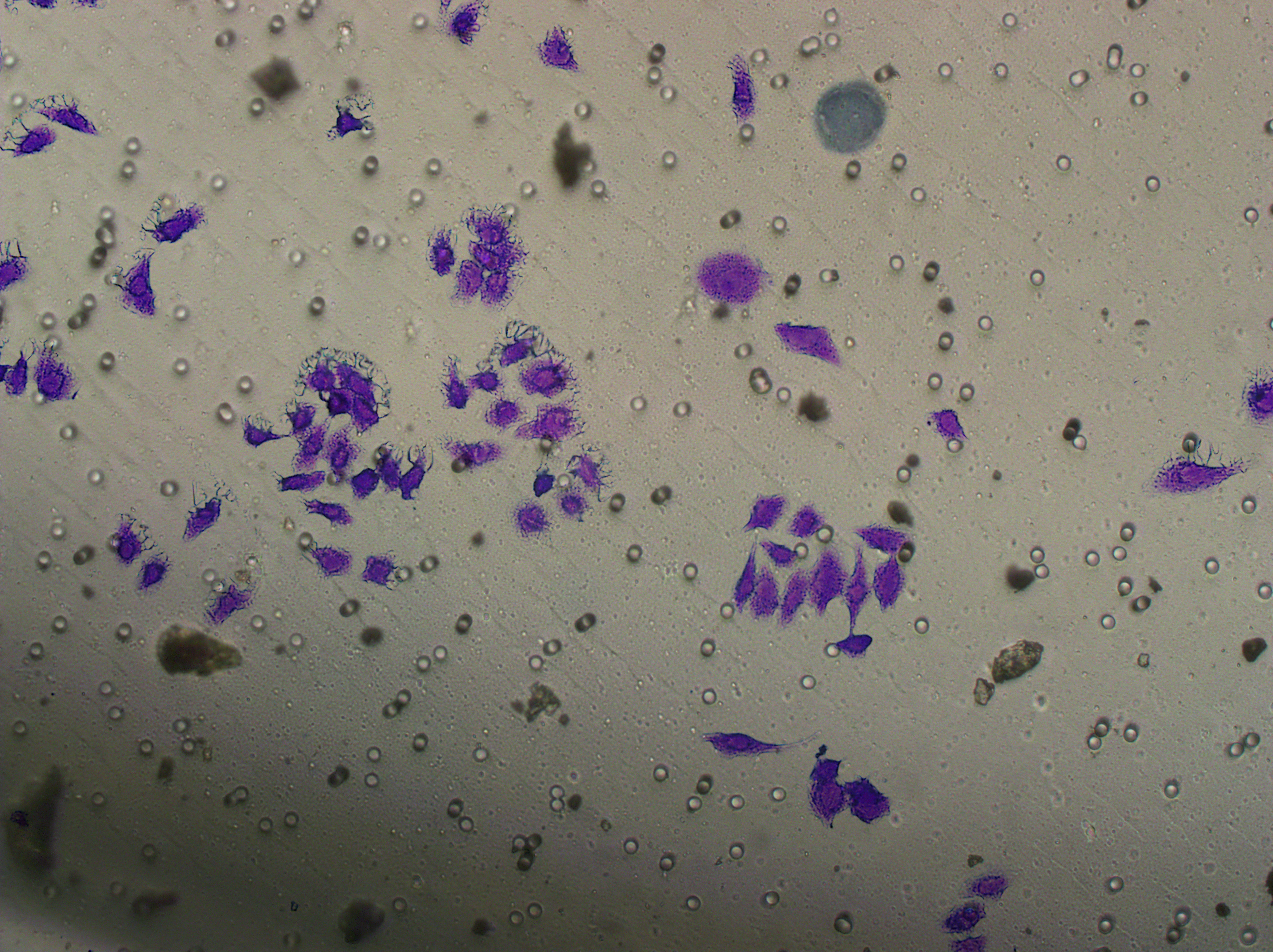

Supplement: Supplemental Information 23 [file peerj-12-18476-s023.png]

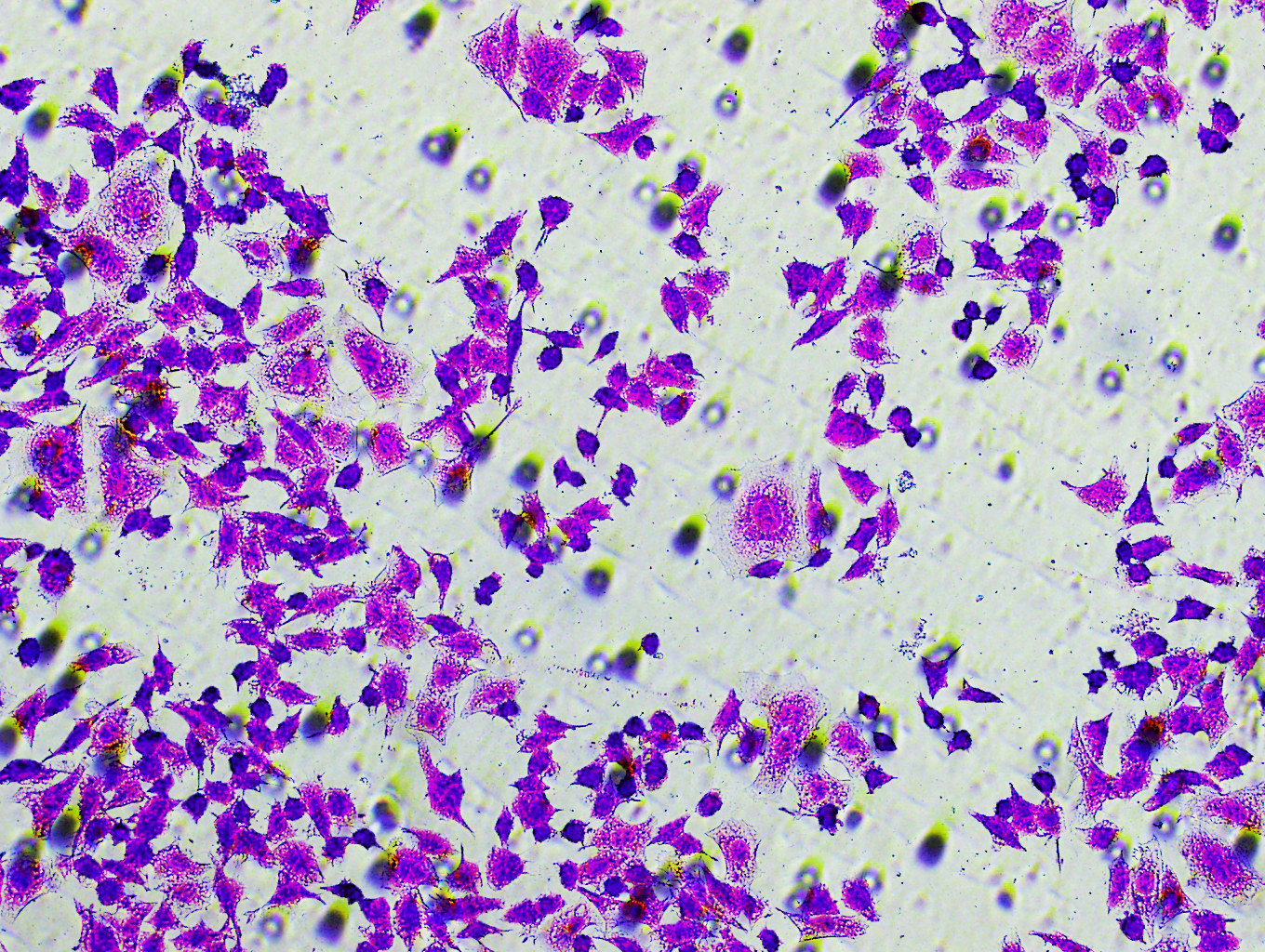

Supplement: Supplemental Information 24 [file peerj-12-18476-s024.png]

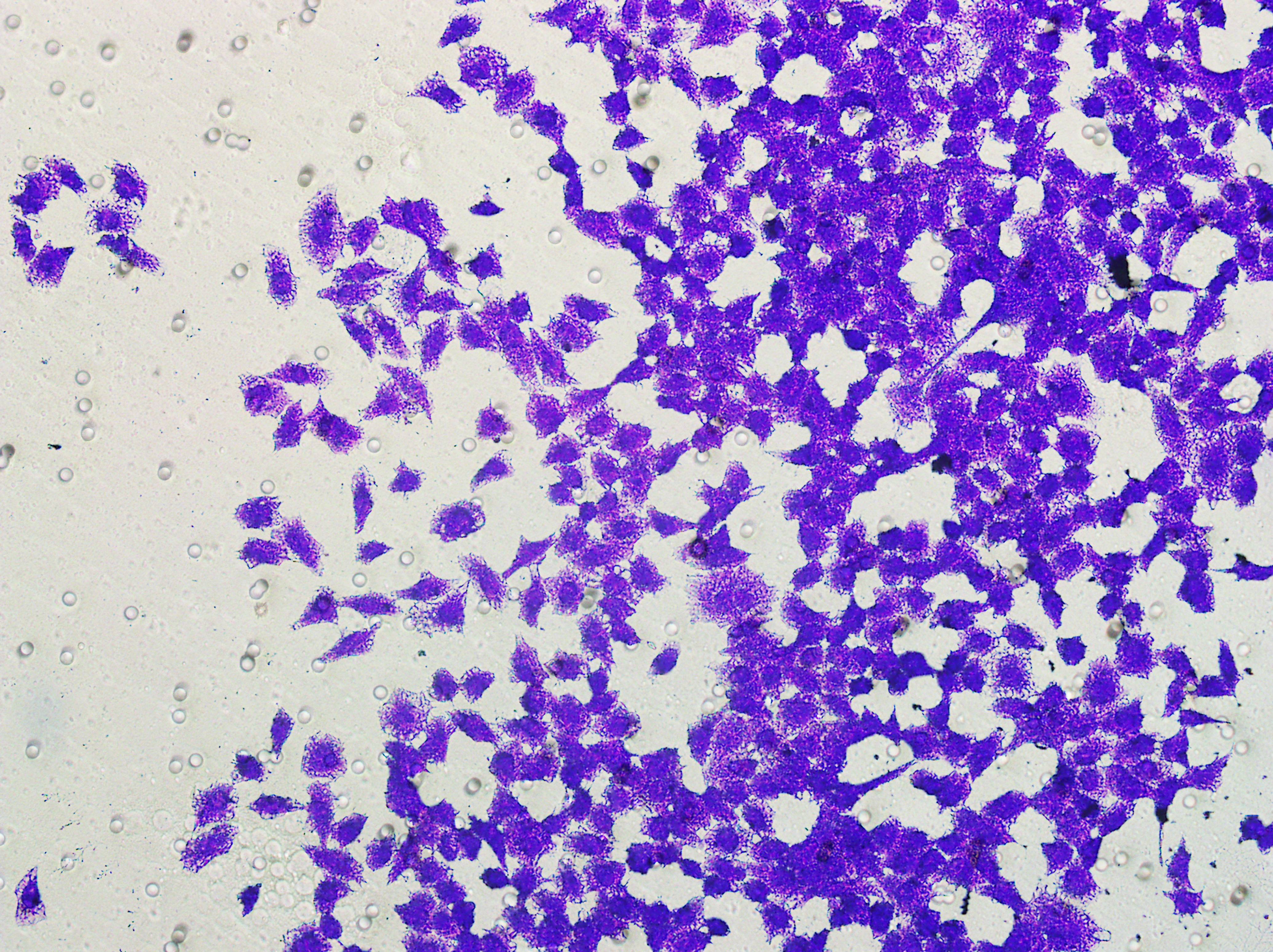

Supplement: Supplemental Information 25 [file peerj-12-18476-s025.png]

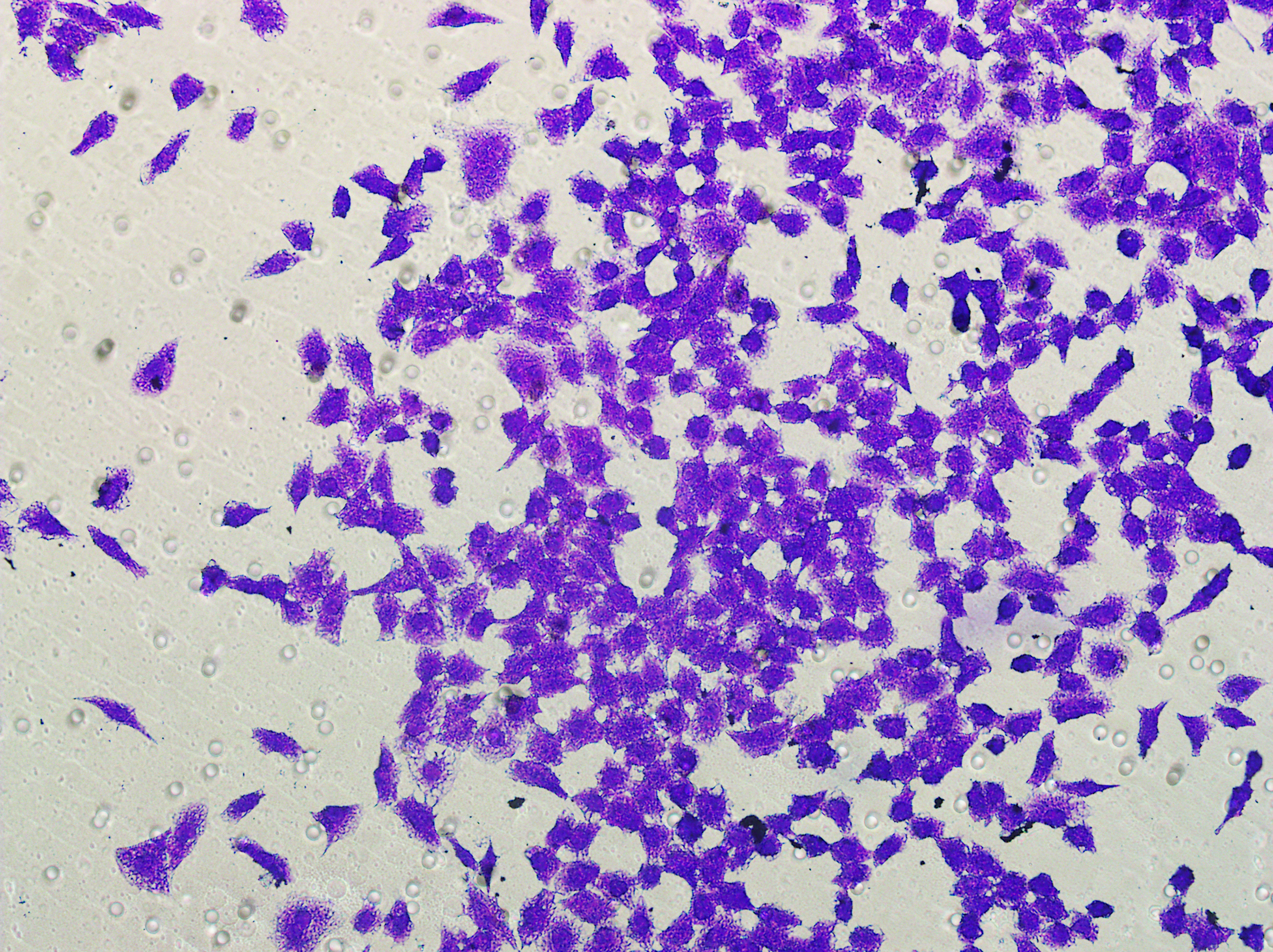

Supplement: Supplemental Information 26 [file peerj-12-18476-s026.png]

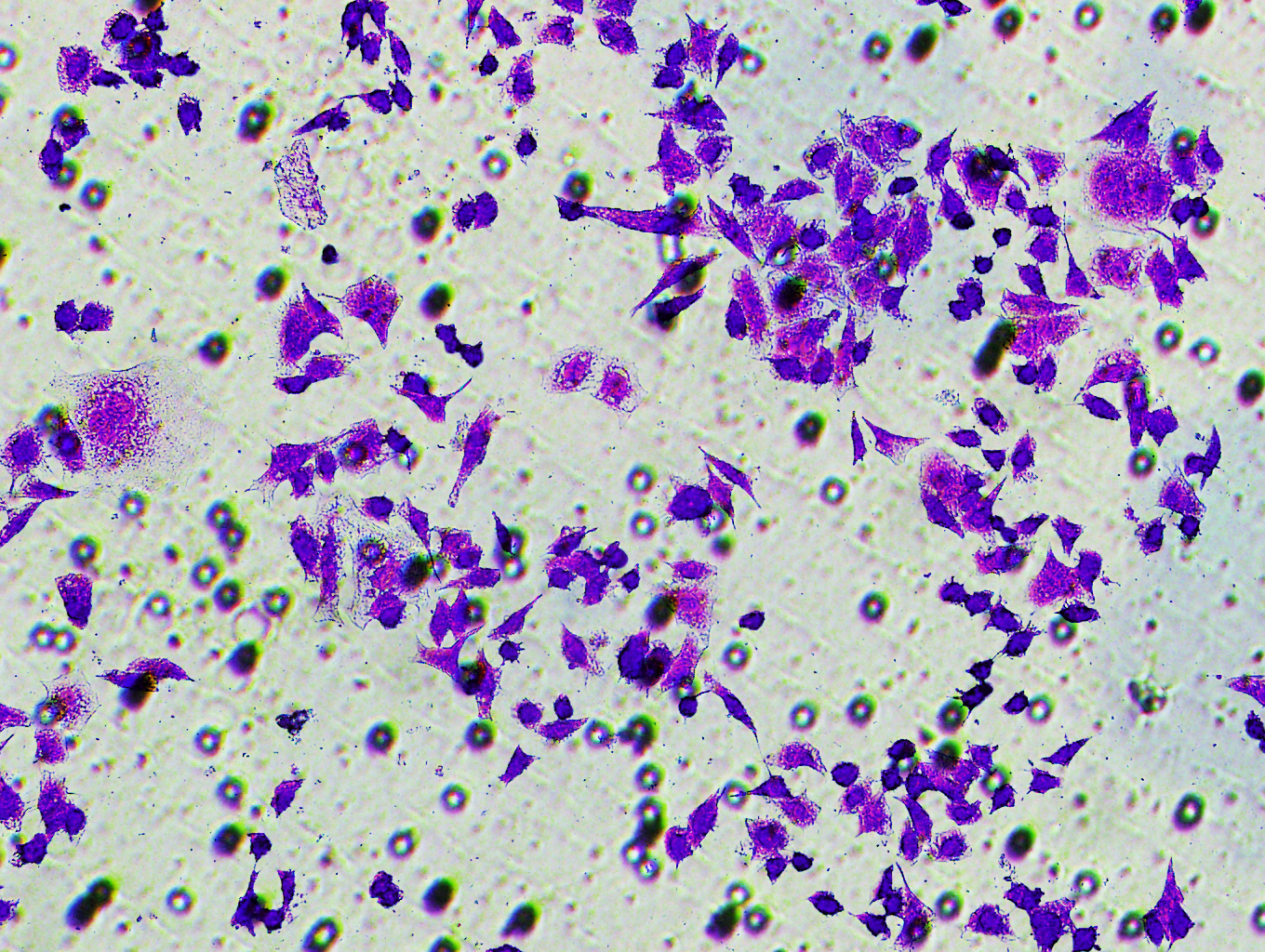

Supplement: Supplemental Information 27 [file peerj-12-18476-s027.png]

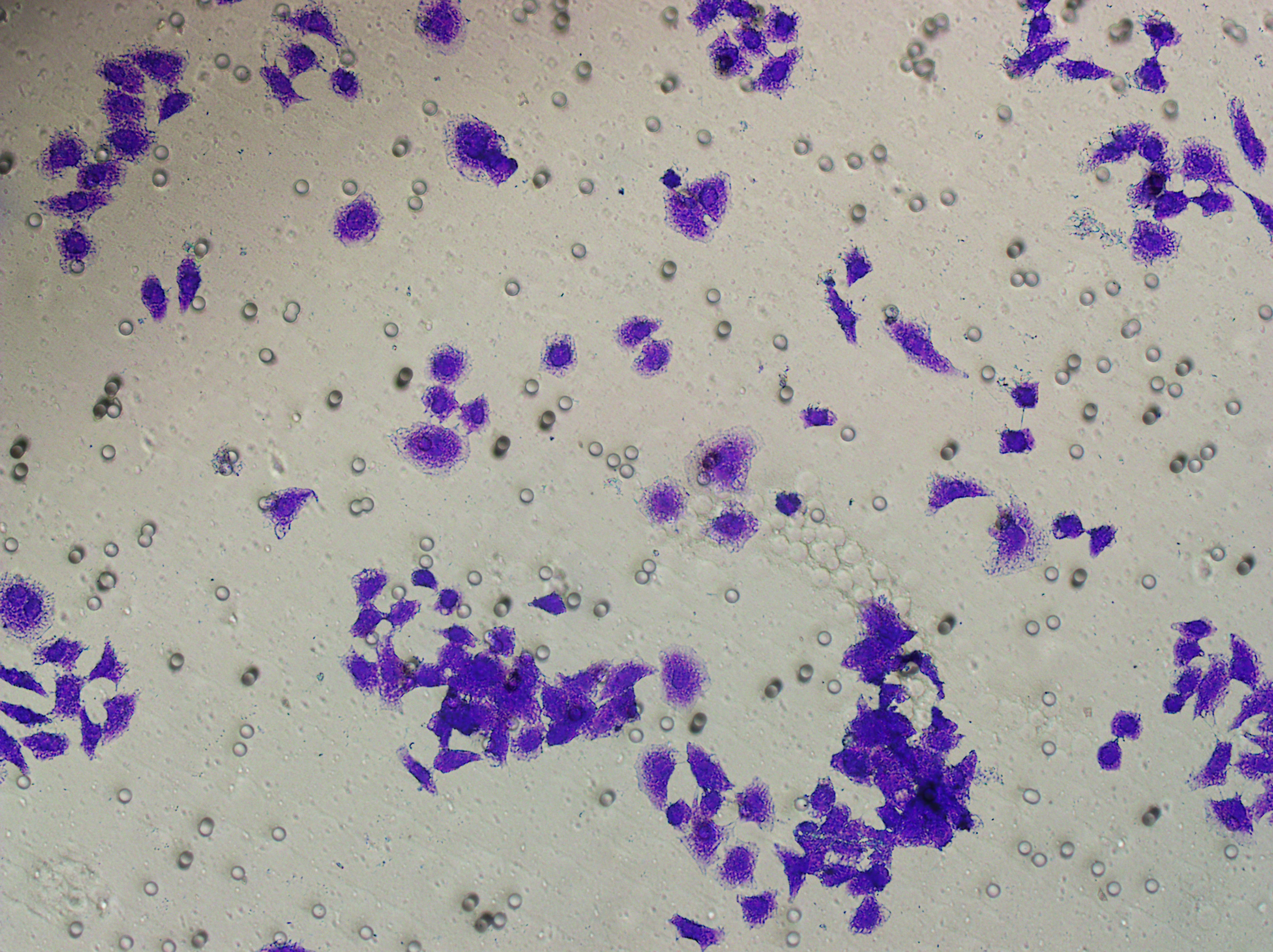

Supplement: Supplemental Information 28 [file peerj-12-18476-s028.png]

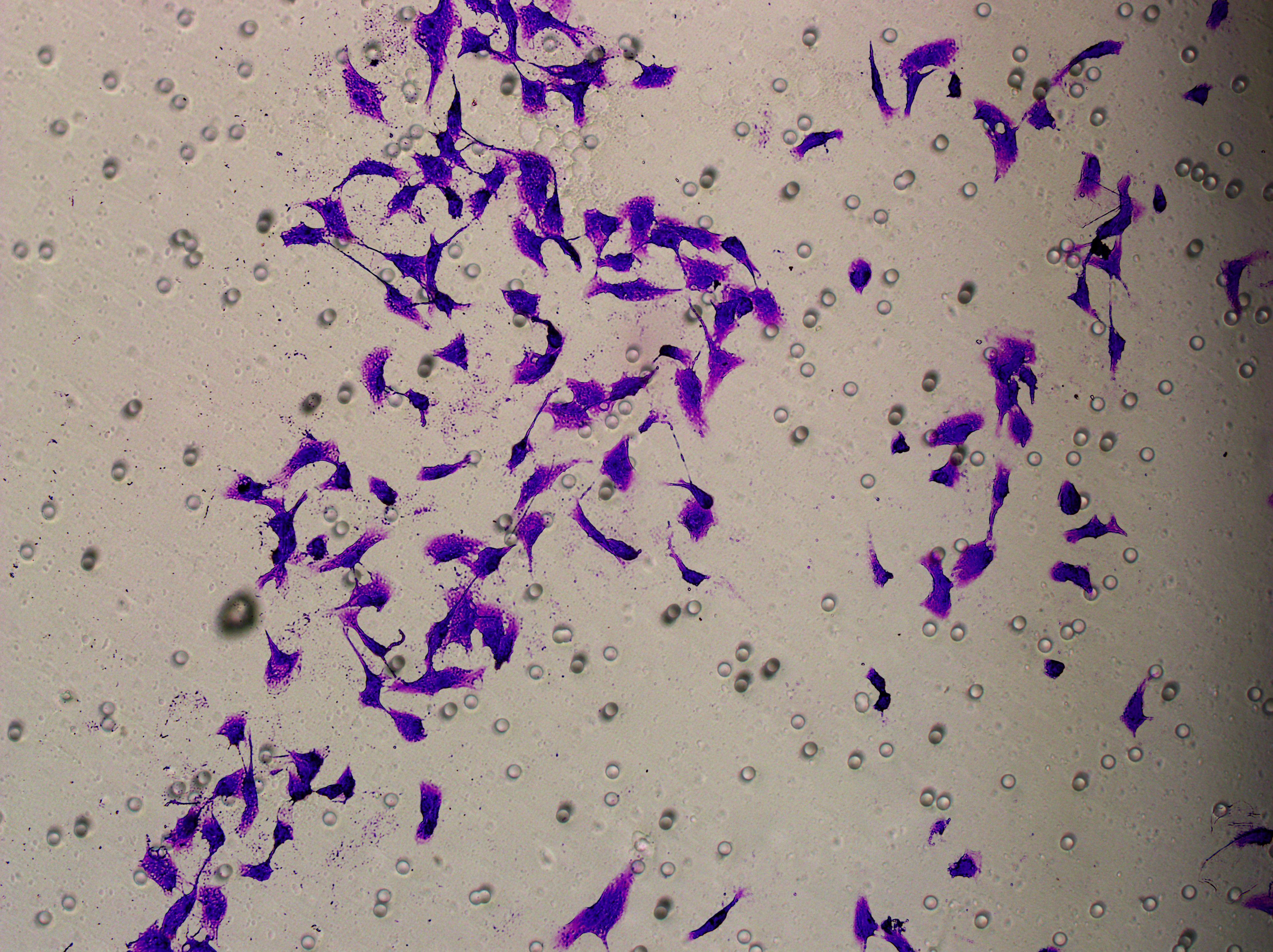

Supplement: Supplemental Information 29 [file peerj-12-18476-s029.png]

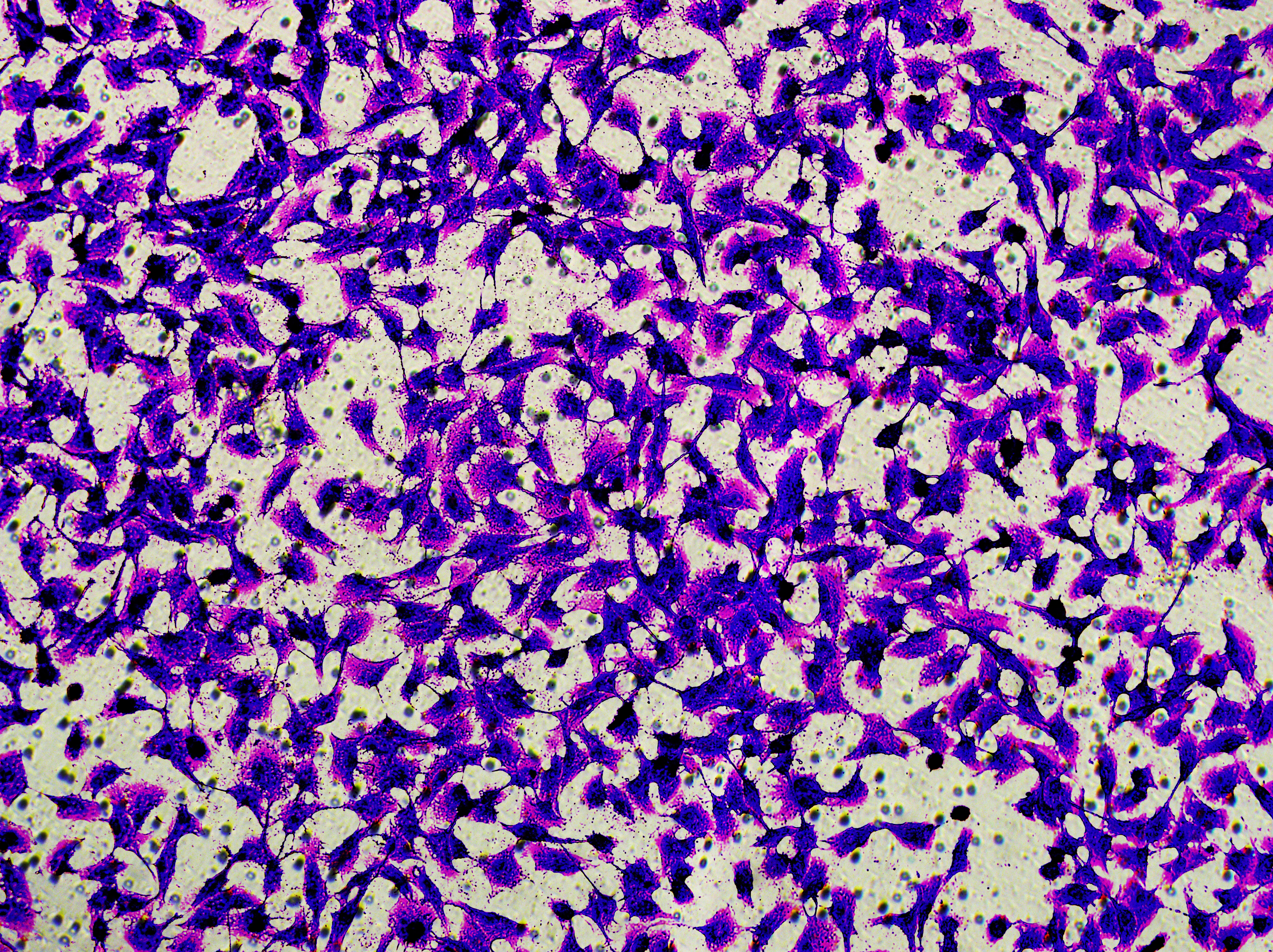

Supplement: Supplemental Information 30 [file peerj-12-18476-s030.png]

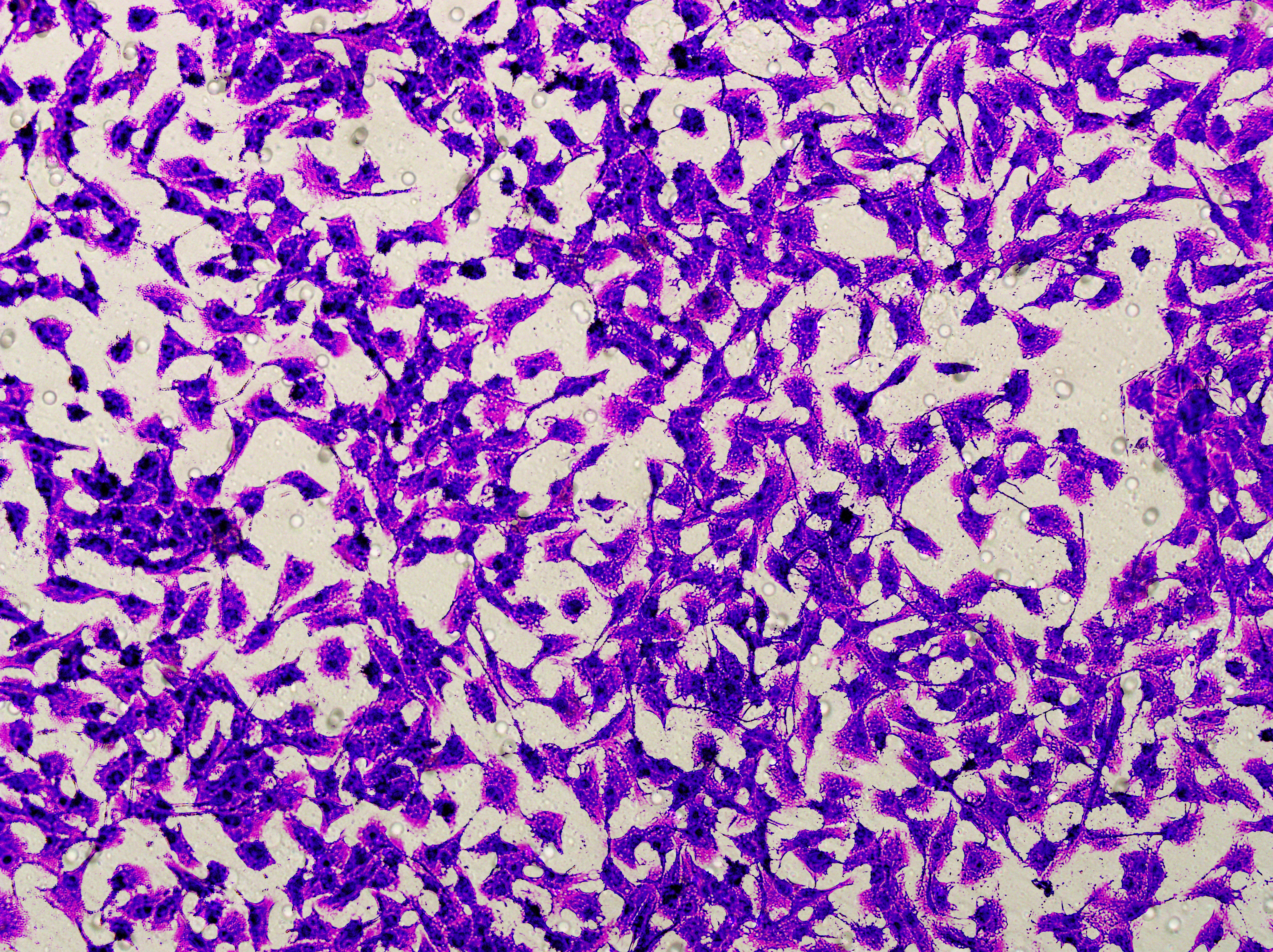

Supplement: Supplemental Information 31 [file peerj-12-18476-s031.png]

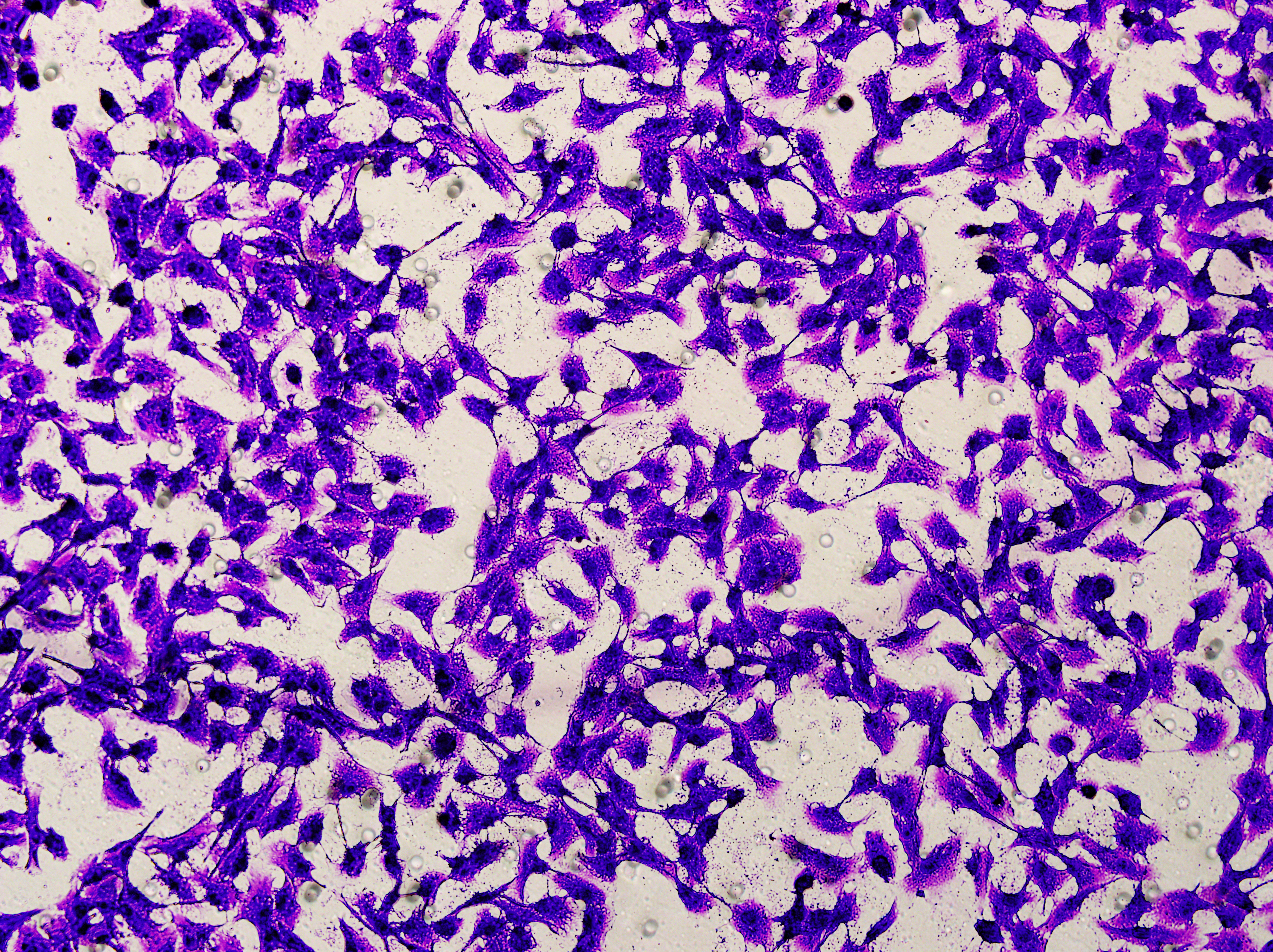

Supplement: Supplemental Information 32 [file peerj-12-18476-s032.png]

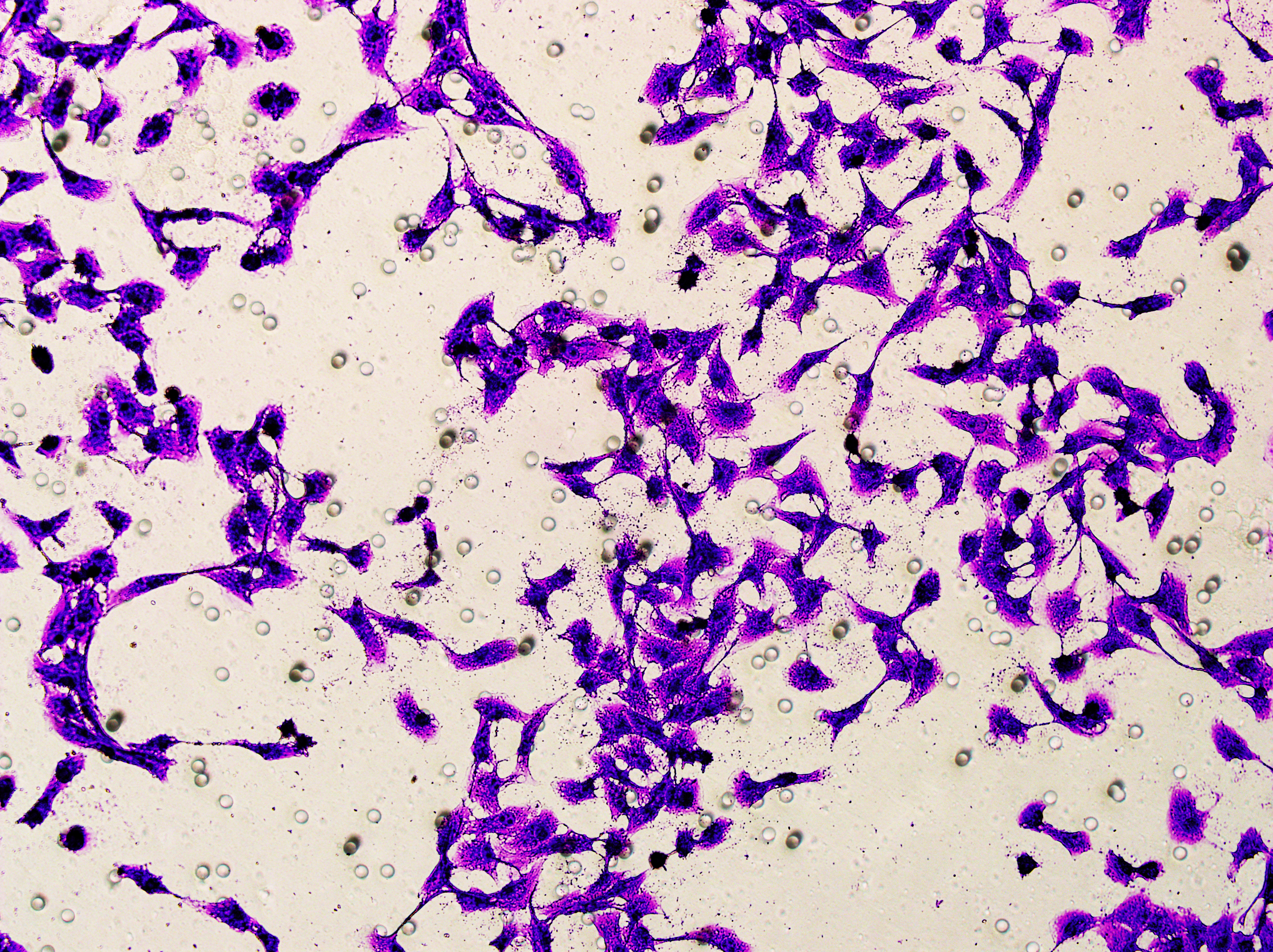

Supplement: Supplemental Information 33 [file peerj-12-18476-s033.png]

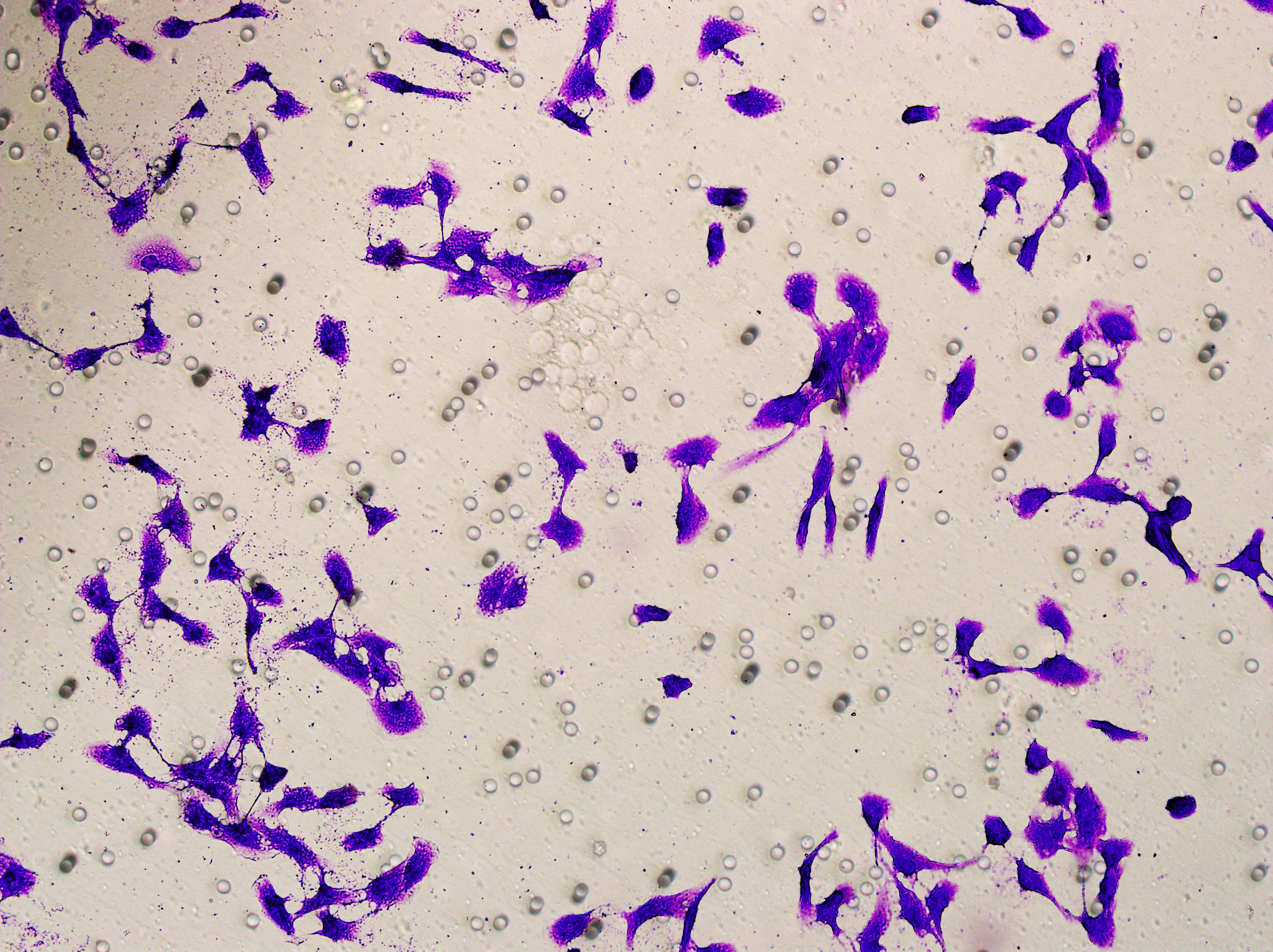

Supplement: Supplemental Information 34 [file peerj-12-18476-s034.png]

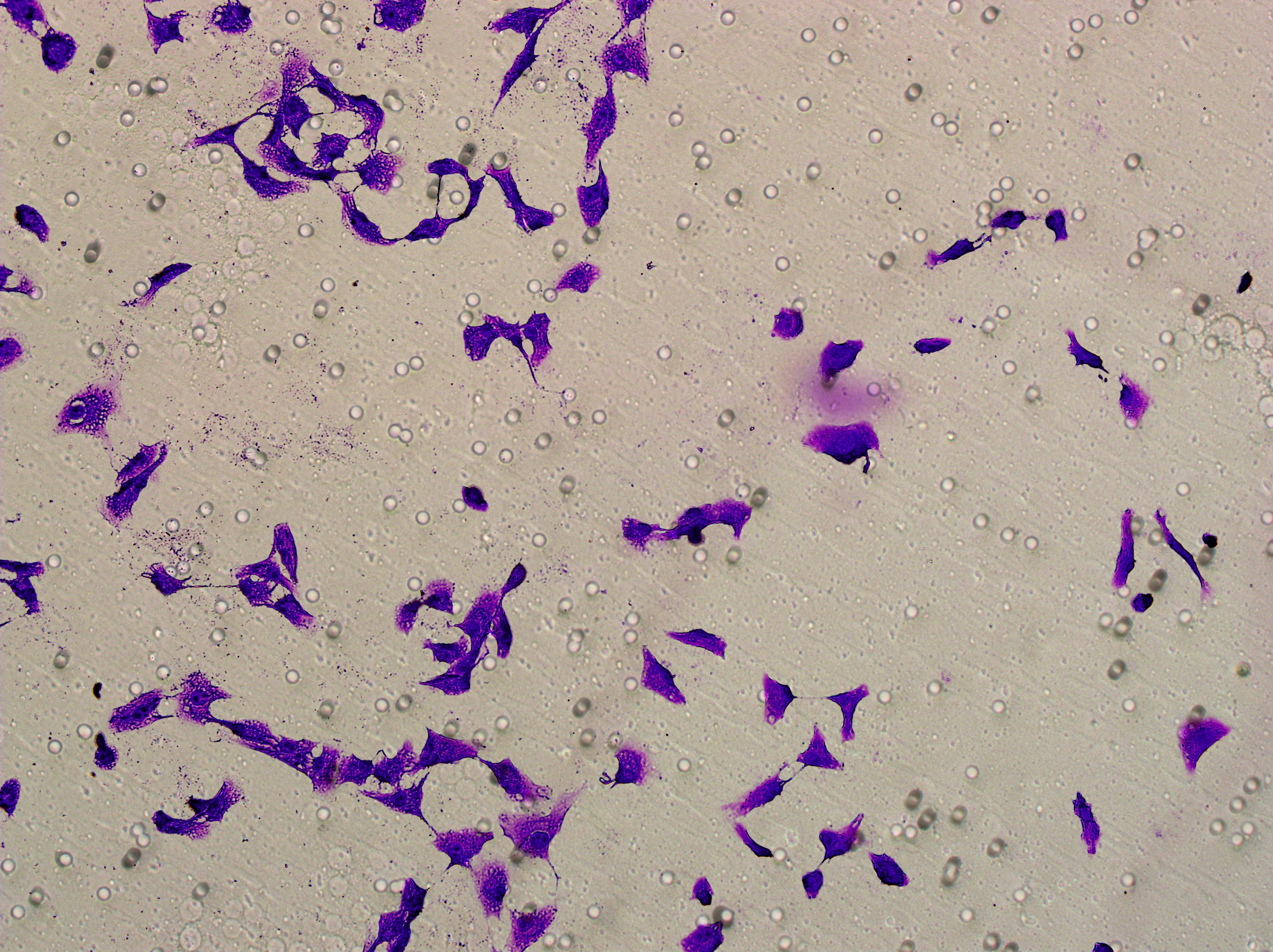

Supplement: Supplemental Information 35 [file peerj-12-18476-s035.png]

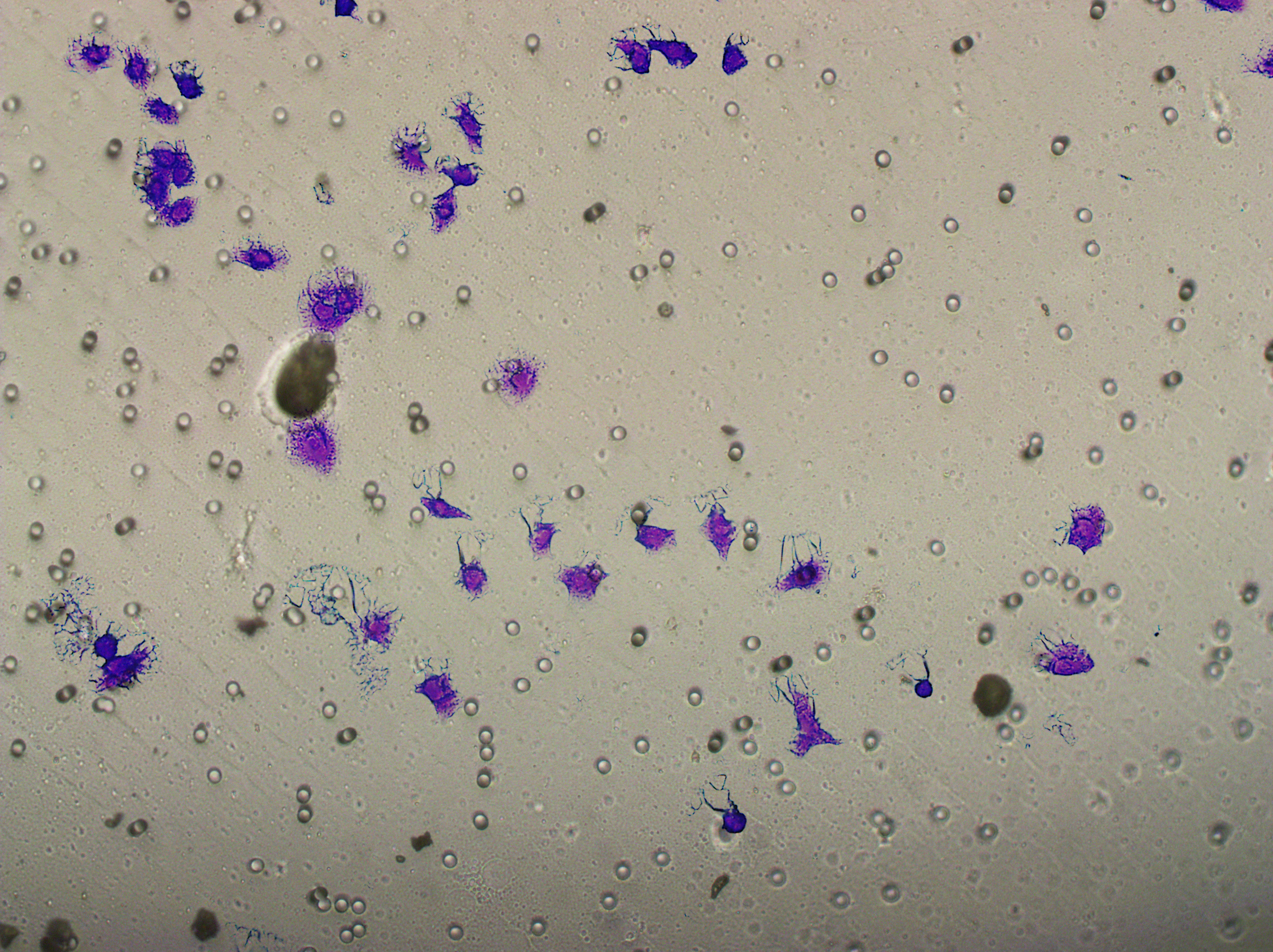

Supplement: Supplemental Information 36 [file peerj-12-18476-s036.png]

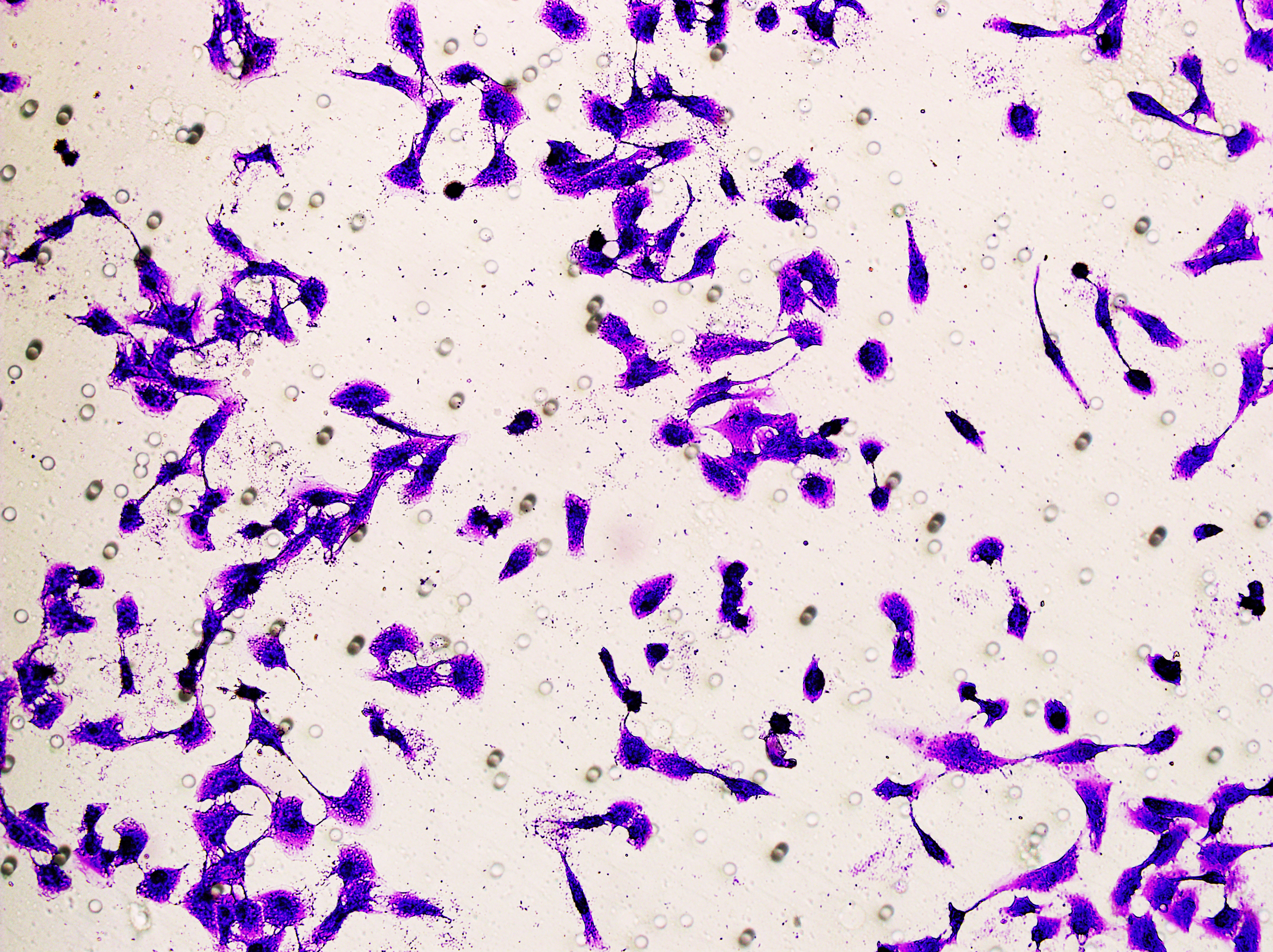

Supplement: Supplemental Information 37 [file peerj-12-18476-s037.png]
